# Biotransformation of the Phenolic Constituents from Licorice and Cytotoxicity Evaluation of Their Metabolites

**DOI:** 10.3390/ijms221810109

**Published:** 2021-09-18

**Authors:** Yina Xiao, Fubo Han, Ik-Soo Lee

**Affiliations:** College of Pharmacy, Chonnam National University, Gwangju 61186, Korea; yogurtxiao@163.com (Y.X.); hanfubo0306@gmail.com (F.H.)

**Keywords:** biotransformation, licorice, phenolic compounds, cytotoxicity

## Abstract

Biotransformation of four bioactive phenolic constituents from licorice, namely licoisoflavanone (**1**), glycyrrhisoflavone (**2**), echinatin (**3**), and isobavachalcone (**4**), was performed by the selected fungal strain *Aspergillus niger* KCCM 60332, leading to the isolation of seventeen metabolites (**5**–**21**). Structures of the isolated compounds were determined on the basis of extensive spectroscopic methods, twelve of which (**5**–**7**, **10**–**17** and **19**) have been previously undescribed. A series of reactions including hydroxylation, hydrogenation, epoxidation, hydrolysis, reduction, cyclization, and alkylation was observed in the biotransformation process. All compounds were tested for their cytotoxic activities against three different human cancer cell lines including A375P, MCF-7, and HT-29. Compounds **1** and **12** exhibited most considerable cytotoxic activities against all the cell lines investigated, while compounds **2** and **4** were moderately cytotoxic. These findings will contribute to expanding the chemical diversity of phenolic compounds, and compounds **1** and **12** may serve as leads for the development of potential cancer chemopreventive agents.

## 1. Introduction

Biotransformation can be defined as a specific modification (or modifications) of a chemical compound to a product with structural similarity by means of biological catalysts. A biological catalyst can be a whole microorganism or its enzyme, or other organisms [1,2,3,4]. Microorganisms can catalyze various reactions including hydroxylation, dehydrogenation, methylation, etc., to modify the chemical structure of a bioactive substrate resulting in the formation of metabolites which maintain the core structure of the substrate [5,6,7]. Biotransformation using microorganisms has advantages over conventional chemical synthesis due to its environmental acceptability, stereo- and regio-selectivity, and mild conditions [8]. It could be used as an alternative to chemical synthesis for generation and optimization of lead compounds in drug discovery and development [9]. Moreover, microorganisms possess the capability to mimic mammalian metabolism as their expression of enzymes that are homologous to phase I and II xenobiotic-metabolizing enzymes such as cytochrome P450 monooxygenases, UDP-glucuronosyltransferases, aryl sulfotransferases, and glutathione *S*-transferases [10,11,12].

Licorice is one of the most popular medicinal plants that has been used as a remedy for cough, gastric ulcer, inflammation, abdominal pain, cardiovascular diseases, and cancer since ancient time [13]. The major constituent glycyrrhizin and its aglycone glycyrrhetinic acid are also used in modern medicine. On the other hand, the phenolic constituents of licorice have been implicated in health-beneficial effects, particularly in stomach ulcers. It has been reported that the phenolic constituents of licorice have pharmacological effects for health [14]. However, little is known on the biotransformation and cytotoxicity of these health promoting phenolic compounds.

In this study, two licorice isoflavonoids licoisoflavanone (**1**) and glycyrrhisoflavone (**2**) were isolated from licorice, and two licorice chalcones echinatin (**3**) and isobavachalcone (**4**) were synthesized. To expand the diversity of phenolic compounds, **1**–**4** were subject to biotransformation. Twelve new (**5**–**7**, **10**–**17** and **19**) and five known (**8**, **9**, **18**, **20** and **21**) metabolites were identified and their structures were characterized by spectroscopic methods. All the compounds were evaluated for their cytotoxic activities against human cancer cell lines A375P, A549 and MCF-7.

## 2. Results and Discussion

### 2.1. Biotransformation of Two Isoflavonoids, Licoisoflavanone (**1**) and Glycyrrhisoflavone (**2**) with A. niger KCCM 60332

**Figure 1:**
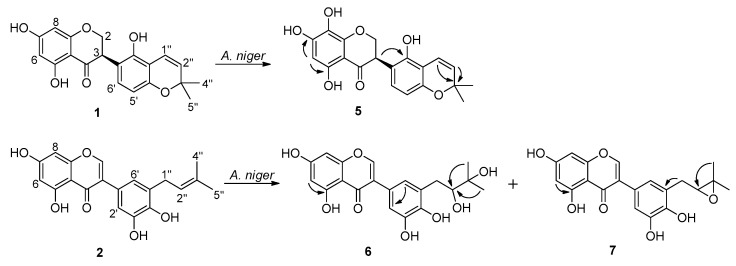
Compounds **5**–**7** obtained by biotransformation of **1** and **2** with *A. niger*. Selected HMBC correlations (^1^H→^13^C) of compounds **5**–**7** are indicated by arrows.

Biotransformation of licoisoflavanone (**1**) by the selected fungal strain *A. niger* KCCM 60332 produced one new hydroxylated metabolite **5**. Biotransformation of glycyrrhisoflavone (**2**) furnished one new dihydroxylated (**6**) and one new epoxidized (**7**) metabolites (Figure 1).

Compound **5** was obtained as a pale yellow amorphous powder. Its molecular formula was determined as C_20_H_18_O_7_ by an [M + Na]^+^ peak at *m*/*z* 393.0953 (calcd for C_20_H_18_O_7_Na, 393.0950) based on its HRESIMS spectrum, indicating that one oxygen atom was inserted into compound **1**. The ^1^H-NMR spectrum of **5** demonstrated eight one-proton signals (δ_H_ 6.91, 6.69, 6.34, 5.99, 5.68, 4.65, 4.53 and 4.23) and two methyl (δ_H_ 1.40) signals (Table 1). The ^13^C-NMR spectrum exhibited signals for twenty carbons. Except for the absence of one phenolic proton signal at C-8 and the presence of one downfield-shifted carbon signal at δc 125.3 in **5**, the ^1^H- and ^13^C-NMR data of **5** closely resembled those of **1** [15]. The mono-hydroxylation took place at C-8 based on the correlations from H-6 (δ_H_ 5.99) to C-5 (δc 156.0), C-7 (δc 157.0), and C-8 (δc 125.3) in the HMBC spectrum of **5**. The absolute configuration of **5** was determined to be *R* by the observation of positive Cotton effect at the range between 280 and 350 nm in the CD spectrum of **5** (Figure S15) [16]. Thus, structure of compound **5** was established as (3*R*)-5,7,8,2’-tetrahydroxy-6”,6”-dimethyl-(2”,3”:4’,3’)-pyranoflavanone.

Compound **6** was obtained as a yellow amorphous powder. HRESIMS of **6** showed an [M + Na]^+^ peak at *m*/*z* 411.1060 (calcd for C_20_H_20_O_8_Na, 411.1056) which established its molecular formula as C_20_H_20_O_8_ which corresponds to a dihydroxylated metabolite of **2**. The ^1^H- and ^13^C-NMR spectral data revealed twenty carbon signals consisting of two methyl groups, one methylene, six methines, ten quaternary carbons, and one carbonyl quaternary carbon. There were no olefinic proton signals of the prenyl moiety in the ^1^H-NMR spectrum of **6**. Moreover, the HMBC spectrum showed correlations between the two methyl protons (δ_H_ 1.25) and the two oxygen-bearing carbons at δ_C_ 79.2 and 72.5 indicating the dihydroxylation took place at the olefinic double bond of the prenyl group. On the basis of these evidences, structure of compound **6** was elucidated as 5,7,3′,4′-tetrahydroxy-5′-(2,3-dihydroxy-3-methylbutyl)isoflavone.

Compound **7** was obtained as a yellow amorphous powder. The HRESIMS spectrum of **7** exhibited an [M + Na]^+^ peak at *m*/*z* 393.0951 (calcd for C_20_H_18_O_7_Na, 393.0950), which suggested a molecular formula of C_20_H_18_O_7_ corresponding to an epoxidized metabolite of **2**. It showed ^1^H- and ^13^C-NMR spectral features closely related to those of **6**. Compared with the NMR spectral data of compound **2**, compound **7** exhibited the absence of olefinic proton signals belonging to the prenyl group. Meanwhile, presence of one oxygen-bearing methine carbon signal at δc 69.2 corresponding with the proton signal at δ_H_ 3.79 and one oxygen-bearing quaternary carbon signal at δc 77.4 were observed based on the HSQC and HMBC correlations. These results suggested that the olefinic double bond of the prenyl group was epoxidized. Thus, the structure of **7** was elucidated as 5,7,3′,4′-tetrahydroxy-5′-(2-epoxy-3-methylbutyl)isoflavone.

### 2.2. Biotransformation of Two Chalcones, Echinatin (**3**) and Isobavachalcone (**4**) with A. niger KCCM 60332

**Figure 2:**
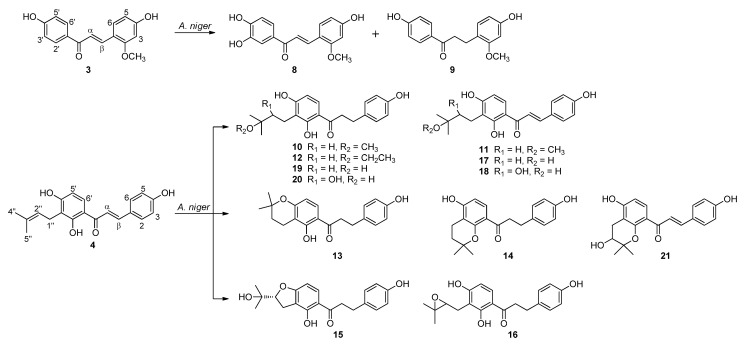
Compounds **8**–**21** obtained by biotransformation of **3** and **4** with *A. niger*.

Biotransformation of echinatin (**3**) furnished two known metabolites **8** and **9**. Biotransformation of isobavachalcone (**4**) afforded twelve metabolites **10**–**21**, of which **10**–**17** and **19** were determined to be structurally new (Figure 2).

Compounds **8** and **9** were obtained as a pale yellow amorphous powder and a yellow amorphous powder, respectively. Their structures were elucidated as (*E*)-1-(3,4-dihydroxyphenyl)-3-(4-hydroxy-2-methoxyphenyl)-prop-2-en-1-one (**8**) and loureirin C (**9**) by comparing their ^1^H-NMR data (Figures S26 and S27) with those in the literatures [17,18].

**Figure 3:**
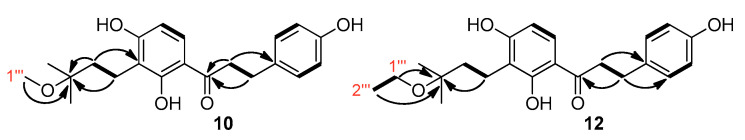
Selected HMBC (^1^H→^13^C) and COSY (^1^H−^1^H) correlations of compounds **10** and **12**.

Compound **10** was acquired as a pale yellow amorphous powder. Its molecular formula of C_21_H_26_O_5_ was established by an [M + Na]^+^ peak at *m*/*z* 381.1678 (calcd C_21_H_26_O_5_Na, 381.1678), which is consistent with 9 degrees of unsaturation. The UV spectrum showed absorption maxima at 220 and 285 nm. The ^13^C-NMR data of **10** showed resonances for twenty-one carbon atoms comprising a typical carbonyl carbon, twelve phenolic carbons, four methylene and three methyl carbons, and one oxygenated quaternary carbon (Table 2). Its ^1^H-NMR data exhibited four aromatic proton signals of ring B constituting an AA’XX’ spin system at δ_H_ 7.04 (2H, d, *J* = 8.6 Hz) and 6.66 (2H, d, *J* = 8.6 Hz); two aromatic proton signals of ring A constituting an AX spin system at δ_H_ 7.63 (1H, d, *J* = 8.9 Hz) and 6.41 (1H, d, *J* = 8.9 Hz); two coupled methylene proton signals at δ_H_ 3.17 (2H, t, *J* = 7.6 Hz) and 2.81 (2H, t, *J* = 7.6 Hz) together with one H-bonded hydroxyl proton signal at δ_H_ 13.13 (2′-OH). Moreover, the ^1^H-NMR data displayed characteristic signals for a 3-hydroxy-3-methylbutyl moiety at δ_H_ 2.50 (2H, m), 1.52 (2H, m), and 1.12 (6H, s), and a three-proton singlet signal for a methoxyl moiety at δ_H_ 3.13 (3H, s). These spectroscopic features of **10** enabled the assignment of the skeleton as 4,2′,4′-trihydroxy-α,β-dihydrochalcone linked with the 3-hydroxy-3-methylbutyl and methoxyl substituents. These inferences were further confirmed by combined analysis of HSQC, HMBC, and COSY spectral data (Figure 3). HMBC correlations were used to confirm the locations of the two substituents. From HMBC correlation of H-2″ (δ_H_ 1.52) to C-3′ (δc 115.2), the 3-hydroxy-3-methylbutyl group was assigned to C-3′. The methoxyl group was deduced to be at C-3″ from the HMBC correlation of H-1′′′ (δ_H_ 3.13) to C-3″ (δc 73.9). According to the above data analysis, compound **10** was elucidated as 4,2′,4′-trihydroxy-3′-(3-*O*-methyl-3-methylbutyl)dihydrochalcone.

Compound **11**, obtained as a yellow amorphous powder, possessed a molecular formula of C_21_H_24_O_5_ as deduced from its HRESIMS peak [M + Na]^+^ at *m*/*z* 379.1519 (calcd C_21_H_24_O_5_Na, 379.1521), which lacks two proton atoms compared with that of **4**. The UV spectrum showed characteristic chalcone absorption maxima at 365 nm. In accordance with the UV spectrum, the H-bonded hydroxyl proton resonance at δ_H_ 14.05 and the carbonyl carbon resonance at δ_C_ 192.6 were consistent with a 2′-hydroxychalcone [19]. The ^1^H- and ^13^C-NMR spectroscopic data for **11** were similar to those of **10** except for new resonances at δ_H_ 7.71 and δc 143.6 and 117.3 (Table 2). In the HSQC spectrum, the resonance at δ_H_ 7.71 showed correlations with the two new resonances at δc 143.6 and 117.3 suggesting the presence of an olefinic double bond. In the HMBC spectrum, the new resonance at δ_H_ 7.71 showed correlations with resonances at δc 191.1 (C=O), 131.0 (C-2,6), 112.0 (C-1′), indicating the presence of an α,β-olefinic group. Thus, the structure of compound **11** was assigned 4,2′,4′-trihydroxy-3′-(3-*O*-methyl-3-methylbutyl)chalcone.

Compound **12** was obtained as a pale yellow amorphous powder. HRESIMS analysis showed the [M + Na]^+^ peak at *m*/*z* 395.1833 which was in accord with the molecular formula C_22_H_28_O_5_. By comparing the NMR data of **12** with those of **10**, it was found that the NMR data of **12** were identical to those of **10** except for the additional ethoxyl proton signals at δ_H_ 3.56 (2H, q, *J* = 7.0 Hz) and 1.29 (3H, t, *J* = 7.0 Hz) (Table 2). It was suggested that the methoxyl group belonging to the 3-methoxy-3-methylbutyl substituent in **10** was replaced by an ethoxyl group in **12**. This deduction was consistent with the difference in molecular ion masses [Δ*m*/*z* = 14.0155 mmu (CH_2_)]. HMBC correlations between the protons at δ_H_ 3.56 (H-1′′′) and 1.29 (H-2′′′) and the carbon at δc 76.3 (C-3′′) confirmed the attachment of the ethoxyl group at C-3′′ (Figure 3). Therefore, compound **12** was assigned 4,2′,4′-trihydroxy-3′-(3-*O*-ethyl-3-methylbutyl)dihydrochalcone.

Compound **13** was obtained as a pale yellow amorphous powder. The HRESIMS of **13** displayed an [M + Na]^+^ peak at *m*/*z* 349.1416 which was consistent with the molecular formula C_20_H_22_O_4_, indicating 10 indices of hydrogen deficiency. UV spectrum showed absorption maxima at 220 and 286 nm. Comparison of its NMR spectroscopic data with those of **10** indicated that **13** have a similar structure but with a 2,2-dimethyldihydropyran ring in the case of **13** (Table 2). On the basis of the HMBC correlation from H-5′ to C-3′′ together with the presence of the intramolecular H-bonded hydroxyl proton signal at δ_H_ 13.17 (2′-OH), it was confirmed that the additional 2,2-dimethyldihydropyran ring was fused to ring A via C-3′ and C-4′ positions. Compound **13** was therefore characterized as 4,2′-dihydroxy-(2,2-dimethyl-3,4-dihydropyran)-(5″,6″:3′,4′)dihydrochalcone.

Compound **14**, isolated as a pale yellow amorphous powder, showed a sodium adduct molecular ion peak at *m*/*z* 349.1415 in the HRESIMS corresponding to the molecular formula C_20_H_22_O_4_, which was the same as that of **13**. The overall NMR data of **14** showed analogous structural features to those of **13** except for the absence of an H-bonded hydroxyl proton resonance in the lower field (Table 3). These data suggested that the 2,2-dimethyldihydropyran ring was fused to C-2′ and C-3′ positions of **14**. Supportive evidence for this deduction was provided by the up-field shifted carbon resonance at δ_C_ 155.2 (C-2′) after combined analysis of its HSQC and HMBC data. Moreover, the NMR data of **6** were in good accordance with those of deoxydihydroxanthoangelol H in which a methoxyl group was attached at C-4′ instead of a hydroxyl group in **6** [20]. Thus, the structure of **14** was assigned 4,4′-dihydroxy-(2,2-dimethyl-3,4-dihydropyran)-(5″,6″:3′,2′)dihydrochalcone.

Compound **15**, an optically active compound (${\left[\alpha \right]}_{D}^{20}$ −52.2), was obtained as a pale yellow amorphous powder. Its molecular formula was defined as C_20_H_22_O_5_ by the HRESIMS peak [M + Na]^+^ at *m*/*z* 365.1364 (calcd C_20_H_22_O_5_Na, 365.1365). The ^1^H- and ^13^C-NMR spectroscopic data (Table 3) in conjunction with HSQC and HMBC experiments delineated the presence of twenty carbon atoms consisting of the following functional groups: two methyl, three methylene, seven methine, and seven quaternary and a carbonyl carbons (Table 3). In the ^1^H-NMR spectrum, resemblance of the resonance signals between compounds **15** and **10** suggested that both have the same skeleton of α,β-dihydrochalcone. Meanwhile, the ^1^H-NMR spectrum of **15** obviously showed characteristic signals for a 2-(1-methyl-1-hydroxyethyl)dihydrofuran ring fused to an aromatic ring at δ_H_ 3.04 (t, *J* = 8.8 Hz, 2H, H-1″), 4.71 (d, *J* = 8.8 Hz, 1H, H-2″), 1.13 (s, 3H, H-4″), and 1.12 (s, 3H, H-5″) [21]. Analysis of the HMBC correlations revealed that the ring was fused at C-3′ and C-4′ positions in ring A by the correlations from the H-1″ (δ_H_ 3.04) and H-2″ (δ_H_ 4.71) to C-3′ (δ_C_ 113.3) and C-4′ (δ_C_ 166.8). The absolute configuration at C-2′′ was proposed as *R* by comparison of the specific rotation of **15** with those of coryaurone A (${\left[\alpha \right]}_{D}^{25}$ −44.9) [22], artonitidin A (${\left[\alpha \right]}_{D}^{20}$ −25.7) [23], and anodendroic acid (${\left[\alpha \right]}_{D}^{25}$ +42.0) [24]. Taken together, compound **15** was elucidated to be (2″*R*)-4,2′-dihydroxy-[2-(1-hydroxy-1-methyl)- 2,3-dihydrofuran]-(4″,5″:3′,4′)dihydrochalcone.

Compound **16** was isolated as a pale yellow amorphous powder. HRESIMS indicated a molecular formula of C_20_H_22_O_5_, according to its sodium adduct ion peak at *m*/*z* 365.1366 with 10 indices of hydrogen deficiency. Analysis of the ^1^H- and ^13^C-NMR data of **16** exhibited signal patterns closely resembling to those of **10**, indicating that compound **16** has a skeleton of 4,2′,4′-trihydroxydihydrochalcone. Whereas, the substituent attached to C-3′ position was found to be different, as the ^1^H-NMR data of **16** revealed signals for a 2,3-epoxy-3-methylbutyl group instead of a 3-methoxy-3-methylbutyl group in **10**. The attachment of the 2,3-epoxy-3-methylbutyl group was further confirmed to be at C-3′ on the basis of the HMBC correlations from H-1′′ (δ_H_ 2.78 and 2.46) and H-2′′ (δ_H_ 3.67) to C-3′ (δ_C_ 107.8). Accordingly, compound **16** was elucidated as 4,2′,4′-trihydroxy-3′-(2,3-epoxy-3-methylbutyl)dihydrochalcone.

Compound **17**, obtained as a yellow amorphous powder, had a molecular formula of C_20_H_22_O_5_ according to its sodium adduct ion peak at *m/z* 365.1365 ([M + Na]^+^, calcd for C_20_H_22_O_5_Na, 365.1365) with 10 degrees of unsaturation. The UV absorption of **17** displayed absorption maxima at 370 nm typical of a chalcone. The ^1^H- and ^13^C-NMR spectra of **17** were remarkably similar to those of **11**, except for the resonances for a methyl group at C-1′′′ position (Table 2 and Table 3). The HMBC correlations from H-1′′ (δ_H_ 2.57) and H-2′′ (δ_H_ 1.50) to C-3′ (δ_C_ 115.8) confirmed the 3-hydroxy-3-methylbutyl group to be attached at C-3′ on the skeleton of 4,2′,4′-trihydroxychalcone. Thus, compound **17** was characterized as 4,2′,4′-trihydroxy-3′-(3-hydroxy-3-methylbutyl)chalcone.

Compound **19** was obtained as a pale yellow amorphous powder. Its molecular formula was established as C_20_H_24_O_5_ by its HRESIMS data ([M + Na]^+^, calcd for C_20_H_24_O_5_Na, 367.1521). Comparison of the ^1^H- and ^13^C-NMR data of **19** and **10** revealed that the resonance signals for the methyl group at C-1′′′ of **10** were absent in **19**, suggesting the isoprene unit at C-3′ of **19** was a 3-hydroxy-3-methylbutyl moiety (Table 2 and Table 3). The connectivity of 3-hydroxy-3-methylbutyl moiety at C-3′ was further secured by the HMBC correlations from H-1′′ (δ_H_ 2.54) and H-2′′ (δ_H_ 1.47) to C-3′ (δ_C_ 115.6). Compound **19** was therefore identified as 4,2′,4′-trihydroxy-3′-(3-hydroxy-3-methylbutyl)dihyrochalcone.

Structures of three other known compounds were identified as brosimacutin M (**18**) [25], brosimacutin H (**20**) [26], and bavachromanol (**21**) [27,28] by comparing their spectral data with those reported in the literatures (Figures S74–S76). However, absolute configuration of their hydroxyl groups remained undetermined due to the limited quantities of the isolates. Further study may be necessary to determine the absolute configuration in compounds **18**, **20**, and **21**.

### 2.3. Proposed Metabolic Pathways of Isobavachalcone (**4**) Catalyzed by A. niger KCCM 60332

**Figure 4:**
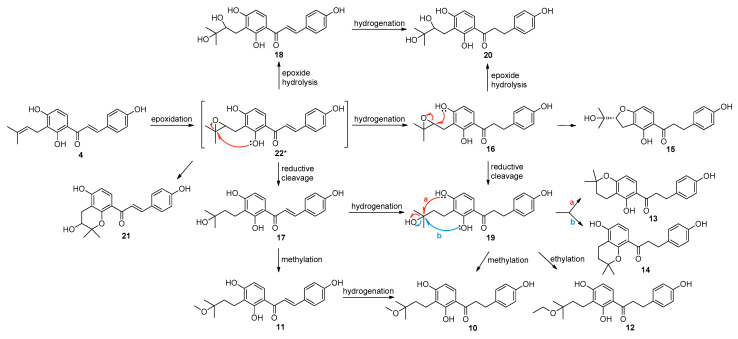
Proposed metabolic pathways of **4** catalyzed by *A. niger*. Pathways a and b, represented by the arrows in red and blue respectively, are proposed as the two routes to form a new ring in compound **19**. * Compound **22** is proposed as an intermediate which could not be unambiguously identified in this study.

Biotransformation of isobavachalcone (**4**) by the selected fungal strain *A. niger* produced metabolites **10**–**21** through hydrogenation, epoxidation, hydrolysis, reduction, cyclization, and alkylation (Figure 4). The prenyl substituent and α,β-double bond were the major sites for biotransformation by *A. niger*.

Regarding the metabolic relationships of these metabolites, **22** was proposed as a potential intermediate which could not be unambiguously identified in this study. The proposed intermediate **22** could be rationalized by initial epoxidation of the prenyl group at C-3′′. Further reductive cleavage or hydrolysis of the epoxide intermediate led to the generation of metabolites **17** or **18**, respectively. Moreover, *O*-methylation of the hydroxyl group at C-3′′ in **17** could form **11**. Meanwhile, a spontaneous intramolecular attack of the neighboring oxygen atom at C-2′ in **22** could lead to the metabolite **21**. Hydrogenation of the α,β-double bonds in **22**, **17**, **18,** and **11** could produce their corresponding hydrogenated metabolites **16**, **19**, **20**, and **10**, respectively. Metabolites **16** and **19** could be considered as intermediates to produce the rest of the metabolites. In the case of **16**, a spontaneous intramolecular attack of the neighboring oxygen atom at C-4′ could result in the opening of epoxide ring to form its respective metabolite **15**. In the case of **19**, similarly, intramolecular cyclization of the prenyl group by the neighboring hydroxyl group at C-2′ or C-4′ could form a 2,2-dimethyldihydropyran moiety in **14** (pathway b) or **13** (pathway a). Additionally, metabolite **12** could be formed by *O*-ethylation of the hydroxyl group at C-3′′ of **19**.

### 2.4. Cytotoxicity Evaluation

The parent compounds **1**–**4** and all isolated metabolites **5**–**21** were evaluated for in vitro cytotoxic potential against human cancer cell lines A375P, HT-29, and MCF-7 using modified MTT method [29]. The results are presented on Table 4. Noteworthily, compounds **1** and **12** showed the strongest cytotoxic activities against human cancer cell lines A375P, A549, and MCF-7 with IC_50_ values ranging from 4.4 to 10.1 μM, while compounds **2** and **4** were moderately cytotoxic.

## 3. Materials and Methods

### 3.1. General Experimental Procedures

Optical rotations were recorded with a 343 Plus polarimeter (Perkin Elmer, Waltham, MA, USA). UV spectra were recorded on a V-530 spectrophotometer (JASCO, Tokyo, Japan). IR spectra were obtained on a Frontier FT-IR/NIR spectrometer (PerkinElmer, Waltham, MA, USA). CD spectra were recorded on a JASCO J-815 CD spectrometer (JASCO, Tokyo, Japan). NMR experiments were recorded using an Avance III 400 spectrometer (Bruker, Fällanden, Switzerland) and Varian Unity INOVA 500 and 600 spectrometers (Varian, Palo Alto, CA, USA) with TMS as the internal standard. HRESIMS were determined on Waters Synapt G2 QTOF (Waters, Manchester, UK). TLC was carried out on Merck silica gel F_254_-precoated glass plates. Chromatography was performed on a Waters 1525 Binary HPLC pump connected to a 996 Photodiode Array (PDA) detector using Isco Allsphere ODS-2 (10 μm, 10 × 250 mm) and Zorbax SB-C8 (5 μm, 4.6 × 150 mm) columns with methanol (solvent A) and water (solvent B).

4′-Dihydroxyacetophenone, 2,4-dihydroxybenzaldehyde, 2′,4′-dihydroxyacetophenone, and 4-hydroxybenzaldehyde were purchased from Tokyo Chemical Industry Co., Ltd. Demethylzeylasteral (DZ) used as a reference standard in the bioassays was purchased from Biopurify Phytochemicals, Ltd. All the ingredients for microbial media including D-glucose, peptone, malt extract, yeast extract, and potato dextrose medium were purchased from Becton, Dickinson and Co.

### 3.2. Plant Material

The roots and rhizomes of licorice (*Glycyrrhiza inflata*) were purchased from the herbal market Sehwadang (Gwangju, Korea) in March 2018, which were identified by Dae Hyo Pharmacy Co., Ltd. (Suwon, Korea).

### 3.3. Extraction and Isolation of Substrates **1** and **2**

The dried plant material (2.5 kg) was powdered and extracted with 95% EtOH (7.5 L × 3) and was dispersed in water and successively extracted with *n*-hexane, CH_2_Cl_2_, EtOAc and *n*-BuOH. The CH_2_Cl_2_ extract (50 g) was separated by column chromatography eluted with CHCl_3_: MeOH to obtain fractions C1–C20. Fraction C11 was further separated by HPLC to yield compound **1** (30 mg), and fraction C15 was further separated by HPLC to yield compound **2** (45 mg). The structures of **1** and **2** were confirmed by comparison of their ^1^H-NMR data with those previously reported [15,30].

Licoisoflavanone (**1**): ^1^H NMR (CD_3_OD, 400 MHz, δ in ppm, *J* in Hz) δ 6.87 (1H, d, *J* = 8.3, H-6′), 6.66 (1H, d, *J* = 10.0, H-1″), 6.32 (1H, d, *J* = 8.3, H-5′), 5.89 (1H, d, *J* = 2.2, H-6), 5.88 (1H, d, *J* = 2.2, H-8), 5.66 (1H, d, *J* = 10.0, H-2″), 4.58 (1H, t, *J* = 10.2, H-2a), 4.44 (1H, dd, *J* = 10.2, 5.4, H-2b), 4.20 (1H, dd, *J* = 10.2, 5.4, H-3), 1.38 (6H, s, H-4″,5″).

Glycyrrhisoflavone (**2**): ^1^H NMR (CD_3_OD, 400 MHz, δ in ppm, *J* in Hz) δ 7.96 (1H, s, H-2), 6.87 (1H, s, H-6′), 6.71 (1H, s, H-2′), 6.30 (1H, s, H-8), 6.19 (1H, s, H-6), 5.34 (1H, m, H-2″), 3.35 (2H, d, *J* = 7.3, H-1″), 1.72 (6H, s, H-4″,5″).

### 3.4. Synthesis of Substrates **3** and **4**

Echinatin (**3**) and isobavachalcone (**4**) were synthesized for biotransformation due to their low yield from natural sources. Briefly, echinatin (**3**) was synthesized through acid-mediated Claisen-Schmidt condensation using 2,4-dihydroxybenzaldehyde with 4′-hydroxyacetophenone as starting materials (Scheme S1) [31]. Isobavachalcone (**4**) was synthesized through Claisen-Schmidt condensation using 4-hydroxybenzaldehyde with 2′,4′-dihydroxyacetophenone as starting materials (Scheme S2) [32]. Structures of the final products **3** and **4** were confirmed by comparing their spectroscopic data with those reported in the literatures [33,34].

Echinatin (**3**): ^1^H-NMR (CD_3_OD, 400 MHz, δ in ppm, *J* in Hz) δ 8.03 (1H, d, *J* = 15.6, H-α), 7.97 (2H, d, *J* = 8.8, H-2′,6′), 7.62 (1H, d, *J* = 15.6, H-α), 7.61 (1H, d, *J* = 8.5, H-6), 6.89 (2H, d, *J* = 8.8, H-3′,5′), 6.47 (1H, d, *J* = 2.2, H-3), 6.44 (1H, dd, *J* = 8.5, 2.2, H-5), 3.89 (3H, s, 2-OMe).

Isobavachalcone (**4**): ^1^H-NMR (CD_3_OD, 400 MHz, δ in ppm, *J* in Hz) δ 7.84 (1H, d, *J* = 8.9, H-6′), 7.78 (1H, s, *J* = 15.4, H-β), 7.64 (1H, d, *J* = 15.4, H-α), 7.62 (2H, d, *J* = 8.6, H-2,6), 6.85 (2H, d, *J*= 8.6, H-3,5), 6.43 (1H, d, *J* = 8.9, H-5′), 5.23 (1H, m, H-2′′), 3.33 (2H, overlapped, H-1′′), 1.78 (3H, s, H-4′′), 1.66 (3H, s, H-5′′).

### 3.5. Microorganisms and Screening for Biostransformation

All the microorganisms were obtained from the Korean Collection for Type Cultures (KCTC, Daejeon, Korea) and Korean Culture Center of Microorganisms (KCCM, Seoul, Korea). The strains used for preliminary screening are as follows: *Absidia coerulea* KCTC 6936, *Aspergillus niger* KCCM 60332, *Aspergillus oryzae* KCCM 60345, *Hormoconis resinae* KCTC 6966, *Mortierella ramanniana* var. *angulispora* KCTC 6137, *Penicillium chrysogenum* KCTC 6933, *Pichia pastoris* KCTC 7190, *Tremella mesenterica* KCTC 7131.

Fermentation experiments were performed in three types of media. *A. coerulea*, *A. niger*, *A. oryzae*, *P. chrysogenum* were incubated on malt medium (malt extract 20 g/L, D-glucose 20 g/L, peptone 1 g/L). *H. resinae*, *M. ramanniana* var. *angulispora*, *P. pastoris* were cultured on potato sucrose medium (potato dextrose 24 g/L and sucrose 20 g/L). *T. mesenterica* was cultured on yeast-malt medium (D-glucose 10 g/L, peptone 5 g/L, malt extract 3 g/L, and yeast extract 3 g/L).

Biotransformation was carried out according to the two-stage procedure [35]. In the screening studies, the actively growing microbial cultures were incubated in 250 mL flasks containing 50 mL of media with gentle agitation (200 rpm) at 25 °C in a temperature-controlled shaking incubator. Ethanol solution (20 mg/mL, 50 μL) of the substrate **1**, **2**, **3**, or **4** was added to each flask 24 h after inoculation. And further incubation was performed under the same condition for six days. Two controls were used for biotransformation in this study, i.e., culture controls consisting of microorganisms growing in the culture media without substrates, and substrate controls consisting of culture media and substrates incubated without microorganisms. General sampling and TLC monitoring were performed on Merck silica gel F_254_-precoated glass plates. *A. niger* was identified as the most potent strain to metabolize **1**–**4** and therefore selected for scale-up fermentation.

### 3.6. Scale-up Fermentation, Extraction, and Isolation of Metabolites **5**–**21**

For scale-up fermentation, *A. niger* was incubated in 500 mL Erlenmeyer flasks containing 150 mL of media. After a further 24 h incubation, the ethanol solution (20 mg/mL, 150 μL) of each substrate (**1**, **2**, **3**, or **4**) was evenly distributed to each flask containing stage II cultures (Table 5).

After incubation, the liquid cultures of **1**, **2**, **3**, or **4** were extracted three times with equal volumes of EtOAc, and the organic layer was collected and concentrated at reduced pressure.

The organic extract of **1** incubated with *A. niger* was subject to HPLC with a gradient solvent system of 45% MeOH to 67% MeOH to afford **5** (4.5 mg, t_R_ = 50.3 min) at a flow rate of 2.0 mL/min. 

The organic extract of **2** incubated with *A. niger* was subject to HPLC with a gradient solvent system of 55% MeOH to 69% MeOH to afford **6** (3.2 mg, t_R_ = 14.2 min) and **7** (2.0 mg, t_R_ = 16.2 min) at a flow rate of 2.0 mL/min. 

The organic extract of **3** incubated with *A. niger* was subject to HPLC with a gradient solvent system of 45% MeOH to 72% MeOH to afford **8** (5.0 mg, t_R_ = 27.1 min), **9** (2.5 mg, t_R_ = 31.2 min).

The organic extract of **4** incubated with *A. niger* was subject to HPLC with a gradient solvent system of 50% MeOH to 90% MeOH to furnish nine fractions (Fr. A-I) and **13** (1.9 mg, t_R_ = 50.3 min) at a flow rate of 2.0 mL/min. Fr. G, H, or I was further separated on HPLC eluting with a gradient solvent system from 60% MeOH to 84% MeOH to yield **10** (1.7 mg, t_R_ = 19.3 min), **11** (2.8 mg, t_R_ = 14.6 min), and **12** (3.6 mg, t_R_ = 16.6 min), respectively. Fr. D was purified by HPLC with an isocratic solvent system of 65% MeOH to yield **14** (3.3 mg, t_R_ = 20.0 min). Fr. E was further purified by HPLC with an isocratic solvent system of 65% MeOH to yield **15** (3.6 mg, t_R_ = 18.6 min) and **16** (3.7 mg, t_R_ = 19.4 min). Fr. F was further purified on HPLC eluting with an isocratic solvent system of 58% MeOH to yield **17** (2.8 mg, t_R_ = 16.5 min). Fr. C was subject to HPLC with a gradient solvent system from 54% MeOH to 60% MeOH to yield **18** (2.2 mg, t_R_ = 16.7 min) and **19** (2.7 mg, t_R_ = 20.1 min). Fr. A was purified by HPLC with an isocratic solvent system of 55% MeOH to yield **20** (2.1 mg, t_R_ = 7.9 min). Fr. B was purified by HPLC with an isocratic solvent system of 62% MeOH to yield **21** (1.9 mg, t_R_ = 14.0 min).

### 3.7. Spectroscopic Data of Metabolites **5**–**21**

#### 3.7.1. Spectroscopic Data of the New Compounds **5**–**7**, **10**–**17**, and **19**

1. Compound **5**

Yellow amorphous powder; ${\left[\alpha \right]}_{D}^{20}$ +3.1 (c 0.10, MeOH); UV (MeOH) λ_max_ (log ε): 293 (1.25) nm; IR ν_max_: 3438, 2991, 2865, 1650, 1514, 1094 cm^−1^; HRESIMS *m/z*: 393.0953 [M + Na]^+^ (calcd for C_20_H_18_O_7_Na, 393.0950); ^1^H- and ^13^C-NMR data (see Table 1).

2. Compound **6**

Yellow amorphous powder; ${\left[\alpha \right]}_{D}^{20}$ −8.3 (c 0.10, MeOH); UV (MeOH) λ_max_ (log ε): 262 (1.08) nm; IR ν_max_: 3321, 2931, 2607, 1741, 1522, 1098 cm^−1^; HRESIMS *m/z*: 411.1060 [M + Na]^+^ (calcd for C_20_H_20_O_8_Na, 411.1056); ^1^H- and ^13^C-NMR data (see Table 1).

3. Compound **7**

Yellow amorphous powder; ${\left[\alpha \right]}_{D}^{20}$ +5.5 (c 0.10, MeOH); UV (MeOH) λ_max_ (log ε): 262 (0.90) nm; IR ν_max_: 3431, 2922, 2858, 1651, 1512, 1187, 1078 cm^−1^; HRESIMS m/z: 393.0951 [M + Na]^+^ (calcd for C_20_H_18_O_7_Na, 393.0950); ^1^H- and ^13^C-NMR data (see Table 1).

4. Compound **10**

Pale yellow amorphous powder; UV (MeOH) λ_max_ (log ε) 220 (1.25), 285 (0.98) nm; IR *ν*_max_ 3278, 2938, 1614, 1515, 1438, 1225, 1098, 812 cm^−1^; HRESIMS *m/z* 381.1678 [M + Na]^+^ (calcd for C_21_H_26_O_5_Na, 381.1678; ^1^H- and ^13^C-NMR data (see Table 2).

5. Compound **11**

Yellow amorphous powder; UV (MeOH) λ_max_ (log ε) 365 (1.38) nm; IR *ν*_max_ 3322, 2947, 1635, 1450, 1236, 1109, 1017 cm^−1^; HRESIMS *m/z* 379.1519 [M + Na]^+^ (calcd for C_21_H_24_O_5_Na, 379.1521); ^1^H- and ^13^C-NMR data (see Table 2).

6. Compound **12**

Pale yellow amorphous powder; UV (MeOH) λ_max_ (log ε) 220 (1.32), 286 (0.90), 326 (sh) nm; IR *ν*_max_ 3365, 2933, 1618, 1516, 1437, 1226, 805 cm^−1^; HRESIMS *m/z* 395.1833 [M + Na]^+^ (calcd for C_22_H_28_O_5_Na, 395.1834); ^1^H- and ^13^C-NMR data (see Table 2).

7. Compound **13**

Pale yellow amorphous powder; UV (MeOH) λ_max_ (log ε) 220 (1.83), 286 (1.41), 327 (sh) nm; IR *ν*_max_ 3393, 2925, 1598, 1516, 1418, 1372, 1218, 1110 cm^−1^; HRESIMS *m/z* 349.1416 [M + Na]^+^ (calcd for C_20_H_22_O_4_Na, 349.1416; ^1^H- and ^13^C-NMR data (see Table 2).

8. Compound **14**

Pale yellow amorphous powder; UV (MeOH) λ_max_ (log ε) 222 (1.31), 280 (0.86), 313 (sh) nm; IR *ν*_max_ 3300, 2922, 1584, 1515, 1440, 1371, 1218, 1158, 1050 cm^−1^; HRESIMS *m/z* 349.1415 [M + Na]^+^ (calcd for C_20_H_22_O_4_Na, 349.1416); ^1^H- and ^13^C-NMR data (see Table 3).

9. Compound **15**

Pale yellow amorphous powder; ${\left[\alpha \right]}_{D}^{25}$ −52.2 (*c* 0.5, MeOH); UV (MeOH) λ_max_ (log ε) 214 (1.44), 294 (0.87) nm; IR *ν*_max_ 3288, 2976, 1614, 1516, 1439, 1370, 1220, 1096, 1054 cm^−1^; HRESIMS *m/z* 365.1364 [M + Na]^+^ (calcd for C_20_H_22_O_5_Na, 365.1365); ^1^H- and ^13^C-NMR data (see Table 3).

10. Compound **16**

Pale yellow amorphous powder; ${\left[\alpha \right]}_{D}^{25}$ −11.0 (*c* 0.8, MeOH); UV (MeOH) λ_max_ (log ε) 215 (1.78), 286 (1.14), 324 (sh) nm; IR *ν*_max_ 3358, 2933, 1616, 1516, 1371, 1218, 1108, 803 cm^−1^; HRESIMS *m/z* 365.1366 [M + Na]^+^ (calcd for C_20_H_22_O_5_Na, 365.1365); ^1^H- and ^13^C-NMR data (see Table 3).

11. Compound **17**

Yellow amorphous powder; UV (MeOH) λ_max_ (log ε) 370 (1.13) nm; IR *ν*_max_ 3358, 2933, 1616, 1516, 1371, 1218, 1108, 803 cm^−1^; HRESIMS *m/z* 365.1365 [M + Na]^+^ (calcd for C_20_H_22_O_5_Na, 365.1365); ^1^H- and ^13^C-NMR data (see Table 3).

12. Compound **19**

Yellow amorphous powder; UV (MeOH) λ_max_ (log ε) 220 (1.32), 286 (1.02), 324 (sh) nm; IR *ν*_max_ 3269, 2971, 1615, 1516, 1442, 1370, 1228, 1099, 1051 cm^−1^; HRESIMS *m/z* 367.1521 [M + Na]^+^ (calcd for C_20_H_24_O_5_Na, 367.1521); ^1^H- and ^13^C-NMR data (see Table 3).

#### 3.7.2. ^1^H-NMR Data of the Compounds **8**, **9**, **18**, **20**, and **21**

1. Compound **8**

^1^H-NMR (CD_3_OD, 400 MHz, δ in ppm, *J* in Hz) δ 8.00 (1H, d, *J* = 15.7, H-α), 7.60 (1H, d, *J* = 15.7, H-β), 7.59 (1H, d, *J* = 8.4, H-6), 7.52 (1H, dd, *J* = 8.2, 2.0, H-6′), 7.49 (1H, d, *J* = 2.0, H-2′), 6.87 (1H, d, *J* = 8.2, H-5′), 6.48 (1H, d, *J* = 2.1, H-3), 6.46 (1H, dd, *J* = 8.4, 2.1, H-5), 3.89 (3H, s, OMe).

2. Compound **9**

^1^H-NMR (CD_3_OD, 400 MHz, δ in ppm, *J* in Hz) δ 7.87 (2H, d, *J* = 8.8, H-2′,6′), 6.93 (1H, d, *J* = 8.2, H-6), 6.83 (2H, d, *J* = 8.8, H-3′,5′), 6.39 (1H, d, *J* = 2.2, H-3), 6.29 (1H, d, *J* = 8.2, 2.2, H-5), 3.77 (3H, s, OMe), 3.10 (2H, d, *J* = 7.4, H-α), 2.85 (2H, t, *J* = 7.4, H-β).

3. Compound **18**

^1^H-NMR (DMSO-*d*_6_, 400 MHz, δ in ppm, *J* in Hz) δ 14.28 (OH), 7.94 (1H, d, *J* = 9.1, H-6′), 7.74~7.67 (4H, overlapped, H-α,β,2,6), 6.84 (2H, d, *J* = 8.5, H-3,5), 6.31 (1H, d, *J* = 9.1, H-5′), 3.40 (1H, dd, *J* = 9.7, 2.1, H-2″), 2.91 (1H, dd, *J* = 13.5, 2.1, H-1″a), 2.47 (1H, overlapped, H-1″b), 1.11 (3H, s, H-4″), 1.10 (3H, s, H-5″).

4. Compound **20**

^1^H-NMR (DMSO-*d*_6_, 400 MHz, δ in ppm, *J* in Hz) δ 13.27 (OH), 7.62 (1H, d, *J* = 9.1, H-6′), 7.06 (2H, d, *J* = 7.9, H-2,6), 6.67 (2H, d, *J* = 7.9, H-3,5), 6.30 (1H, d, *J* = 9.1, H-5′), 3.39 (1H, overlapped, H-2″), 3.15 (2H, t, *J* = 7.5, H-α), 2.86 (1H, m, H-1″a), 2.81 (2H, t, *J* = 7.5, H-β), 2.47 (1H, overlapped, H-1″b), 1.09 (6H, s, H-4″,5″).

5. Compound **21**

^1^H-NMR (DMSO-*d*_6_, 400 MHz, δ in ppm, *J* in Hz) δ 7.50 (2H, d, *J* = 8.9, H-2,6), 7.48 (1H, d, *J* = 15.8, H-β), 7.42 (1H, d, *J* = 15.8, H-α), 7.39 (1H, d, *J* = 8.6, H-6′), 6.82 (2H, d, *J* = 8.6, H-3,5), 6.44 (1H, d, *J* = 8.6, H-5′), 3.68 (1H, m, H-2″), 2.80 (1H, dd, *J* = 17.2, 5.4, H-1″a), 2.43 (1H, dd, *J* = 17.2, 7.5, H-1″b), 1.33 (3H, s, H-4″), 1.24 (3H, s, H-5″).

### 3.8. Cytotoxicity Assay

Tested compound solutions were prepared in DMSO and stored as stock solution at 4 °C. Upon dilution into culture medium, the final DMSO concentration was below 1% (*v*/*v*), a concentration without effect on cell replication. The human cancer cell lines consisted of human melanoma (A375P), human colorectal adenocarcinoma (HT-29), and breast adenocarcinoma (MCF-7). All cell lines were cultured in Dulbecco’s modified Eagle’s medium (DMEM) with 5% fetal bovine serum (FBS), 100 U/mL penicillin and 100 μg/mL streptomycin in a humidified incubator at 37 °C with 5% CO_2_. The cells were plated into 96-well plates at approximately 5000 cells per well suspended in 100 μL medium. After being cultivated for 24 h, the culture medium was removed, and serial dilutions of the test compounds were treated into each well containing cells in duplicates. After being cultivated for 48 h, the culture medium was removed and 100 μL of MTT solution (0.5 mg/mL) was added to each well and incubated for another 4 h. Following dissolving the MTT formazan crystals, absorbance of the plates was read on a microplate reader at 490 nm for measuring the reduction of the tetrazolium salt MTT (3-(4,5-dimethylthiazol-2-yl)-2,5-diphenyltetrazolium bromide) by metabolically active cells. Demethylzeylasteral (DZ) was used as a positive control. IC_50_ values were calculated and are presented in the Table 4.

## 4. Conclusions

Biotransformation of licoisoflavanone (**1**), glycyrrhisoflavone (**2**), echinatin (**3**), and isobavachalcone (**4**) by the filamentous fungus *A. niger* furnished twelve new (**5**–**7**, **10**–**17** and **19**) and five known (**8**, **9**, **18**, **20** and **21**) metabolites. Compounds **1** and **12** showed most considerable cytotoxic activities against all human cancer cell lines investigated including A375P, MCF-7, and HT-29.

*A. niger* is a filamentous ascomycete fungus that is ubiquitous in soils, plants, animals, and even in marine environments [36]. Investigations focused on microbial biotransformation of bioactive compounds revealed that *A. niger* has been considered as a potential biocatalyst for the modification of chemicals to identify undescribed derivatives or chemical intermediates [37,38]. In this study, *A. niger* demonstrated its ability to catalyze various reactions for isoflavonoids and chalcones including hydroxylation, hydrogenation, epoxidation, hydrolysis, reduction, cyclization, and alkylation reactions. It’s worth noting that the metabolic routes were affected by the presence or absence of a linear prenyl group in the substrates. In the presence of a linear prenyl group in substrates **2** and **4**, metabolism preferentially took place on the prenyl group by *A. niger*. Conversely, metabolism took place on ring A or α,β-double bond in substrates **1** and **3** which lack linear prenyl groups. It is hypothesized that presence of the linear prenyl group may be given a higher priority in the regioselectivity rendered by *A. niger*.

In traditional herbal medicine and oriental clinical practice, licorice has been used as a potential anti-cancer or cancer chemopreventive natural agent [39]. Biological investigations have revealed that licorice extracts show different cytotoxic activities [40,41,42,43]. However, most studies on the effective constituents responsible for these bioactivities are focused on the major compounds such as glycyrrhizin, isoangustone A, glabridin, liquiritigenin, isoliquiritigenin, and licochalcone A [44,45,46,47]. Little is known on the biological effects of the phenolic compounds that have been isolated from licorice. In this study, comparative evaluation on the cytotoxicity of the licorice constituents (**1**–**4**) and their metabolites (**5**–**21**) has been conducted to investigate structure-cytotoxic activity relationship using three human cancer cell lines A375P, HT-29 and MCF-7. Compound **1** showed potent cytotoxic activities, with IC_50_ values ranging from 7.5 to 9.2 μM against the three cancer cell lines tested. However, its metabolite **5** was inactive, indicating that introduction of the hydroxyl group at C-8 of licoisoflavanone could decrease its cytotoxic activity. Meanwhile, compound **2** showed moderate cytotoxic activity whereas its metabolites **6** and **7** were inactive, suggesting that the prenyl group at C-5′ position could improve the cytotoxic activities instead of the 2,3-dihydroxy-3-methylbutyl or 2,3-epoxy-3-methylbutyl groups. On the other hand, metabolite **8** showed improved cytotoxic activities compared with its parent compound **3**, indicating the importance of the hydroxyl group at C-3′ position for retrochalcone. Noteworthily, metabolite **12** showed more potent cytotoxic activities than its parent compound **4** against A375P, HT-29 and MCF-7 cancer cell lines with IC_50_ values ranging from 4.4 to 10.1 μM. Whereas other metabolites (**10**, **11**, and **13**–**21**) exhibited reduced cytotoxic activities compared with **4** against the three cell lines tested.

These results generate new ideas for the investigation of cytotoxic constituents from licorice and provide a potential value for the development of more potent inhibitors of tumor promotion.

## Figures and Tables

**Table 1:** ^1^H- and ^13^C-NMR data for **5**–**7** in CD_3_OD.

No.	5		6		7	
C/H	δ_H_^a^ (*J*/Hz)	δ_C_ ^b^	δ_H_ ^a^ (*J*/Hz)	δ_C_ ^b^	δ_H_^a^ (*J*/Hz)	δ_C_ ^b^
2	4.65 t (8.3)	70.5	8.04 s	153.5	8.03 s	153.6
3	4.53 dd (10.0, 5.4) 4.23 dd (10.0, 5.4)	46.9		127.2		123.3
4		197.9		180.9		180.8
5		156.0		162.4		162.4
6	5.99 s	95.4	6.22 d (2.1)	98.7	6.22 d (2.0)	98.8
7		157.0		164.6		164.5
8		125.3	6.34 d (2.1)	93.4	6.33 d (2.0)	93.5
9		149.2		158.3		158.3
10		102.0		104.9		104.9
1′		115.7		123.6		122.7
2′		150.7	6.81 d (2.1)	122.3	6.75 d (2.1)	120.8
3′		110.9		143.9		141.2
4′		153.3		145.1		145.3
5′	6.34 d (8.3)	108.5		122.0		120.6
6′	6.91 d (8.3)	129.3	6.92 d (2.1)	114.2	6.86 s	113.8
1″	6.69 d (10.0)	116.6	2.97 dd (14.0, 1.9) 2.68 dd (14.0, 10.2)	32.8	3.03 dd (16.7, 5.4) 2.74 dd (16.7, 7.3)	30.7
2″	5.68 d (10.0)	129.1	3.65 dd (10.2, 1.9)	79.2	3.79 m	69.2
3″		75.1		72.5		77.4
4″	1.40 s	26.5	1.25 s	24.3	1.39 s	24.4
5″	1.40 s	26.4	1.25 s	23.6	1.31 s	19.6

Assignments were achieved based on HSQC and HMBC. *J* values (Hz) are given in parentheses. ^a^ 400 MHz. ^b^ 100 MHz.

**Table 2:** ^1^H- and ^13^C-NMR data for **10**–**13**.

No.	10 ^a^		11 ^a^		12 ^b^		13 ^b^	
C/H	δ_H_^c^ (*J*/Hz)	δ_C_ ^d^	δ_H_ ^c^ (*J*/Hz)	δ_C_ ^d^	δ_H_^c^ (*J*/Hz)	δ_C_ ^d^	δ_H_^c^ (*J*/Hz)	δ_C_ ^d^
α	3.17 t (7.6)	38.9	7.71 s	117.3	3.18 t (7.4)	40.0	3.17 t (7.4)	39.9
β	2.81 t (7.6)	29.3	7.71 s	143.6	2.97 t (7.4)	29.9	2.97 t (7.4)	29.9
C=O		203.9		191.1		204.0		203.9
1		131.0		125.6		133.2		133.3
2,6	7.04 d (8.6)	129.2	7.73 d (8.6)	131.0	7.10 d (8.2)	129.6	7.11 d (8.3)	129.6
3,5	6.66 d (8.6)	115.0	6.84 d (8.6)	115.9	6.77 d (8.2)	115.5	6.77 d (8.3)	115.5
4		155.5		160.5		154.2		154.1
1′		111.5		112.0		112.8		112.3
2′		162.3		163.7		162.3		163.0
3′		115.2		115.4		116.8		109.3
4′	13.13 s (OH)	162.3	14.05 s (OH)	163.7	13.10 s (OH)	162.6	13.17 s (OH)	160.8
5′	6.41 d (8.9)	107.8	6.47 d (8.9)	107.9	6.41 d (8.9)	108.7	6.32 d (9.0)	109.3
6′	7.63 d (8.9)	129.8	7.97 d (8.9)	129.5	7.53 d (8.9)	129.6	7.51 d (9.0)	128.9
1″	2.50 (overlay)	16.6	2.53 m	16.7	2.74 t (6.8)	15.5	2.69 t (6.8)	16.4
2″	1.52 m	37.4	1.55 m	37.4	1.77 t (6.8)	41.6	1.81 t (6.8)	32.0
3″		73.9		73.9		76.3		75.9
4″	1.12 s	25.0	1.14 s	25.0	1.21 s	24.7	1.34 s	26.8
5″	1.12 s	25.0	1.14 s	25.0	1.21 s	24.7	1.34 s	26.8
1′′′	3.13 s	48.4	3.15 s	48.4	3.56 q (7.0)	57.6		
2′′′					1.29 t (7.0)	15.6		

Assignments were achieved based on HSQC and HMBC. *J* values (Hz) are given in parentheses. ^a^ Data were acquired in DMSO-*d*_6_. ^b^ Data were acquired in CDCl_3_. ^c^ 500 MHz. ^d^ 125 MHz.

**Table 3:** ^1^H- and ^13^C-NMR data for **14**–**17** and **19** in DMSO-*d*_6_.

No.	14		15		16		17		19	
C/H	δ_H_^a^ (*J*/Hz)	δ_C_ ^b^	δ_H_^a^ (*J*/Hz)	δ_C_ ^b^	δ_H_^a^ (*J*/Hz)	δ_C_ ^b^	δ_H_^a^ (*J*/Hz)	δ_C_ ^b^	δ_H_^a^ (*J*/Hz)	δ_C_ ^b^
α	3.12 t (7.3)	44.9	3.23 t (7.7)	39.5	3.23 t (7.1)	39.0	7.72 s	117.5	3.17 t (7.7)	38.9
β	2.75 t (7.3)	29.5	2.82 t (7.7)	29.2	2.82 t (7.1)	29.1	7.72 s	143.6	2.81 t (7.7)	29.3
C=O		198.6		204.6		204.6		191.1		203.8
1		131.7		130.9		130.8		125.7		131.0
2,6	7.00 d (8.1)	128.9	7.06 d (8.0)	129.2	7.06 d (8.3)	129.1	7.74 d (8.0)	131.1	7.06 d (8.2)	129.2
3,5	6.66 d (8.1)	115.0	6.67 d (8.0)	115.1	6.67 d (8.3)	115.0	6.84 d (8.0)	125.7	6.67 d (8.2)	115.0
4		155.3		155.6		155.5		160.4		155.5
1′		118.2		113.7		112.0		112.0		111.4
2′		155.2		159.4		162.1		163.8		162.3
3′		108.1		113.3		107.8		115.8		115.6
4′		161.2	12.80 s (OH)	166.8	13.18 s (OH)	159.3	14.06 s (OH)	163.8	13.13 s (OH)	162.3
5′	6.40 d (8.6)	107.0	6.41 d (8.7)	101.7	6.34 d (8.9)	108.5	6.43 d (9.0)	108.0	6.40 d (8.8)	107.9
6′	7.38 d (8.6)	129.1	7.81 d (8.7)	132.9	7.73 d (8.9)	129.7	7.98 d (9.0)	129.5	7.63 d (8.8)	129.7
1″	2.55 t (6.7)	17.0	3.04 d (8.8)	26.5	2.78 dd (17.0, 5.1) 2.46 dd (17.0, 7.0)	25.2	2.57 m	17.5	2.54 m	17.4
2″	1.76 t (6.7)	31.1	4.71 t (8.8)	91.2	3.67 t (5.1)	67.0	1.50 m	42.3	1.47 m	42.3
3″		74.7		70.1		78.4		69.0		69.0
4″	1.29 s	26.5	1.13 s	25.8	1.27 s	25.2	1.14 s	29.2	1.13 s	29.1
5″	1.29 s	26.5	1.12 s	24.8	1.20 s	20.9	1.14 s	29.2	1.13 s	29.1

Assignments were achieved based on HSQC and HMBC. *J* values (Hz) are given in parentheses. ^a^ 400 MHz. ^b^ 100 MHz.

**Table 4:** Cytotoxic activities of compounds **1**–**4** and their metabolites **5**–**21** ^a^.

Compound	IC_50_ ± SD (μM)			Compound	IC_50_ ± SD (μM)		
	A375P	HT-29	MCF-7		A375P	HT-29	MCF-7
**1**	8.09 ± 0.35	7.54 ± 0.70	9.20 ± 0.13	**12**	4.35 ± 0.35	5.77 ± 0.28	10.07 ± 1.11
**2**	25.48 ± 1.53	25.98 ± 0.72	26.99 ± 0.77	**13**	27.38 ± 0.67	60.15 ± 1.15	33.87 ± 1.80
**3**	>100	>100	>100	**14**	31.90 ± 1.27	77.65 ± 1.33	47.43 ± 1.91
**4**	5.21 ± 0.39	21.34 ± 1.40	20.94 ± 0.19	**15**	66.57 ± 1.91	>100	>100
**5**	>100	>100	>100	**16**	28.91 ± 1.99	>100	82.77 ± 1.82
**6**	>100	>100	>100	**17**	42.98 ± 0.62	>100	43.58 ± 1.39
**7**	>100	>100	>100	**18**	70.05 ± 1.27	>100	>100
**8**	29.41 ± 1.86	57.17 ± 4.28	59.44 ± 0.39	**19**	21.92 ± 2.26	70.39 ± 1.53	68.26 ± 2.28
**9**	>100	>100	>100	**20**	57.60 ± 0.67	>100	85.25 ± 1.51
**10**	14.20 ± 0.40	73.39 ± 0.48	47.23 ± 1.07	**21**	>100	>100	>100
**11**	29.38 ± 0.59	>100	61.75 ± 1.57	DZ	2.10 ± 0.06	10.13 ± 0.27	2.33 ± 0.05

^a^ Results are expressed as the mean values of three experiments ± SD; Demethylzeylasteral (DZ) was used as a positive control.

**Table 5:** Scale-up fermentation of substrates with *A. niger*.

Substrate	Substrate Amount (mg/Flask)	Number of Flasks	Total Extract Amount (g)
**1**	3	8	0.22
**2**	3	13	0.47
**3**	3	15	0.54
**4**	3	36	1.36

## Data Availability

Not applicable.

## Supplementary Materials

The following are available online at https://www.mdpi.com/article/10.3390/ijms221810109/s1.
