# Supplementary material for: Biotransformation of the Phenolic Constituents from Licorice and Cytotoxicity Evaluation of Their Metabolites
Source: Int J Mol Sci. 2021 Sep 18;22(18):10109. doi: 10.3390/ijms221810109 (PMC8465054; doi:10.3390/ijms221810109)
Supplement: Supplementary file 1 [file ijms-22-10109-s001.zip › ijms-1387881-supplementary.pdf]

Supplementary Material

Biotransformation of the Phenolic Constituents from Licorice and Cytotoxicity Evaluation of Their Metabolites

Yina Xiao [1], Fubo Han [2] and Ik-Soo Lee [2,*]

College of Pharmacy, Chonnam National University, Gwangju 61186, Korea; yogurtxiao@163.com (Y.X.); hanfubo0306@gmail.com (F.H.)

* Correspondence: islee@chonnam.ac.kr; Tel.: +82-62-530-2932

Contents of Supplementary Material

1. Spectroscopic data of **1** and **2**........5

Figure S1. ^{1}H-NMR (CD3OD, 400 MHz) spectrum of licoisoflavanone (**1**)........5

Figure S2. ^{1}H-NMR (CD3OD, 400 MHz) spectrum of glycyrrhisoflavone (**2**)........6

2. Synthetic procedures of echinatin (**3**)........7

Scheme S1. Synthetic procedure of echinatin (**3**)........7

Figure S3. ^{1}H-NMR (400 MHz, $CDCl_3$) spectrum of ECsyn-P1........8

Figure S4. ^{1}H-NMR (400 MHz, $CDCl_3$) spectrum of ECsyn-P2........8

Figure S5. ^{1}H-NMR (400 MHz, CD_3OD) spectrum of echinatin (**3**)........9

3. Synthetic procedures of isobavachalcone (**4**)........10

Scheme S2. Synthetic procedure of isobavachalcone (**4**)........10

Figure S6. ^{1}H-NMR (400 MHz, $CDCl_3$,) spectrum of IBCsyn-P1........11

Figure S7. ^{1}H-NMR (400 MHz, $CDCl_3$,) spectrum of IBCsyn-P2........11

Figure S8. ^{1}H-NMR (400 MHz, $CDCl_3$,) spectrum of IBCsyn-P3........12

Figure S9. ^{1}H-NMR (400 MHz, CD_3OD) spectrum of isobavachalcone (**4**)........12

4. Spectroscopic data of **5-21**........13

Figure S10. ^{1}H-NMR (CD_3OD, 400 MHz) spectrum of **5**........13

Figure S11. ^{13}C-NMR (CD_3OD, 100 MHz) spectrum of **5**........13

Figure S12. HSQC spectrum of **5**........14

Figure S13. HMBC spectrum of **5**........14

Figure S14. HRESIMS spectrum of **5**........15

Figure S15. CD spectrum of **5**........15

Figure S16. ^{1}H-NMR (CD_3OD, 400 MHz) spectrum of **6**........16

Figure S17. ^{13}C-NMR (CD_3OD, 100 MHz) spectrum of **6**........16

Figure S18. HSQC spectrum of **6**........17

Figure S19. HMBC spectrum of **6**........17

Figure S20. HRESIMS spectrum of **6**........18

Figure S21. ^{1}H-NMR (CD_3OD, 400 MHz) spectrum of **7**........19

Figure S22. ^{13}C-NMR (CD_3OD, 100 MHz) spectrum of **7**........19

Figure S23. HSQC spectrum of **7** 20
Figure S24. HMBC spectrum of **7** 20
Figure S25. HRESIMS spectrum of **7** 21
Figure S26. ^{1}H NMR spectrum of **8** (CD_3OD, 100 MHz) 22
Figure S27. ^{1}H NMR spectrum of **9** (CD_3OD, 100 MHz) 23
Figure S28. ^{1}H-NMR (500 MHz, DMSO-d_6) spectrum of **10** 24
Figure S29. ^{13}C-NMR (150 MHz, DMSO-d_6) spectrum of **10** 24
Figure S30. HSQC-spectrum of **10** 25
Figure S31. HMBC spectrum of **10** 25
Figure S32. COSY spectrum of **10** 26
Figure S33. HRESIMS spectrum of **10** 27
Figure S34. ^{1}H-NMR (500 MHz, DMSO-d_6) spectrum of **11** 28
Figure S35. ^{13}C-NMR (150 MHz, DMSO-d_6) spectrum of **11** 28
Figure S36. HSQC spectrum of **11** 29
Figure S37. HMBC spectrum of **11** 29
Figure S38. HRESIMS spectrum of **11** 30
Figure S39. ^{1}H-NMR (500 MHz, $CDCl_3$) spectrum of **12** 31
Figure S40. ^{13}C-NMR (150 MHz, $CDCl_3$) spectrum of **12** 31
Figure S41. HSQC spectrum of **12** 32
Figure S42. HMBC spectrum of **12** 32
Figure S43. HRESIMS spectrum of **12** 33
Figure S44. ^{1}H-NMR (500 MHz, $CDCl_3$) spectrum of **13** 34
Figure S45. ^{13}C NMR (150 MHz, $CDCl_3$) spectrum of **13** 34
Figure S46. HSQC spectrum of **13** 35
Figure S47. HMBC spectrum of **13** 35
Figure S48. HRESIMS spectrum of **13** 36
Figure S49. ^{1}H-NMR (400 MHz, DMSO-d_6) spectrum of **14** 37
Figure S50. ^{13}C-NMR (100 MHz, DMSO-d_6) spectrum of **14** 37
Figure S51. HSQC spectrum of **14** 38
Figure S52. HMBC spectrum of **14** 38

Figure S53. HRESIMS spectrum of **14** ... 39
Figure S54. ^{1}H-NMR (400 MHz, DMSO-d_6) spectrum of **15** ... 40
Figure S55. ^{13}C-NMR (100 MHz, DMSO-d_6) spectrum of **15** ... 40
Figure S56. HSQC spectrum of **15** ... 41
Figure S57. HMBC spectrum of **15** ... 41
Figure S58. HRESIMS spectrum of **15** ... 42
Figure S59. ^{1}H-NMR (400 MHz, DMSO-d_6) spectrum of **16** ... 43
Figure S60. ^{13}C-NMR (100 MHz, DMSO-d_6) spectrum of **16** ... 43
Figure S61. HSQC spectrum of **16** ... 44
Figure S62. HMBC spectrum of **16** ... 44
Figure S63. HRESIMS spectrum of **16** ... 45
Figure S64. ^{1}H-NMR (400 MHz, DMSO-d_6) spectrum of **17** ... 46
Figure S65. ^{13}C-NMR (100 MHz, DMSO-d_6) spectrum of **17** ... 46
Figure S66. HSQC spectrum of **17** ... 47
Figure S67. HMBC spectrum of **17** ... 47
Figure S68. HRESIMS spectrum of **17** ... 48
Figure S69. ^{1}H-NMR (400 MHz, DMSO-d_6) spectrum of **19** ... 49
Figure S70. ^{13}C-NMR (100 MHz, DMSO-d_6) spectrum of **19** ... 49
Figure S71. HSQC spectrum of **19** ... 50
Figure S72. HMBC spectrum of **19** ... 50
Figure S73. HRESIMS spectrum of **19** ... 51
Figure S74. ^{1}H-NMR (400 MHz, DMSO-d_6) spectrum of **18** ... 52
Figure S75. ^{1}H-NMR (500 MHz, DMSO-d_6) spectrum of **20** ... 53
Figure S76. ^{1}H-NMR (400 MHz, DMSO-d_6) spectrum of **21** ... 54

1. Spectroscopic data of **1** and **2**

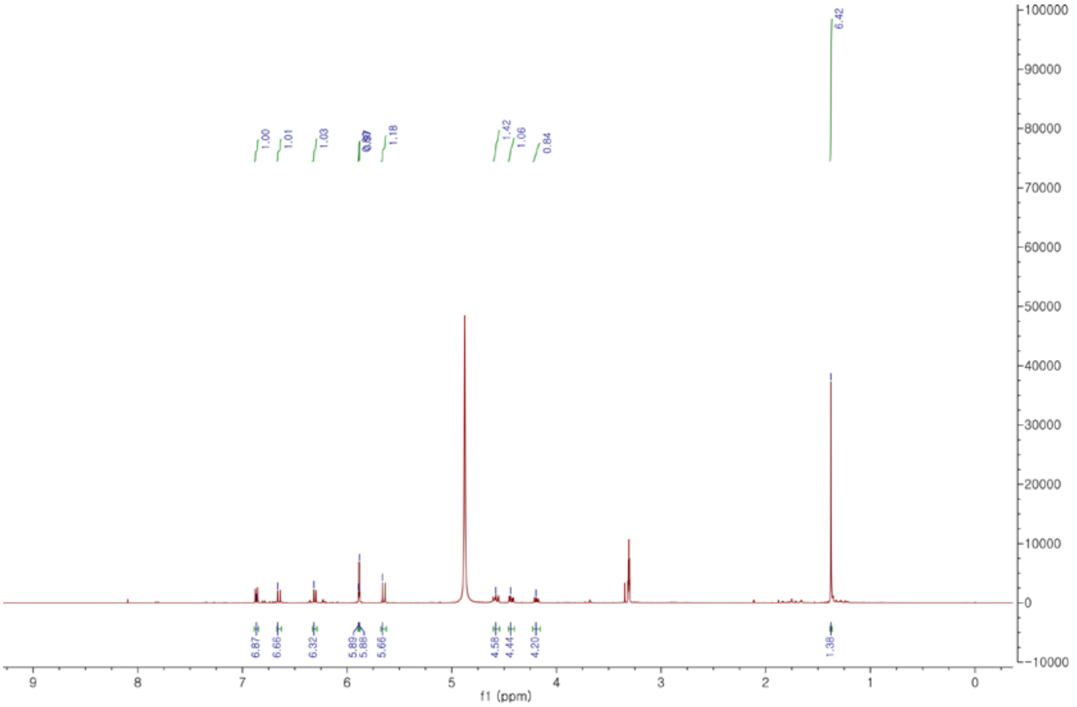

Figure S1. ^{1}H-NMR (CD_3OD, 400 MHz) spectrum of licoisoflavanone (**1**)

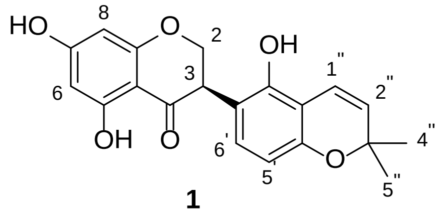 **1**	^{1}H-NMR (CD_3OD, 400 MHz, δ in ppm, *J* in Hz) δ 6.87 (1H, d, *J* = 8.3, H-6′), 6.66 (1H, d, *J* = 10.0, H-1′′), 6.32 (1H, d, *J* = 8.3, H-5′), 5.89 (1H, d, *J* = 2.2, H-6), 5.88 (1H, d, *J* = 2.2, H-8), 5.66 (1H, d, *J* = 10.0, H-2′′), 4.58 (1H, t, *J* = 10.2, H-2a), 4.44 (1H, dd, *J* = 10.2, 5.4, H-2b), 4.20 (1H, dd, *J* = 10.2, 5.4, H-3), 1.38 (6H, s, H-4′′,5′′).

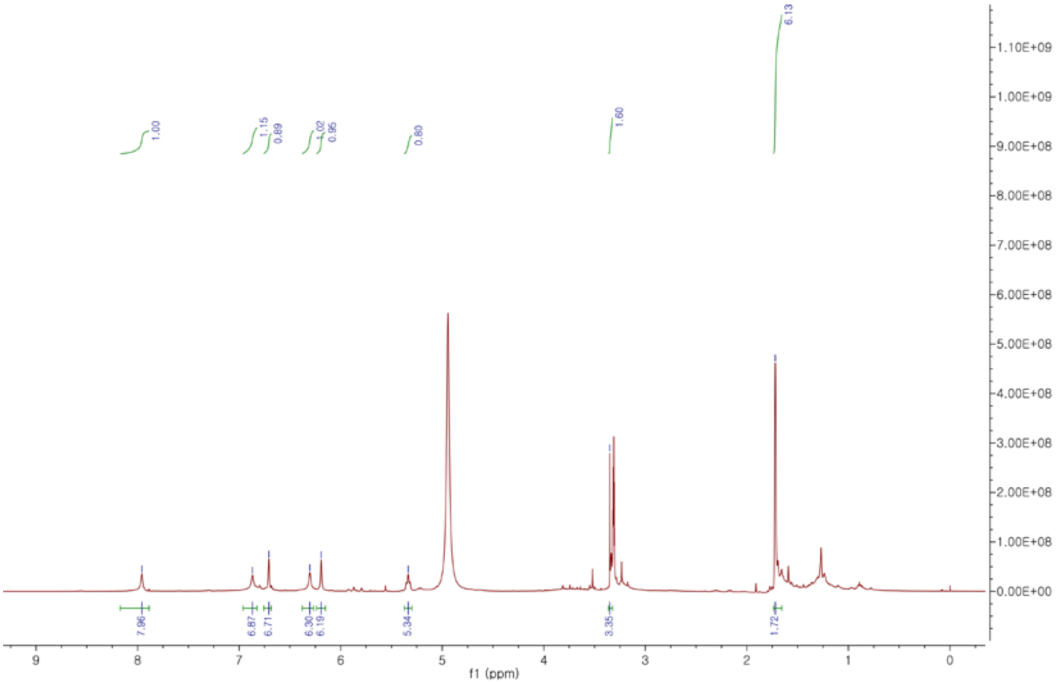

Figure S2. 1H-NMR (CD_3OD, 400 MHz) spectrum of glycyrrhisoflavone (**2**)

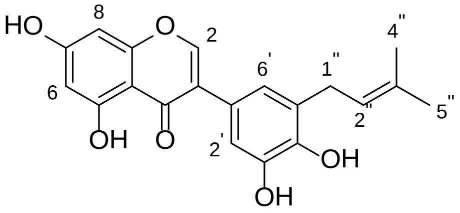 **2**	^{1}H-NMR (CD_3OD, 400 MHz, δ in ppm, *J* in Hz) δ 7.96 (1H, s, H-2), 6.87 (1H, s, H-6′), 6.71 (1H, s, H-2′), 6.30 (1H, s, H-8), 6.19 (1H, s, H-6), 5.34 (1H, m, H-2′′), 3.35 (2H, d, *J* = 7.3, H-1′′), 1.72 (6H, s, H-4′′,5′′).

2. Synthetic procedures of echinatin (**3**)

Echinatin (**3**) was synthesized through acid-mediate Claisen-Schmidt condensation using 4′-hydroxya cetophenone with 2,4-dihydroxybenzaldehyde (Scheme S1).

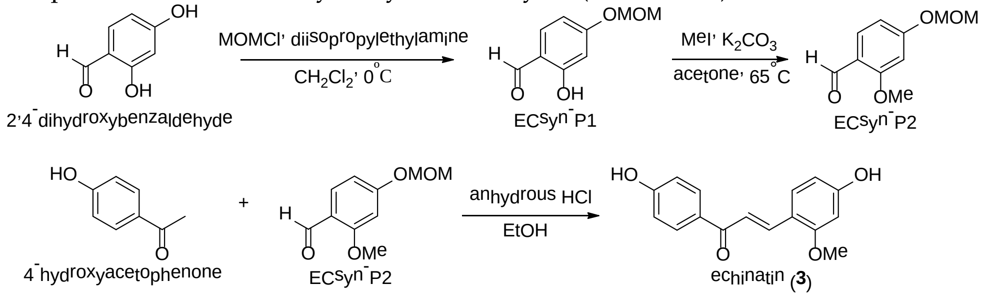

Scheme S1. Synthetic procedure of echinatin (**3**)

N,N-Diisopropylethylamine (0.9 mL) was added to a stirred solution of 2,4-dihydroxybenzaldehyde (0.5 g, 3.6 mmol) in 8 mL CH_2Cl_2 and stirred for 15 min at 0 °C. Then methoxymethylchloride (0.4 mL, 5.4 mmol) was dropped. After stirring at 0 °C for another 15 min, the reaction was kept at room temperature for 1h. The reaction was stopped by pouring water and extracted with CH_2Cl_2. The organic layer was concentrated and subjected to chromatography using silica gel (*n*-hexane: EtOAc = 15:1) to afford ECsyn-P1 (0.56 g, 85%;). ECsyn-P1: colorless oil; ^{1}H-NMR ($CDCl_3$, 400 MHz, δ in ppm, *J* in Hz) δ 11.36 (OH), 9.74 (1H, s), 7.46 (1H, d, *J* = 8.7), 6.66 (1H, dd, *J* = 8.7, 2.3), 6.60 (1H, d, *J* = 2.3), 5.22 (2H, s), 3.43 (3H, s).

To a stirred solution of ECsyn-P1 (0.5 g, 2.7 mmol) in 15 mL acetone was added slowly K_2CO_3 (0.52 g, 2.8 mmol) under nitrogen atmosphere and stirred for 10 min at 50 °C. MeI (1.7 mL) was added slowly to this reaction mixture and stirred for 3.5 h. After completion of reaction, the solvent was evaporated. The reaction mixture was suspended in water and extracted with EtOAc three times. The organic layer was concentrated and purified by silica gel column chromatography (*n*-hexane-EtOAc = 8:1) to afford ECsyn-P2 (0.49 g, 95%). ECsyn-P2: colorless oli; ^{1}H-NMR ($CDCl_3$, 400 MHz, δ in ppm, *J* in Hz) δ 10.31 (1H, s), 7.79 (1H, d, *J* = 8.6), 6.69 (1H, dd, *J* = 8.6, 2.2), 6.61 (1H, d, *J* = 2.2), 5.24 (2H, s), 3.91 (3H, s), 3.50 (3H, s).

4′-Hydroxyacetophenone (0.39 g, 2.8 mmol) and intermediate ECsyn-P2 (0.45 g, 2.3 mmol) were dissolved in 5 mL anhydrous EtOH cooled by ice-water bath, then 4 mL 1.25 M HCl in anhydrous EtOH was added slowly to the stirred solution. The mixture was continuously stirred for 16 h at 0 °C. Then the mixture was kept stirring at room temperature for 10 h. The mixture was extracted with EtOAc and the organic layer was concentrated and purified by silica gel (*n*-hexane:EtOAc = 10:1) to give a yellow solid echinatin (0.37g, 60%). Echinatin (**3**): yellow solid; ^{1}H-NMR (CD_3OD, 400 MHz, δ in ppm, *J* in Hz) δ 8.03 (1H, d, *J* = 15.6, H-*α*), 7.97 (2H, d, *J* = 8.8, H-2′,6′), 7.62 (1H, d, *J* = 15.6, H-*α*), 7.61 (1H, d, *J* = 8.5, H-6), 6.89 (2H, d, *J* = 8.8, H-3′,5′), 6.47 (1H, d, *J* = 2.2, H-3), 6.44 (1H, dd, *J* = 8.5, 2.2, H-5), 3.89 (3H, s, 2-OMe).

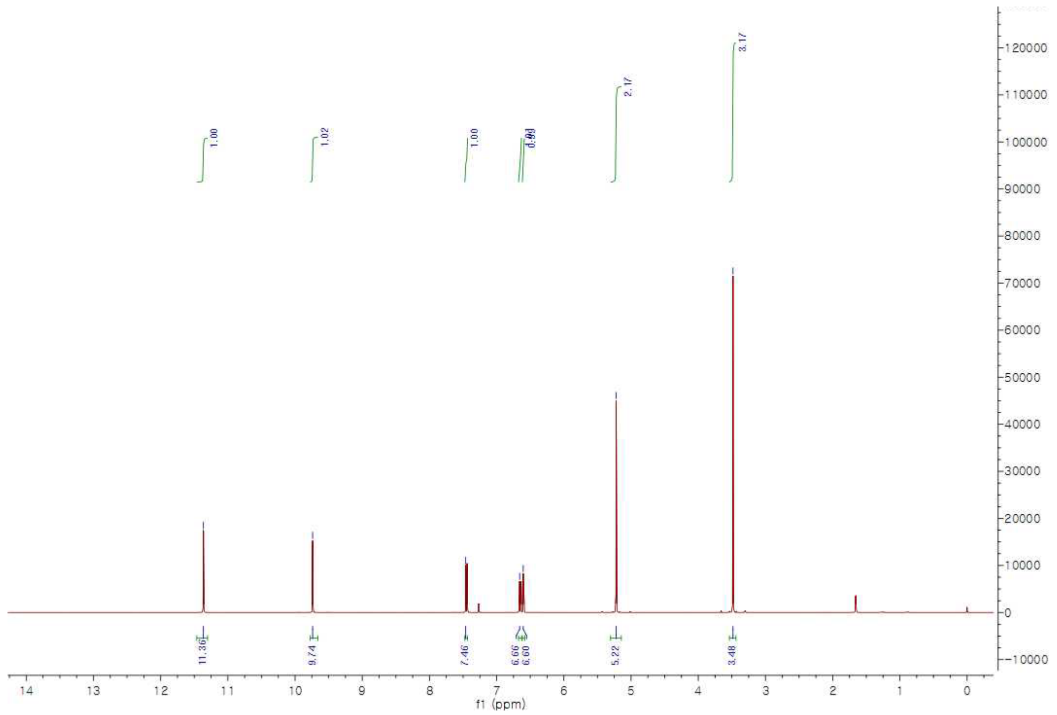

Figure S3. ^{1}H-NMR (400 MHz, $CDCl_3$) spectrum of ECsyn-P1

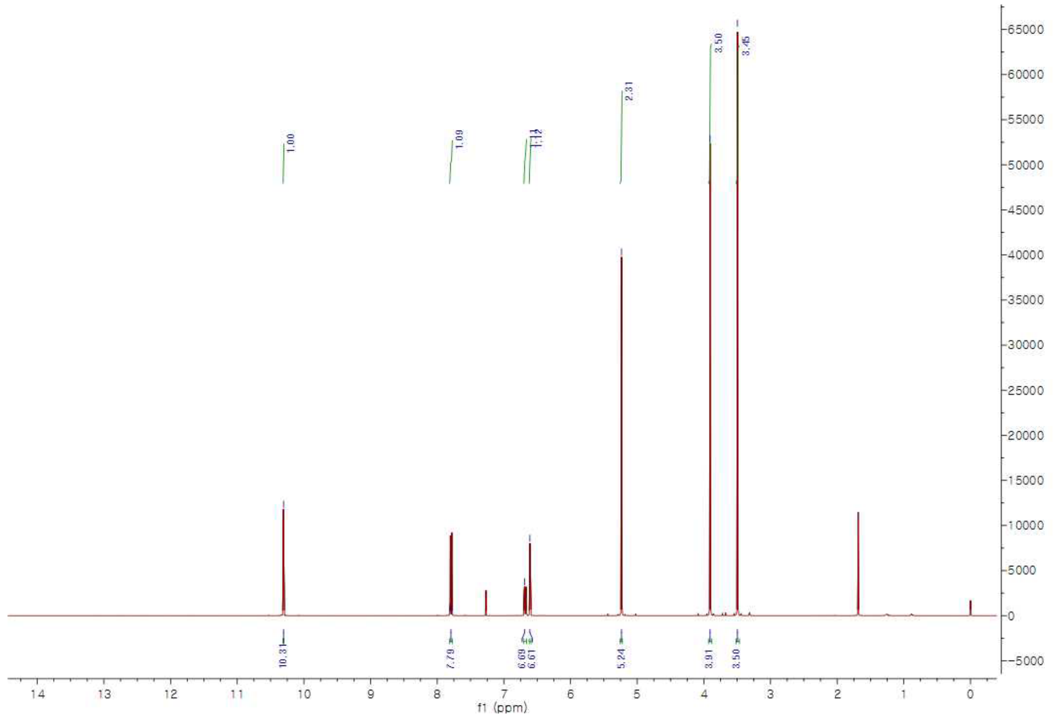

Figure S4. ^{1}H-NMR (400 MHz, $CDCl_3$) spectrum of ECsyn-P2

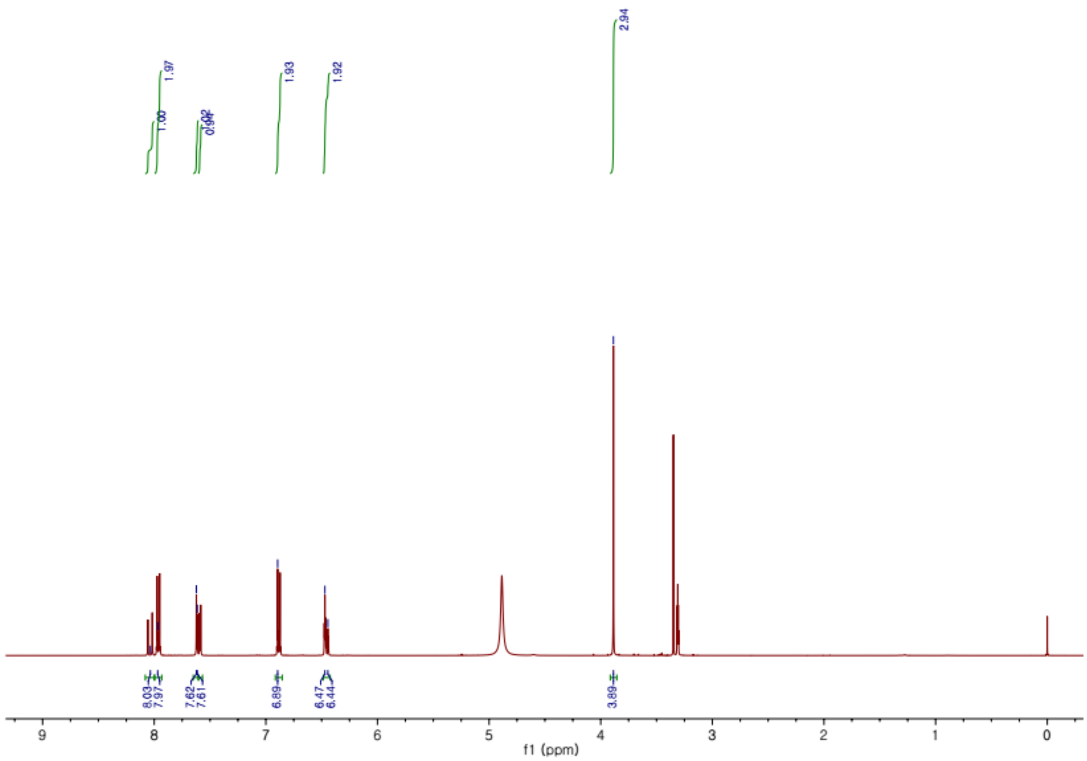

Figure S5. ^{1}H-NMR (400 MHz, CD_3OD) spectrum of echinatin (**3**)

3. Synthetic procedures of isobavachalcone (**4**)

Isobavachalcone (**4**) was synthesized through Claisen-Schmidt condensations using 2′,4′-dihydroxyacetophenone with 4-hydroxybenzaldehyde as starting materials (Scheme S2).

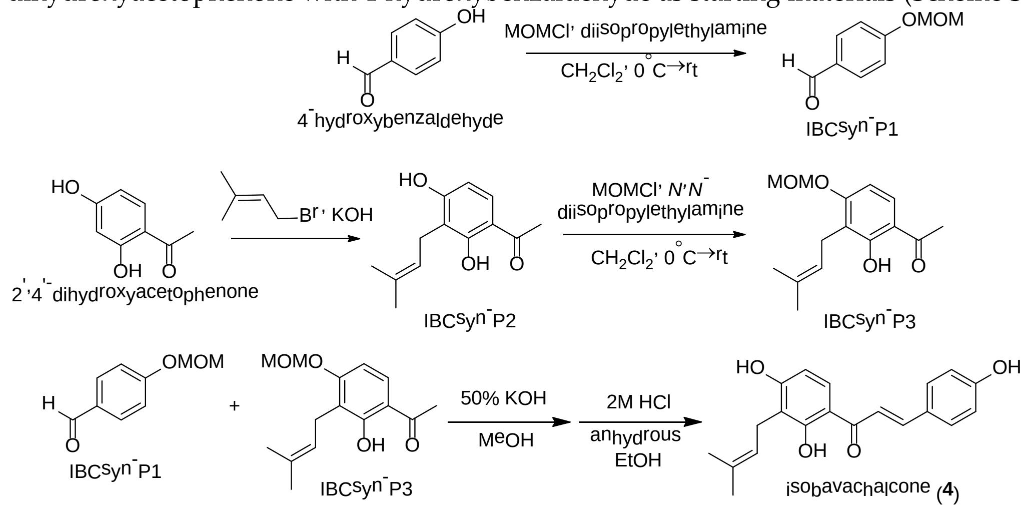

Scheme S2. Synthetic procedure of isobavachalcone (**4**)

N,N-Diisopropylethylamine (1.0 mL, 6.1 mmol) was added to a stirred solution of 4-hydroxybenzaldehyde (0.5 g, 4.1 mmol) in 10 mL CH_2Cl_2 and stirred for 15 min at 0 °C. Then methoxymethylchloride (0.5 mL, 6.1 mmol) was dropped. After stirring at 0 °C for another 15 min, the reaction was kept at room temperature for 1h. The reaction was stopped by pouring water and extracted with CH_2Cl_2. The organic layer was concentrated and subjected to chromatography using silica gel (*n*-hexane: EtOAc = 15:1) to afford IBCsyn-P1 (0.6 g, 90%). IBCsyn-P1: colorless oil; ^{1}H-NMR ($CDCl_3$, 400 MHz, δ in ppm, *J* in Hz) δ 9.90 (1H, s), 7.85 (2H, d, *J* = 8.8), 7.16 (2H, d, *J* = 8.8), 5.26 (2H, s), 3.49 (3H, s).

To a heated solution of 2′,4′-dihydroxyacetophenone (10 g, 65.7 mmol) in water (80 mL), KOH (7.4 g, 131.5 mmol) dissolved in 20 mL water was added. Prenyl bromide (11 mL, 84.6 mmol) was added dropwise and the solution was stirred at 40 °C for 5 h. After the reaction was further stirred at room temperature for 36 h, the solution was acidified with 10% HCl to pH 5.0 and extracted with EtOAc (3 × 100 mL). The organic layer was evaporated under reduced pressure and chromatographed on silica gel (*n*-hexane: EtOAc = 40:1) to afford the pure product IBCsyn-P2 (0.4 g, 2.7%). IBCsyn-P2: white solid; ^{1}H-NMR ($CDCl_3$, 400 MHz, δ in ppm, *J* in Hz) δ 13.09 (OH), 7.55 (1H, d, *J* = 8.8), 6.39 (1H, d, *J* = 8.8), 5.26 (1H, m), 3.45 (2H, d, *J* = 7.5), 2.56 (3H, s), 1.88 (3H, s), 1.76 (3H, s).

To obtain IBCsyn-P3, the method was same as used to synthesis IBCsyn-P1. Reagents: *N,N*-Diisopropylethylamine (0.5 mL, 2.9 mmol), IBCsyn-P2 (0.4 g, 1.8 mmol), methoxymethylchloride (0.2 mL, 2.7 mmol), CH_2Cl_2 (5 mL). IBCsyn-P3: 0.3 g, 64%); colorless oil; ^{1}H-NMR ($CDCl_3$, 400 MHz, δ in ppm, *J* in Hz) δ 12.78 (OH), 7.58 (1H, d, *J* = 8.9), 6.66 (1H, d, *J* = 8.9), 5.27 (2H, s), 5.21 (1H, m), 3.47 (3H, s), 3.39 (2H, d, *J* = 6.9), 2.56 (3H, s), 1.79 (3H, s), 1.67 (3H, s).

To a stirred solution of IBCsyn-P1 (150 mg, 0.8 mmol) and IBCsyn-P3 (260 mg, 1.0 mmol) in 3 mL MeOH, 50% KOH in H_2O (2 mL) was added. After stirring for 4 h at 45 °C, the reaction mixture was acidified with 10% HCl to pH 6.0 and extracted with EtOAc. After concentration, 1.5 M HCl in anhydrous EtOH (2 mL) was added to the extract at room temperature. The reaction mixture was further stirred for 1 h. Then the reaction mixture was basified with 50% KOH to pH 6.0 and extracted with EtOAc. The organic layer was evaporated and the obtained crude product was column chromatographed using Sephadex LH-20 (MeOH) to afford the pure product isobavachalcone (120 mg, 40%). Isobavachalcone (**4**): yellow solid; ^{1}H-NMR (CD_3OD, 400 MHz, δ in ppm, *J* in Hz) δ 7.84 (1H, d, *J* = 8.9, H-6'), 7.78 (1H, s, *J* = 15.4, H-β), 7.64 (1H, d, *J* = 15.4, H-α), 7.62 (2H, d, *J* = 8.6, H-2,6), 6.85 (2H, d, *J*= 8.6, H-3,5), 6.43 (1H, d, *J* = 8.9, H-5'), 5.23 (1H, m, H-2''), 3.33 (2H, overlapped, H-1''), 1.78 (3H, s, H-4''), 1.66 (3H, s, H-5'').

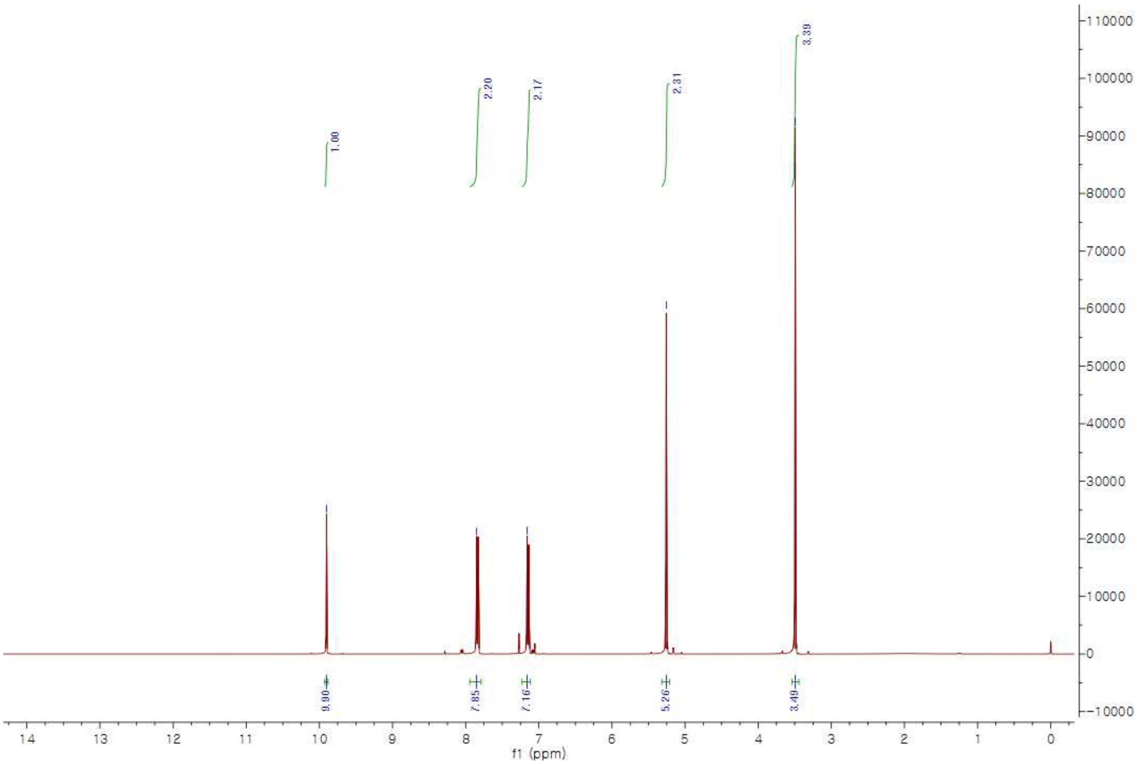

Figure S6. ^{1}H-NMR (400 MHz, $CDCl_3$,) spectrum of IBCsyn-P1

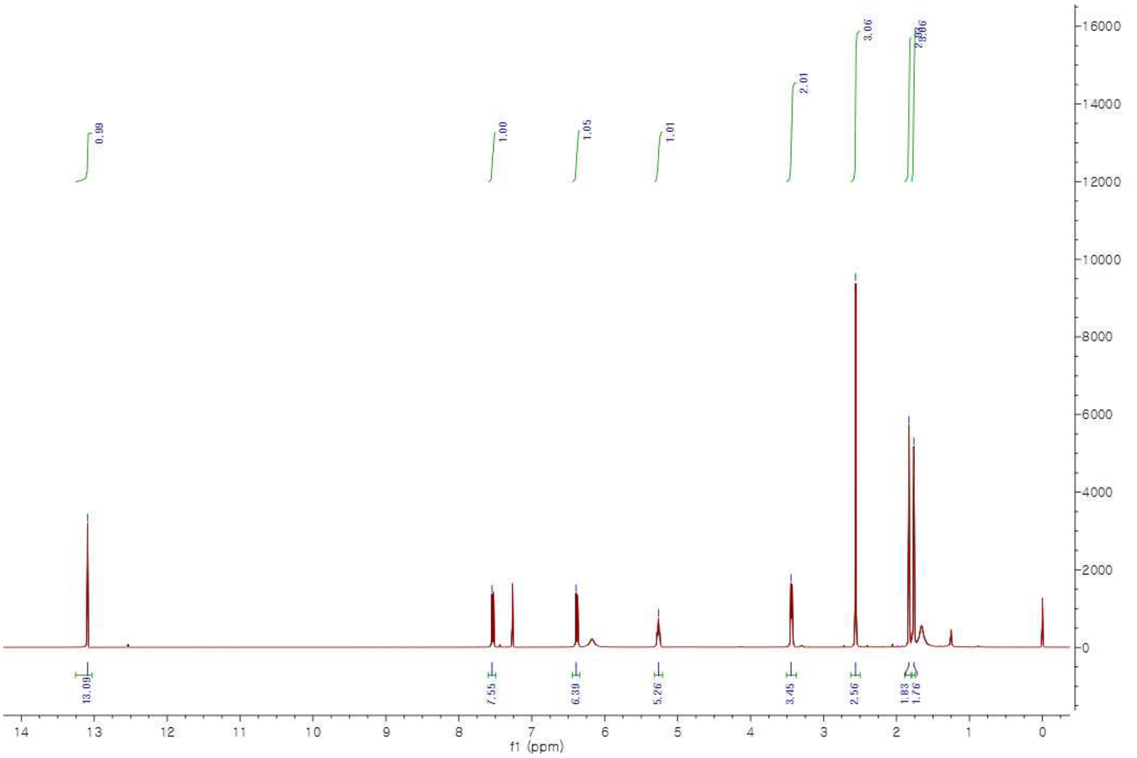

Figure S7. ^{1}H-NMR (400 MHz, $CDCl_3$,) spectrum of IBCsyn-P2

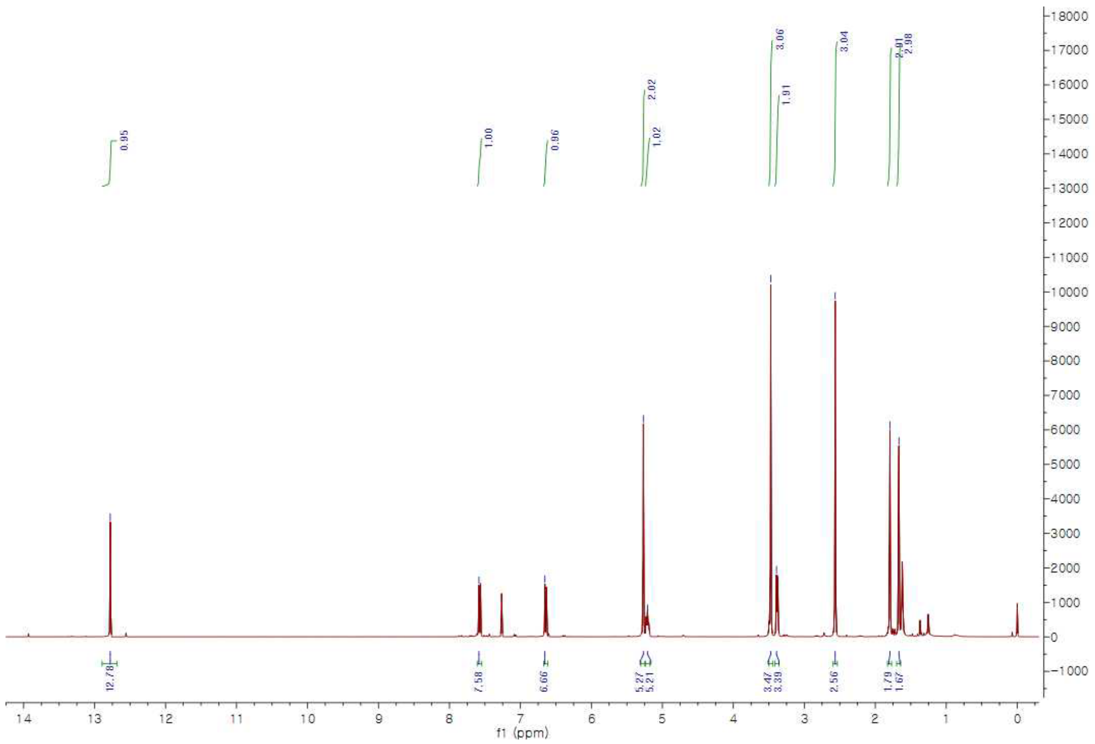

Figure S8. ^{1}H-NMR (400 MHz, $CDCl_3$,) spectrum of IBCsyn-P3

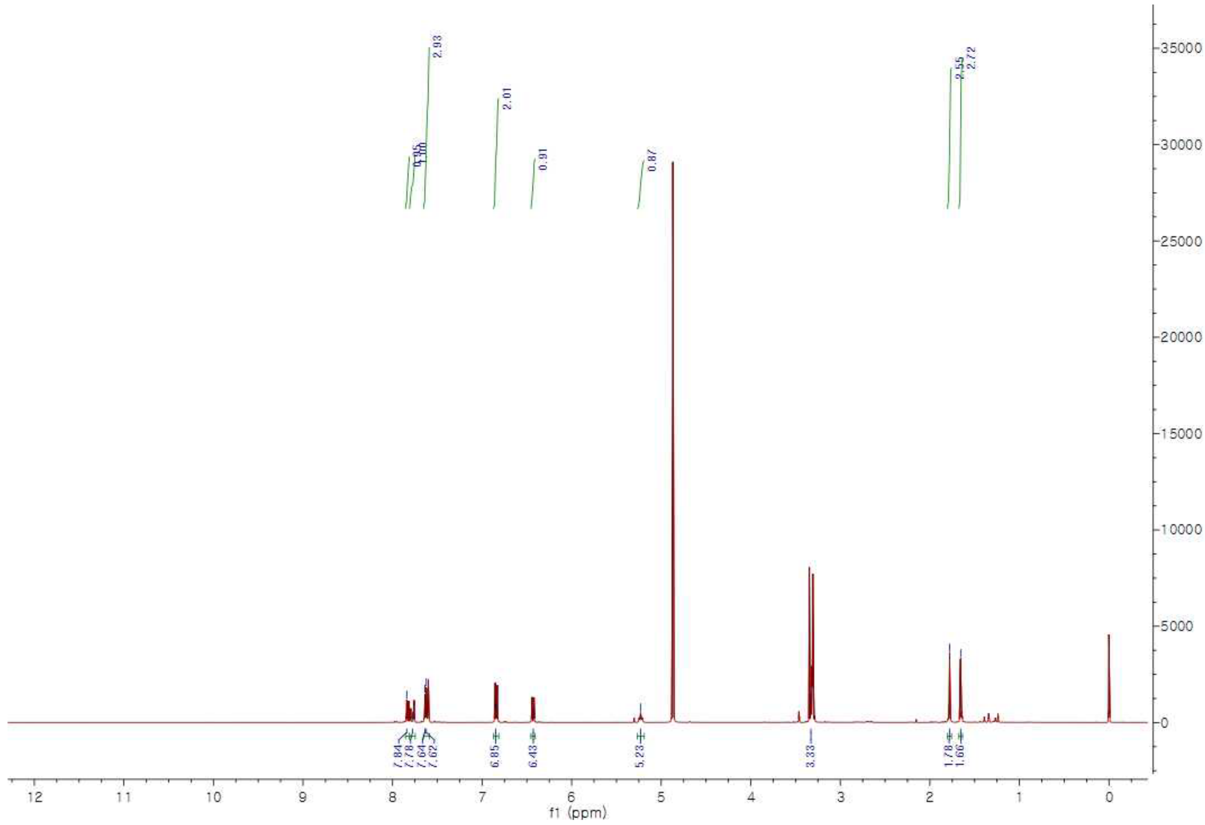

Figure S9. ^{1}H-NMR (400 MHz, CD_3OD) spectrum of isobavachalcone (**4**)

4. Spectroscopic data of **5-21**

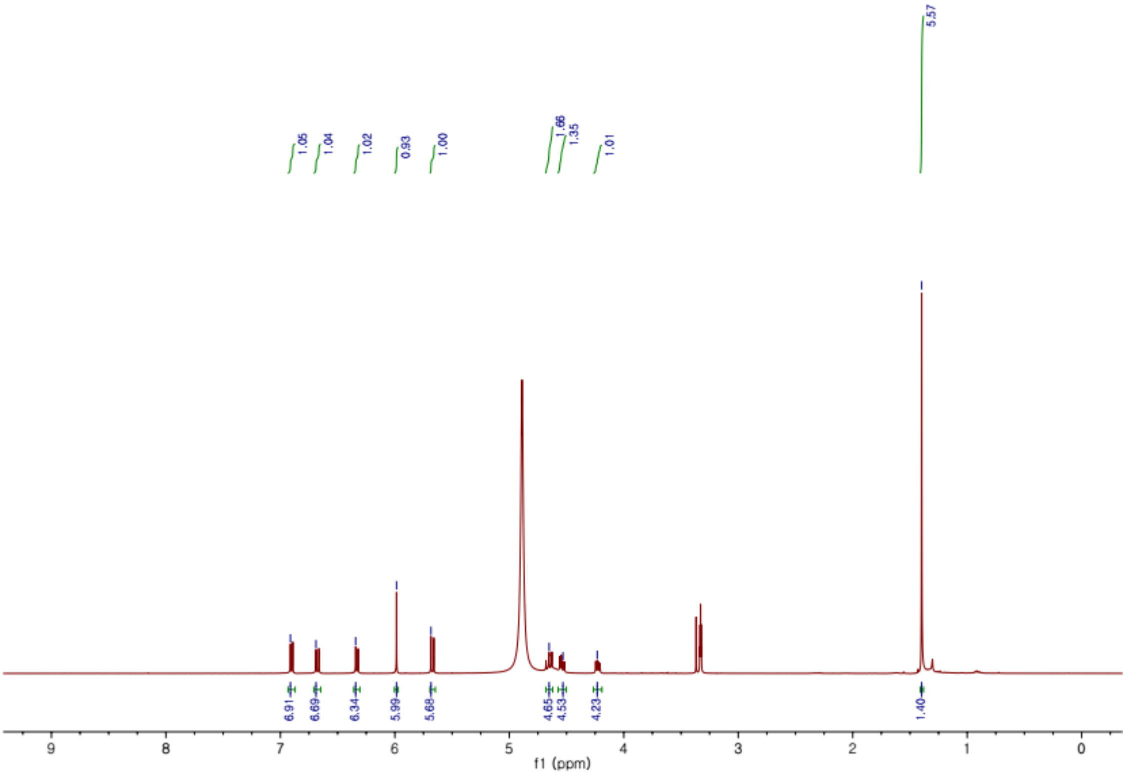

Figure S10. ^{1}H-NMR (CD3OD, 400 MHz) spectrum of **5**

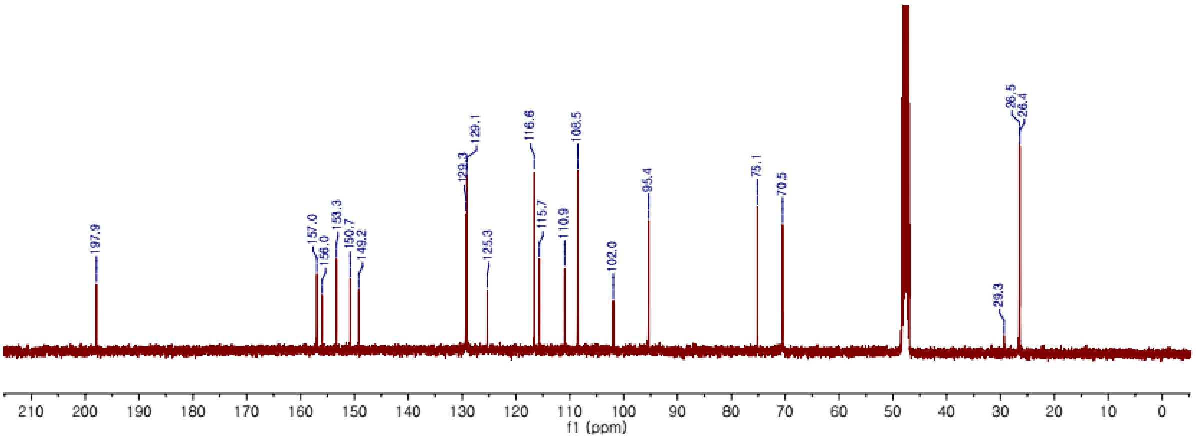

Figure S11. ^{13}C-NMR (CD3OD, 100 MHz) spectrum of **5**

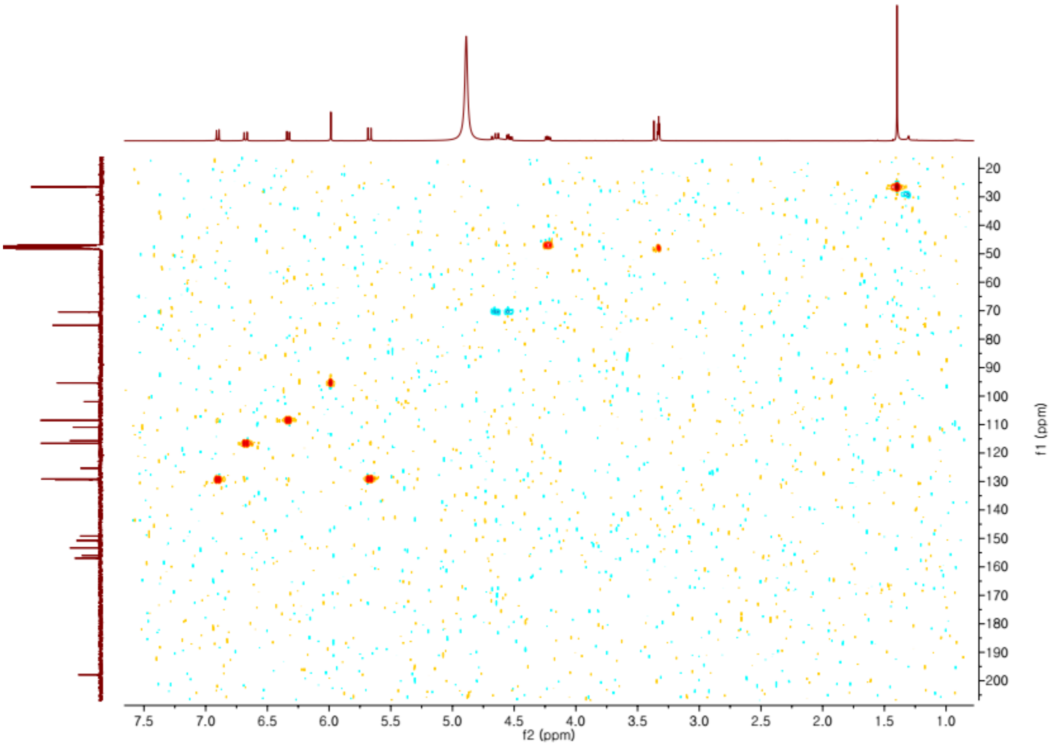

Figure S12. HSQC spectrum of **5**

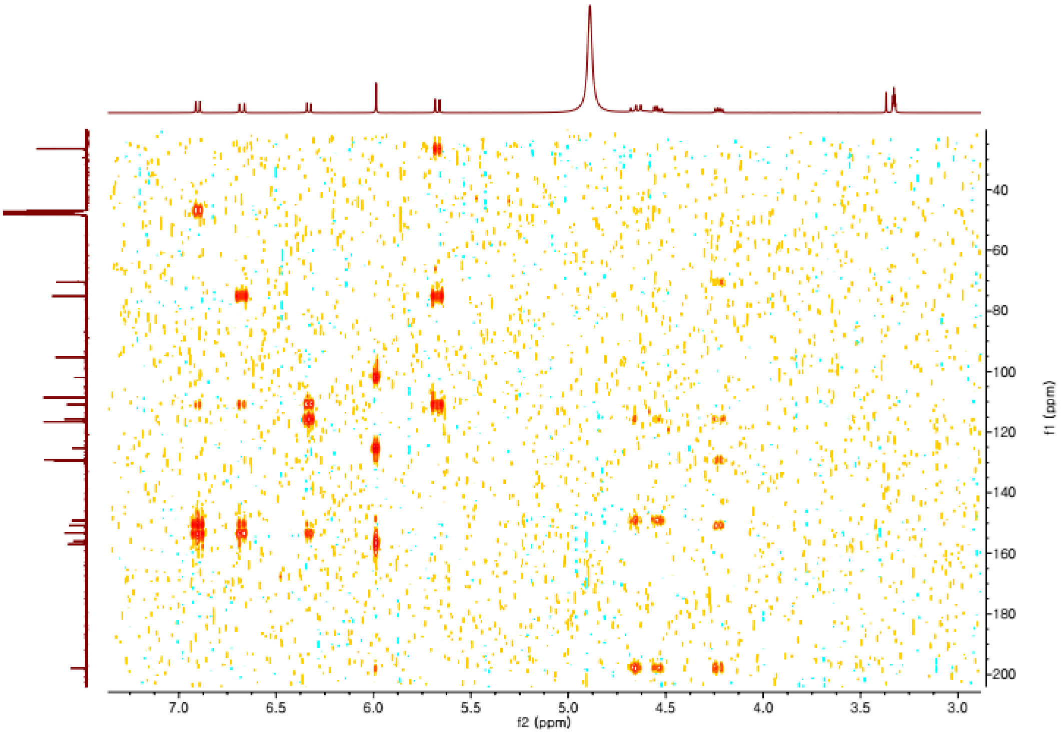

Figure S13. HMBC spectrum of **5**

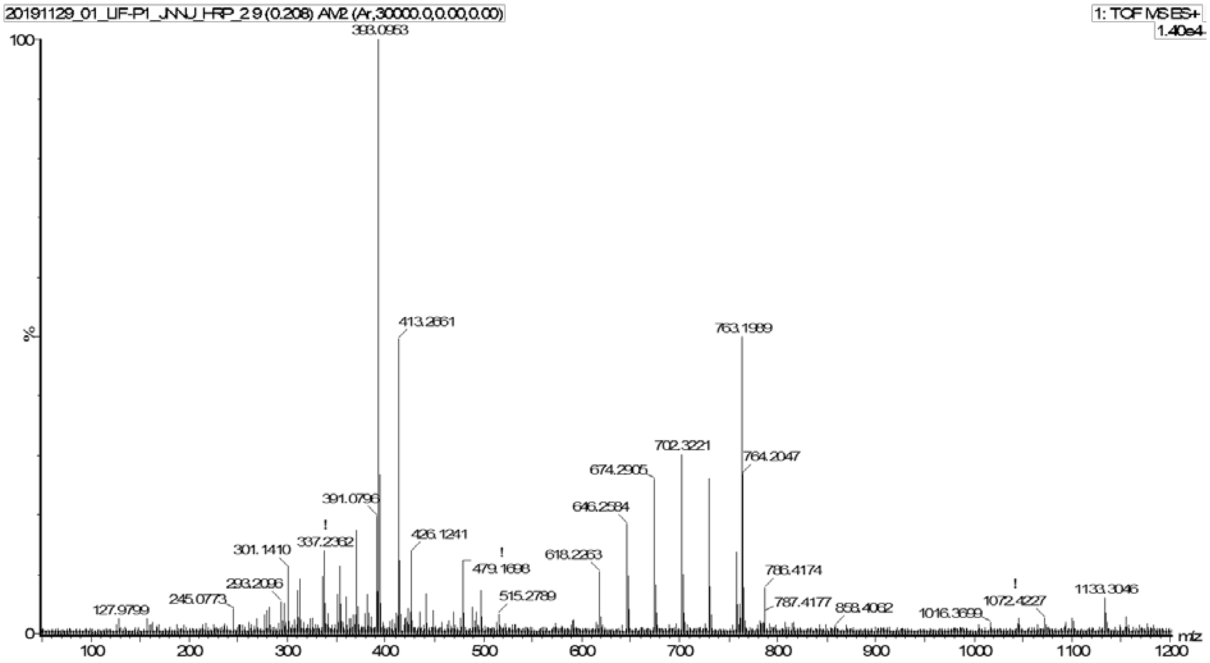

Elemental Composition Report

Elemental Composition Report

Single Mass Analysis
Tolerance = 5.0 PPM / DBE: min = -1.5, max = 100.0
Element prediction: Off
Number of isotope peaks used for i-FIT = 3

Monoisotopic Mass, Even Electron Ions
132 formula(e) evaluated with 1 results within limits (up to 100 closest results for each mass)
Elements Used:
C: 1-40 H: 1 -50 O: 1 -20 Na: 0 -1

Minimum:				-1.5				
Maximum:		100.0	5.0	100.0				
Mass	Calc. Mass	mDa	PPM	DBE	i-FIT	Norm	Conf(%)	Formula
393.0953	393.0950	0.3	0.8	11.5	551.0	n/a	n/a	C20 H18 O7 Na

Figure S14. HRESIMS spectrum of **5**

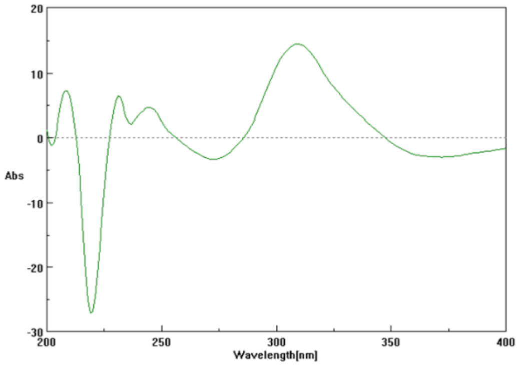

Figure S15. CD spectrum of **5**

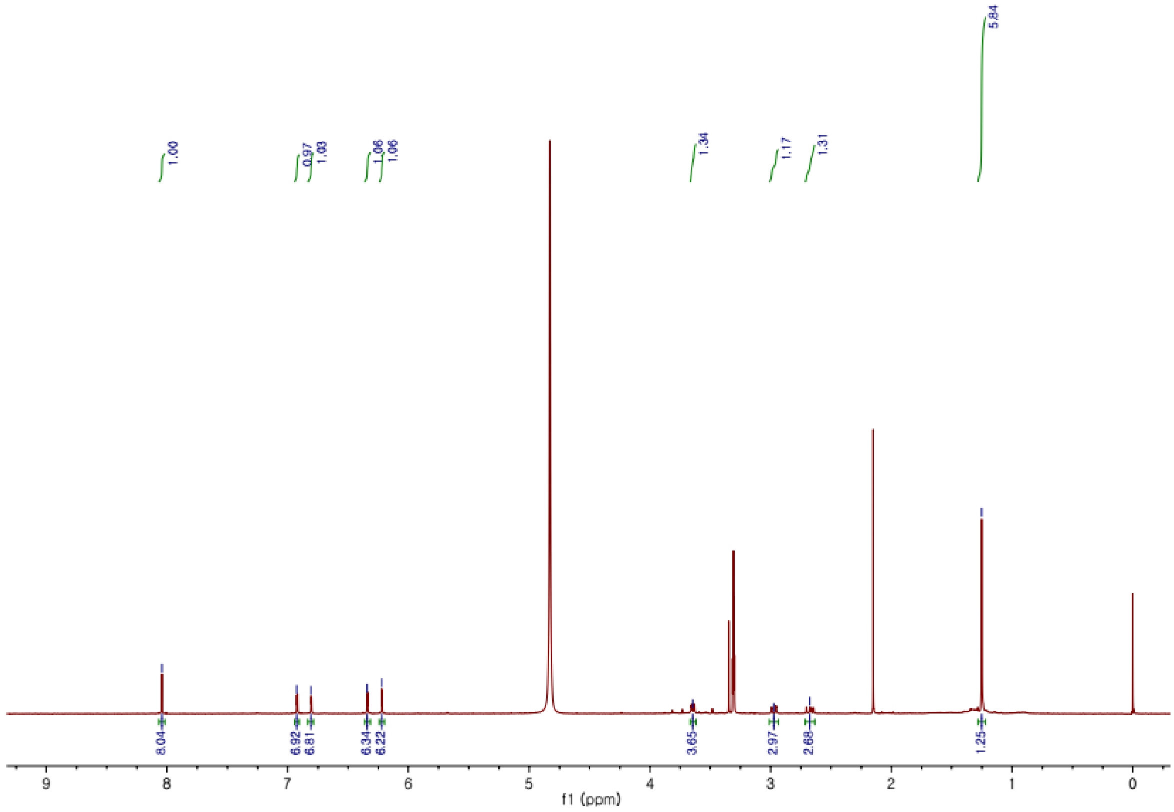

Figure S16. ^{1}H-NMR (CD_3OD, 400 MHz) spectrum of **6**

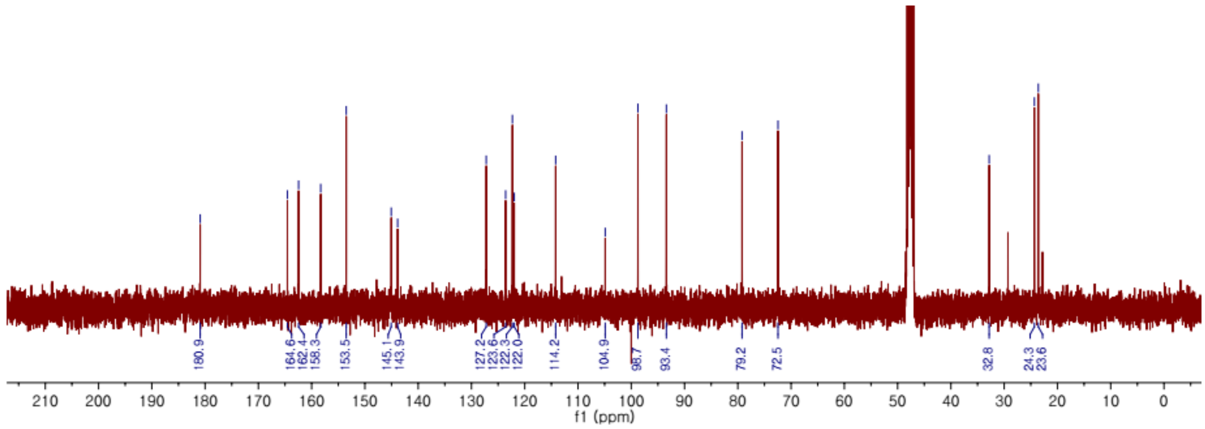

Figure S17. ^{13}C-NMR (CD_3OD, 100 MHz) spectrum of **6**

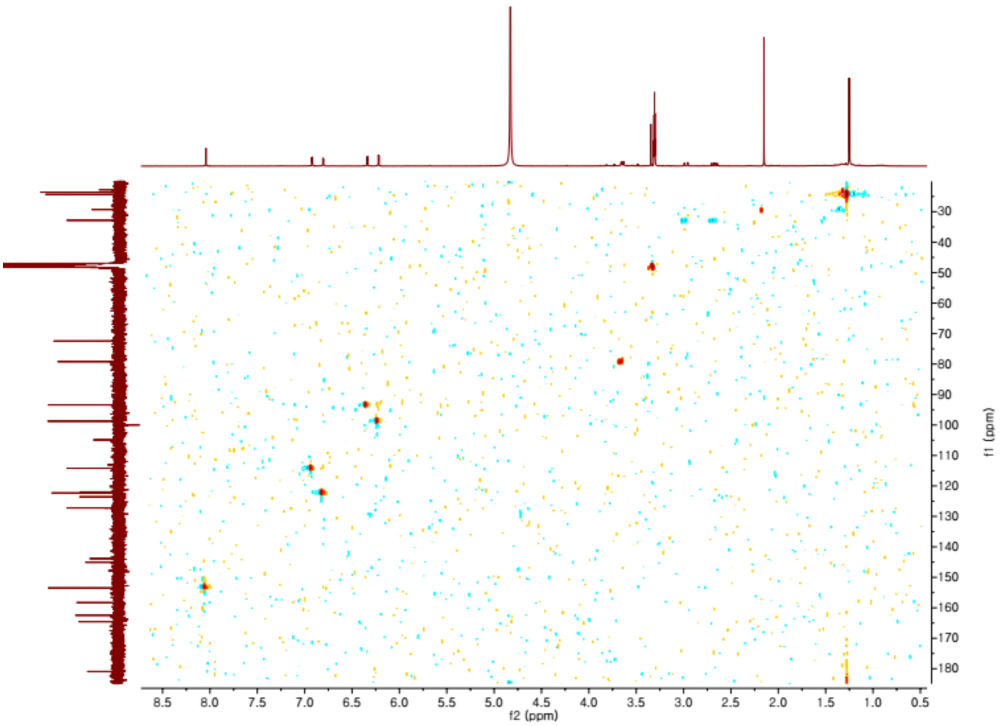

Figure S18. HSQC spectrum of **6**

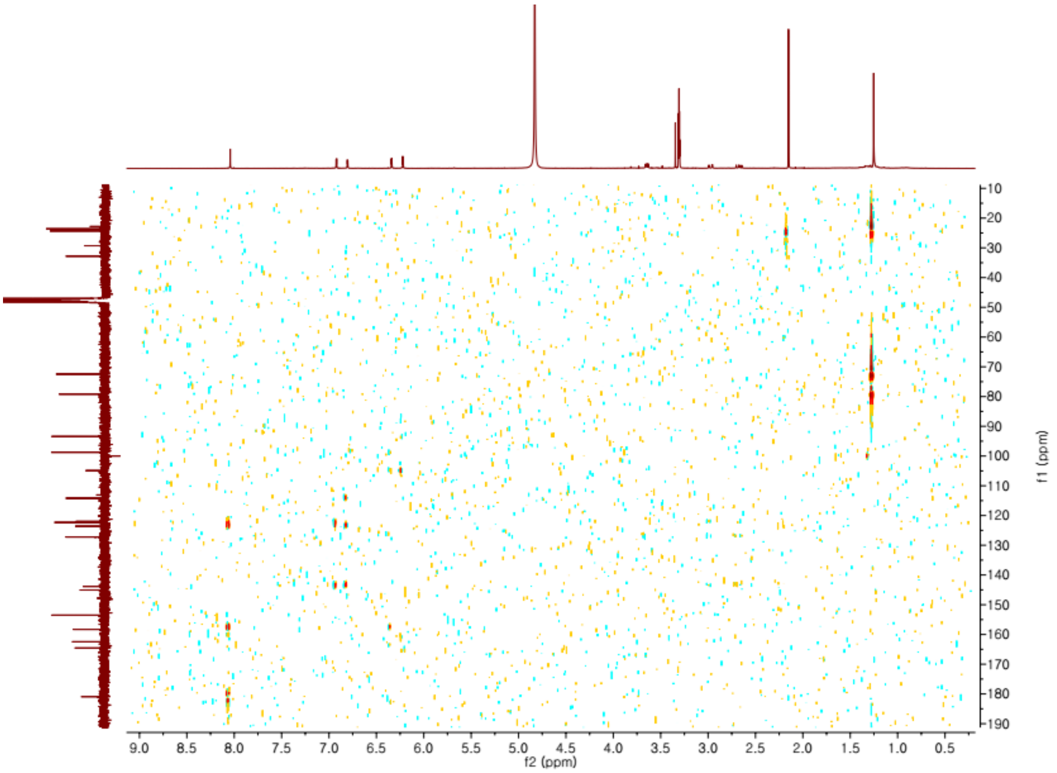

Figure S19. HMBC spectrum of **6**

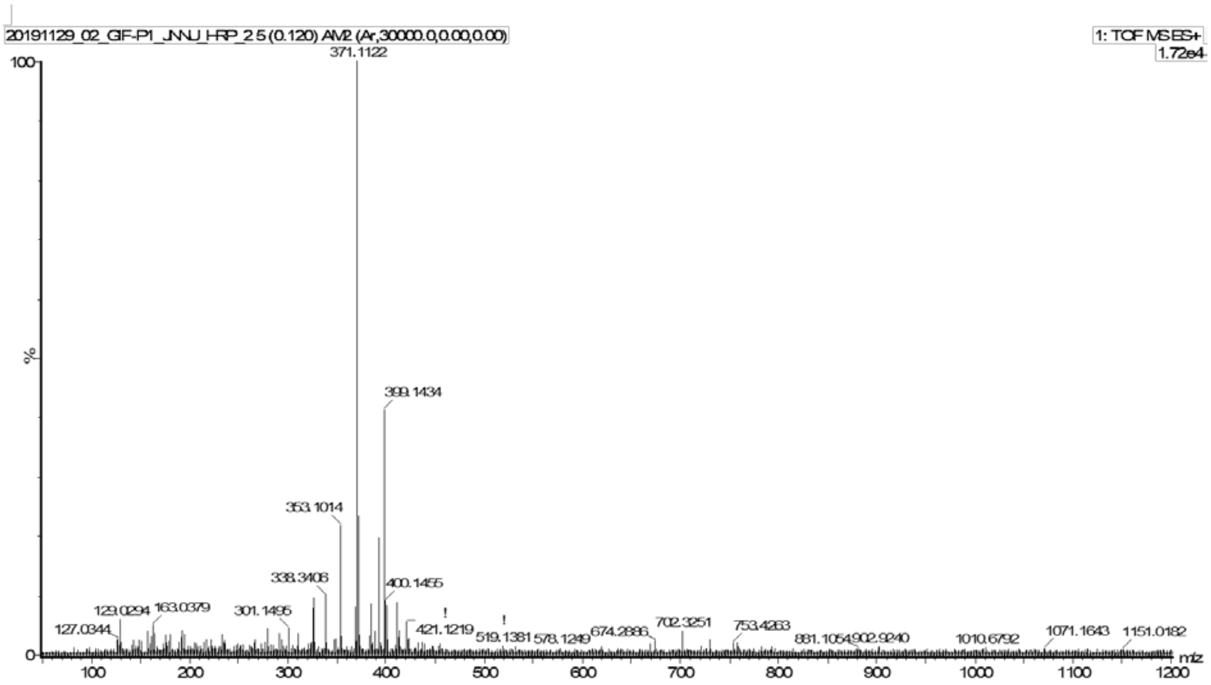

Elemental Composition Report

Single Mass Analysis
Tolerance = 5.0 PPM / DBE: min = -1.5, max = 100.0
Element prediction: Off
Number of isotope peaks used for i-FIT = 3

Monoisotopic Mass, Even Electron Ions
128 formula(e) evaluated with 2 results within limits (up to 100 closest results for each mass)
Elements Used:
C: 1-40 H: 1 -50 O: 1 -20 Na: 0 -1

Minimum:				-1.5				
Maximum:		100.0	5.0	100.0				
Mass	Calc. Mass	mDa	PPM	DBE	i-FIT	Norm	Conf(%)	Formula
411.1060	411.1056	0.4	1.0	10.5	439.2	1.193	30.32	C20 H20 O8 Na
	411.1080	-2.0	-4.9	13.5	438.4	0.361	69.68	C22 H19 O8

Figure S20. HRESIMS spectrum of **6**

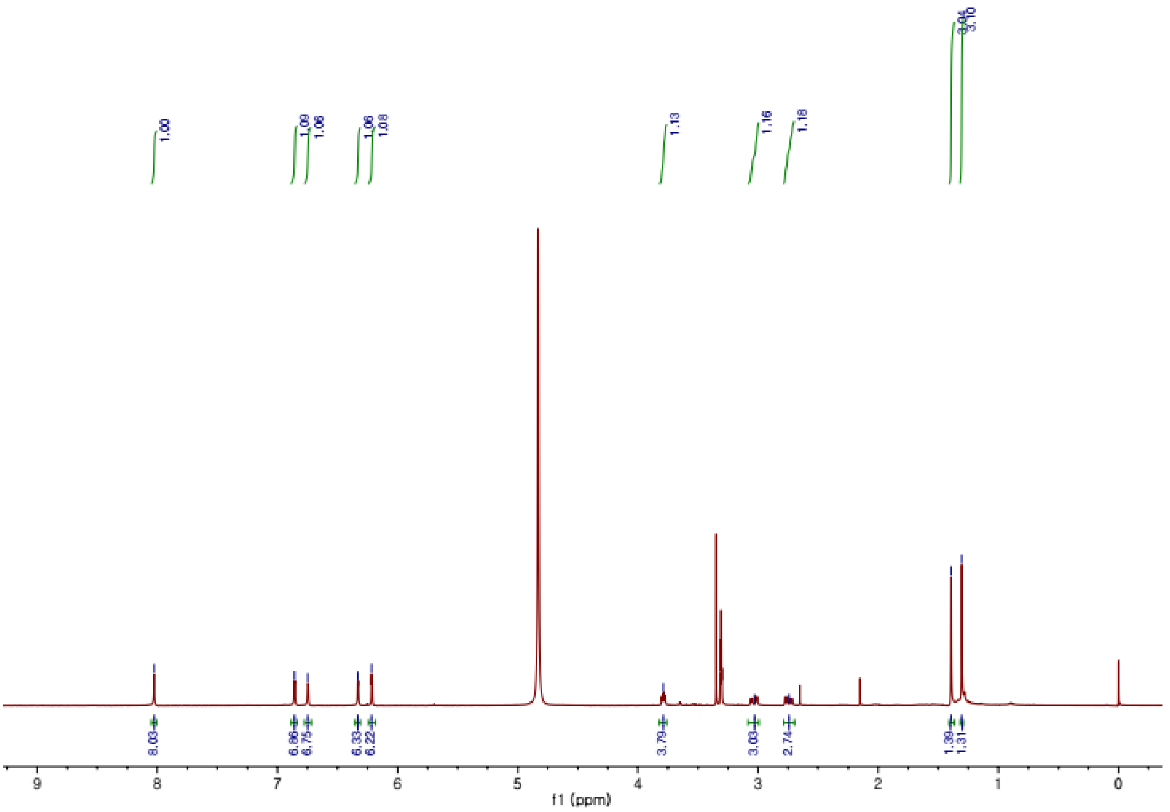

Figure S21. ^{1}H-NMR (CD_3OD, 400 MHz) spectrum of **7**

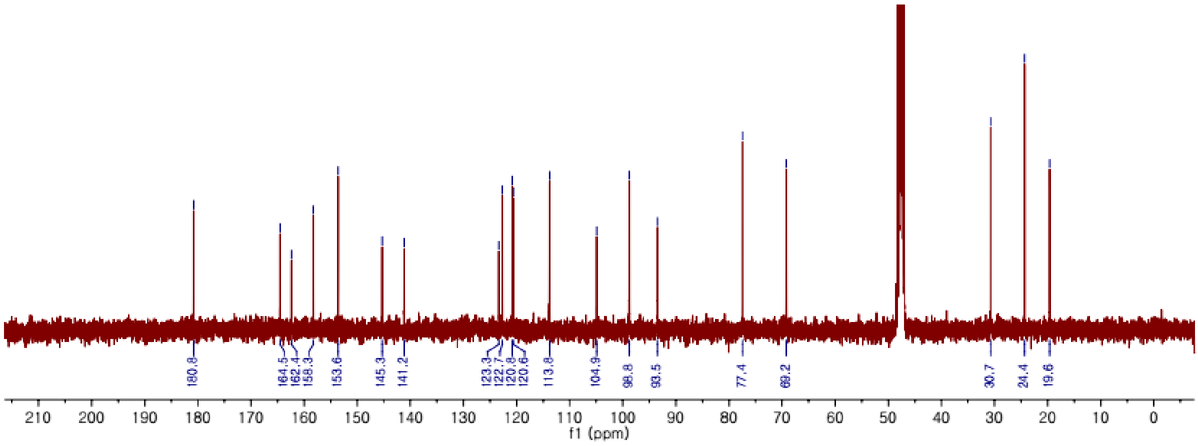

Figure S22. ^{13}C-NMR (CD_3OD, 100 MHz) spectrum of **7**

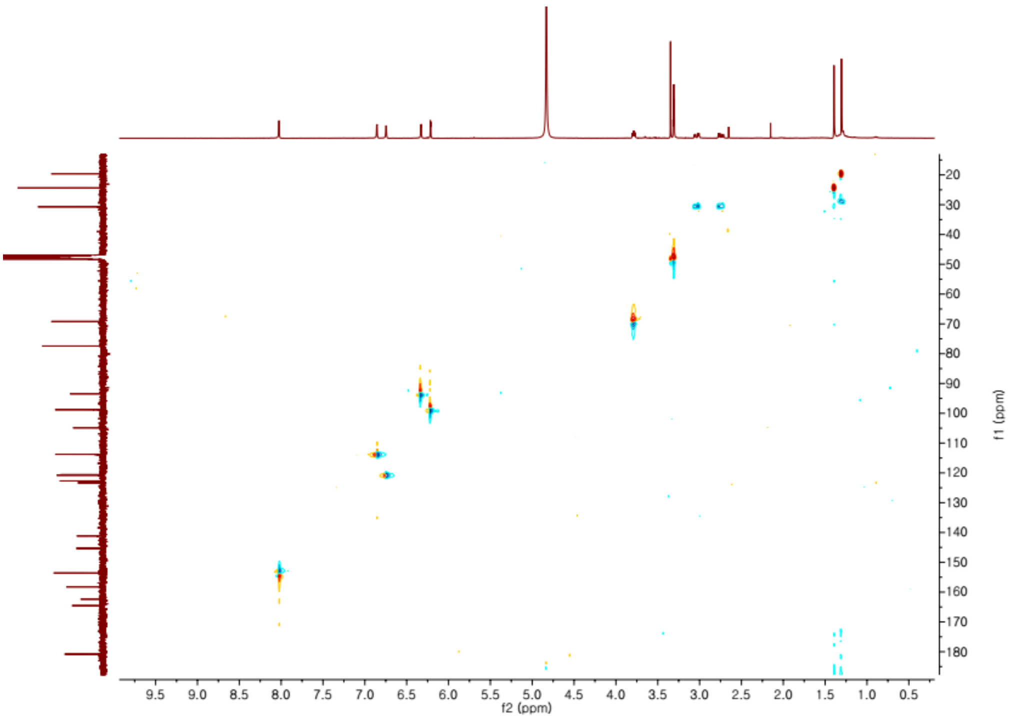

Figure S23. HSQC spectrum of **7**

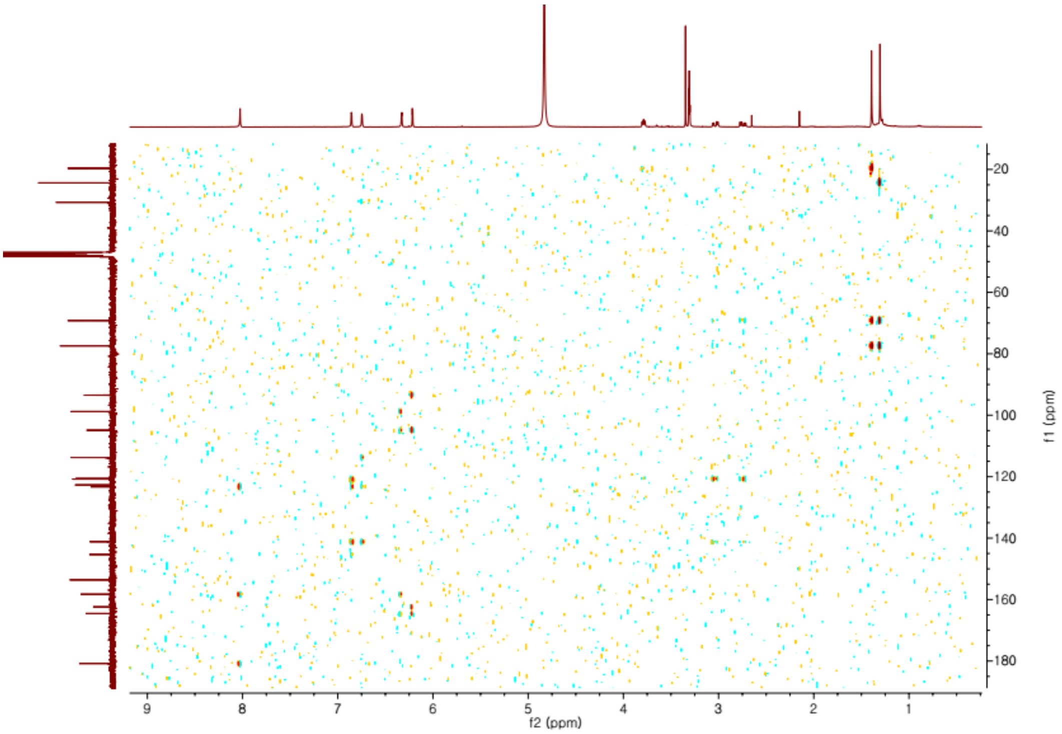

Figure S24. HMBC spectrum of **7**

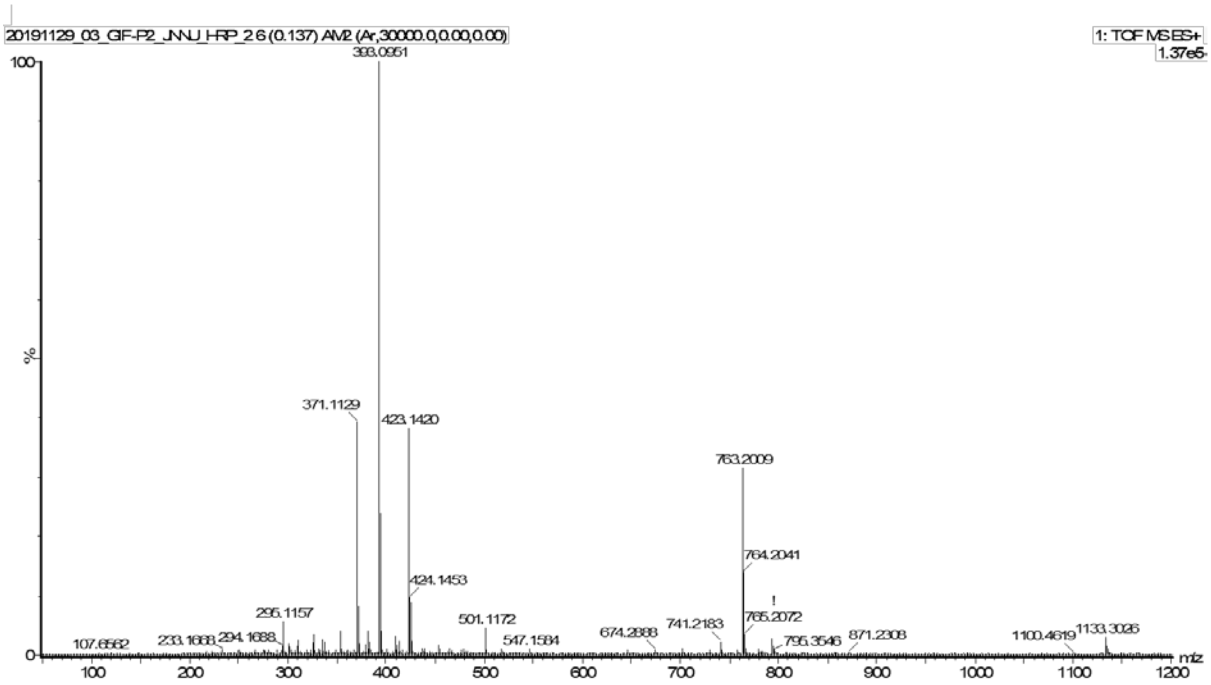

Elemental Composition Report

Single Mass Analysis
Tolerance = 5.0 PPM / DBE: min = -1.5, max = 100.0
Element prediction: Off
Number of isotope peaks used for i-FIT = 3

Monoisotopic Mass, Even Electron Ions
Elements Used:
C: 1-40 H: 1 -50 O: 1 -20 Na: 0 -1

Minimum:				-1.5				
Maximum:		100.0	5.0	100.0				
Mass	Calc. Mass	mDa	PPM	DBE	i-FIT	Norm	Conf(%)	Formula
371.1129	371.1131	-0.2	-0.5	11.5	792.4	n/a	n/a	C20 H19 O7
393.0951	393.0950	0.1	0.3	11.5	803.2	n/a	n/a	C20 H18 O7 Na

Figure S25. HRESIMS spectrum of **7**

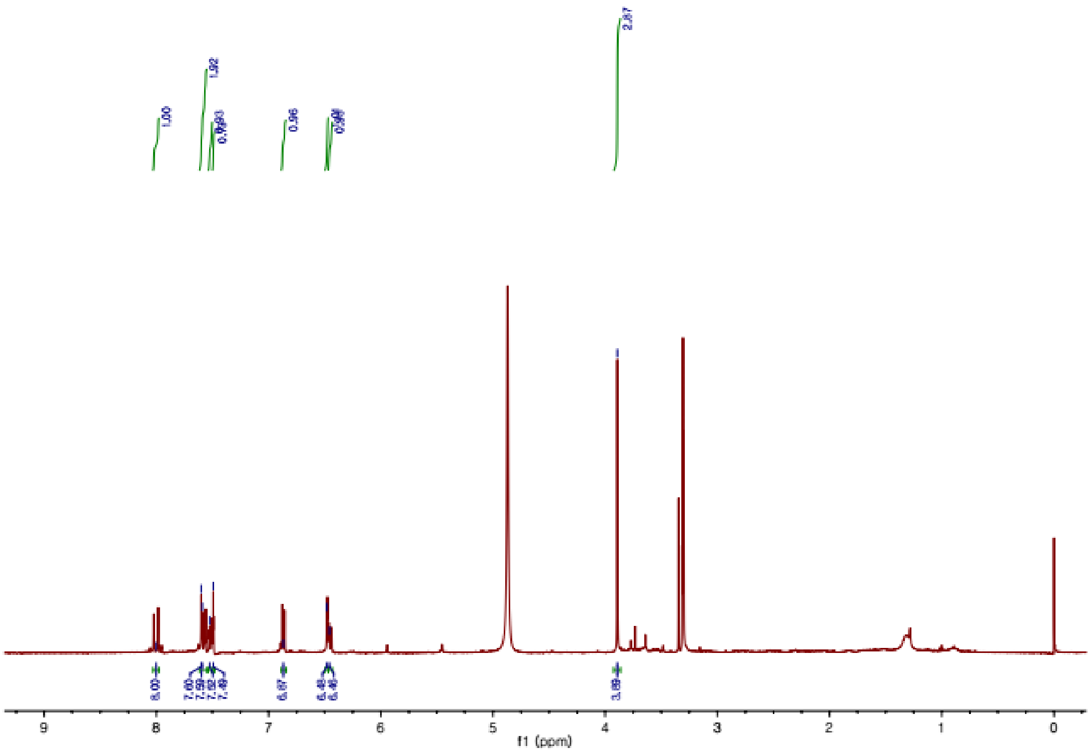

Figure S26. ^{1}H NMR spectrum of **8** (CD_3OD, 100 MHz)

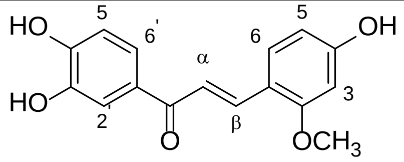 **8**	^{1}H-NMR (CD_3OD, 400 MHz, δ in ppm, *J* in Hz) δ 8.00 (1H, d, *J* = 15.7, H-α), 7.60 (1H, d, *J* = 15.7, H-β), 7.59 (1H, d, *J* = 8.4, H-6), 7.52 (1H, dd, *J* = 8.2, 2.0, H-6'), 7.49 (1H, d, *J* = 2.0, H-2'), 6.87 (1H, d, *J* = 8.2, H-5'), 6.48 (1H, d, *J* = 2.1, H-3), 6.46 (1H, dd, *J* = 8.4, 2.1, H-5), 3.89 (3H, s, OMe).

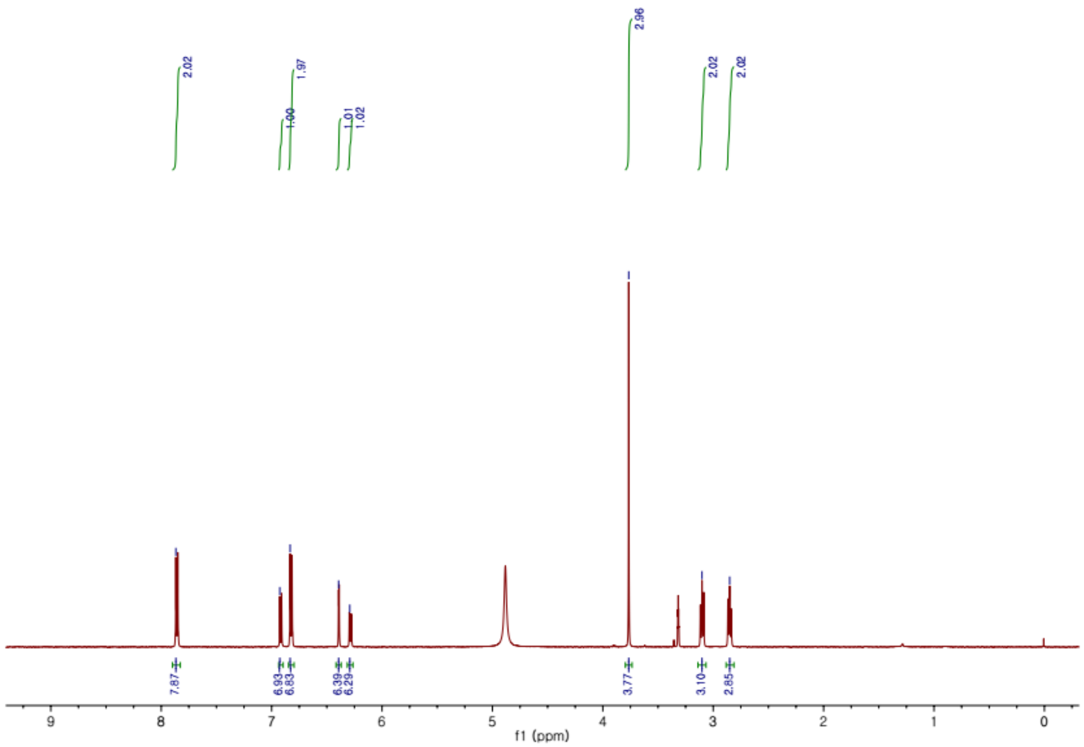

Figure S27. ^{1}H NMR spectrum of **9** (CD_3OD, 100 MHz)

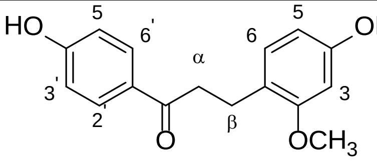 **9**	^{1}H-NMR (CD_3OD, 400 MHz, δ in ppm, *J* in Hz) δ 7.87 (2H, d, *J* = 8.8, H-2′,6′), 6.93 (1H, d, *J* = 8.2, H-6), 6.83 (2H, d, *J* = 8.8, H-3′,5′), 6.39 (1H, d, *J* = 2.2, H-3), 6.29 (1H, d, *J* = 8.2, 2.2, H-5), 3.77 (3H, s, OMe), 3.10 (2H, d, *J* = 7.4, H-α), 2.85 (2H, t, *J* = 7.4, H-β).

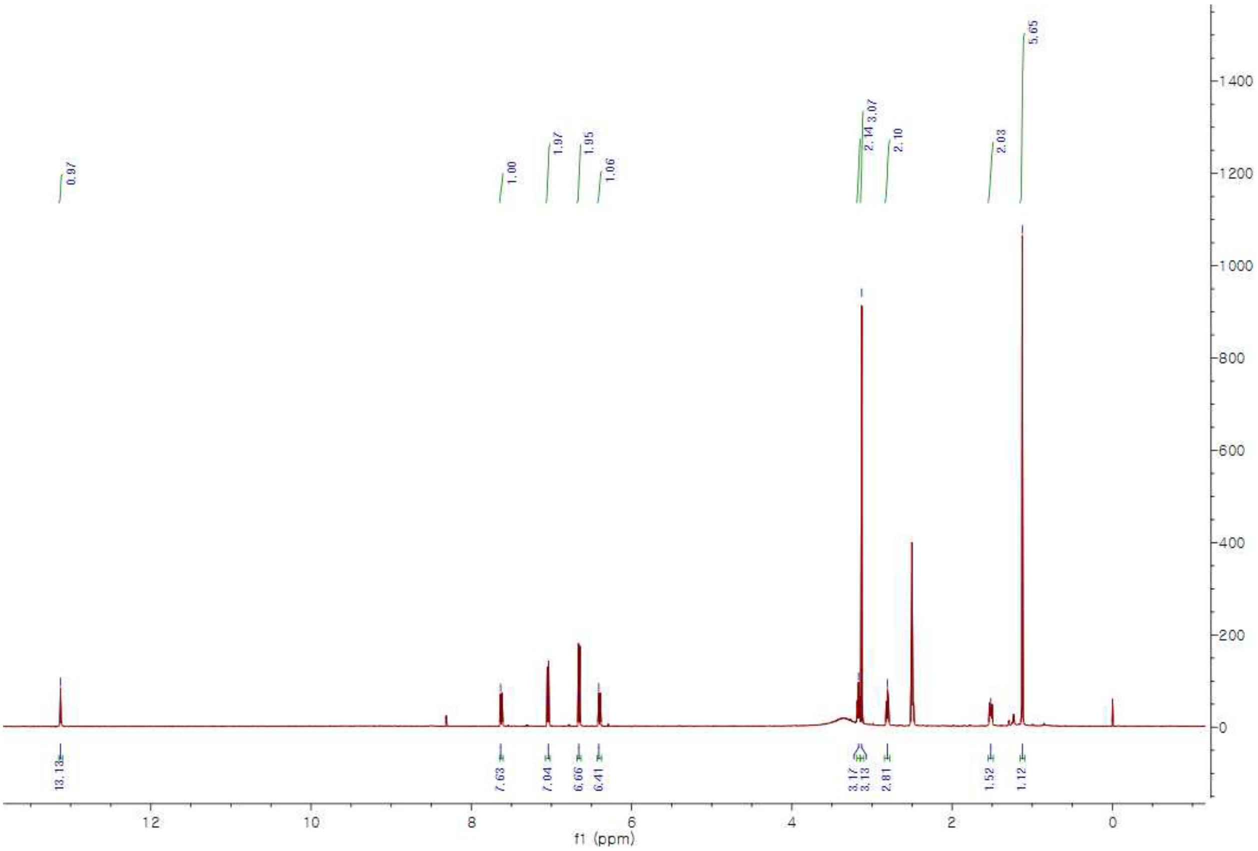

Figure S28. ^{1}H-NMR (500 MHz, DMSO-d_6) spectrum of **10**

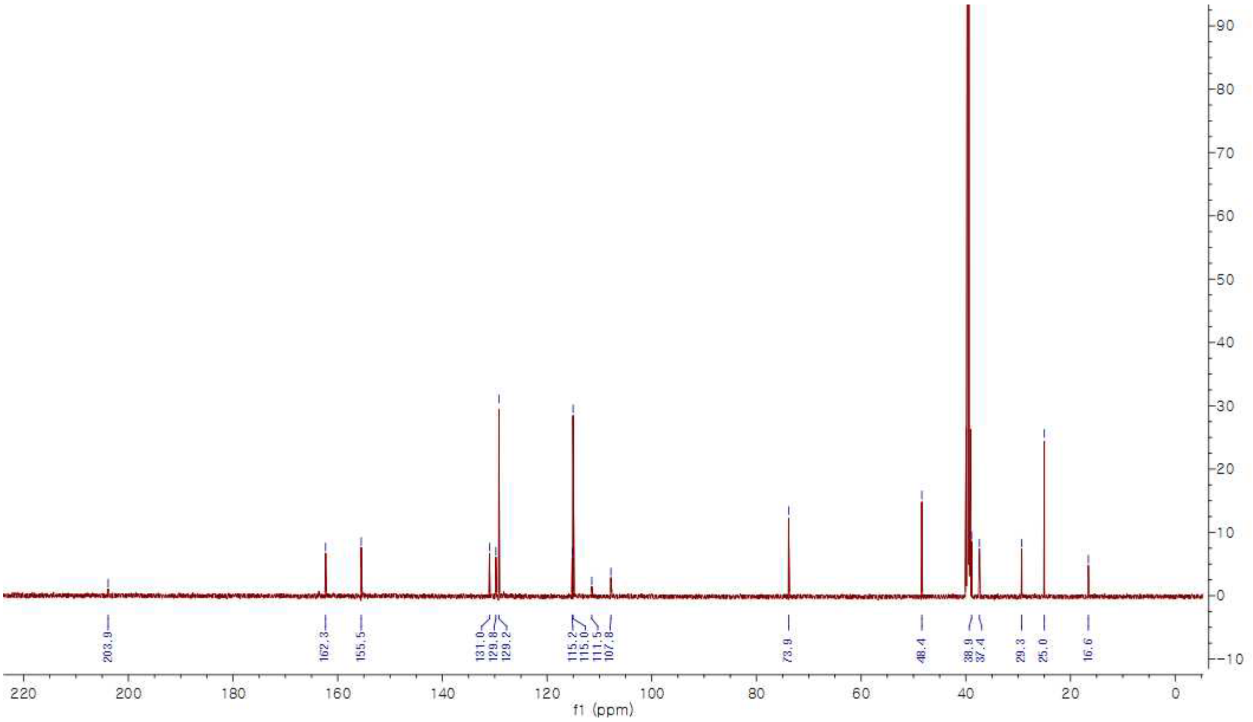

Figure S29. ^{13}C-NMR (150 MHz, DMSO-d_6) spectrum of **10**

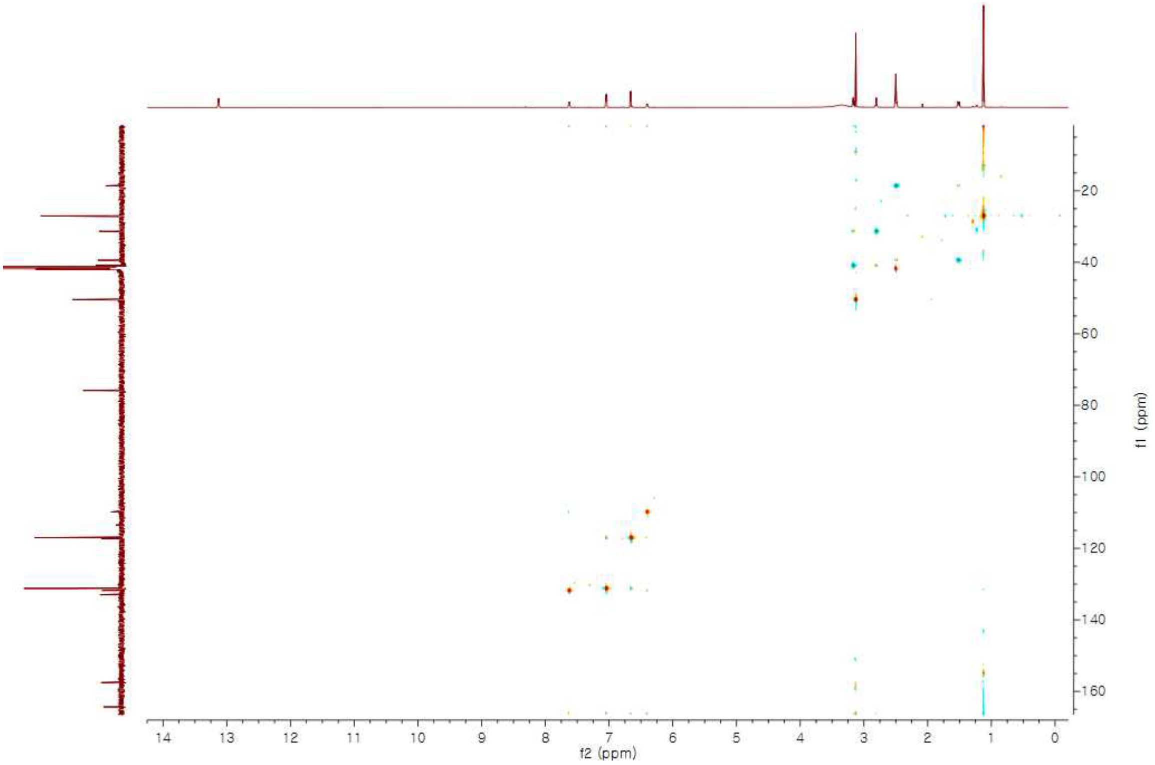

Figure S30. HSQC-spectrum of **10**

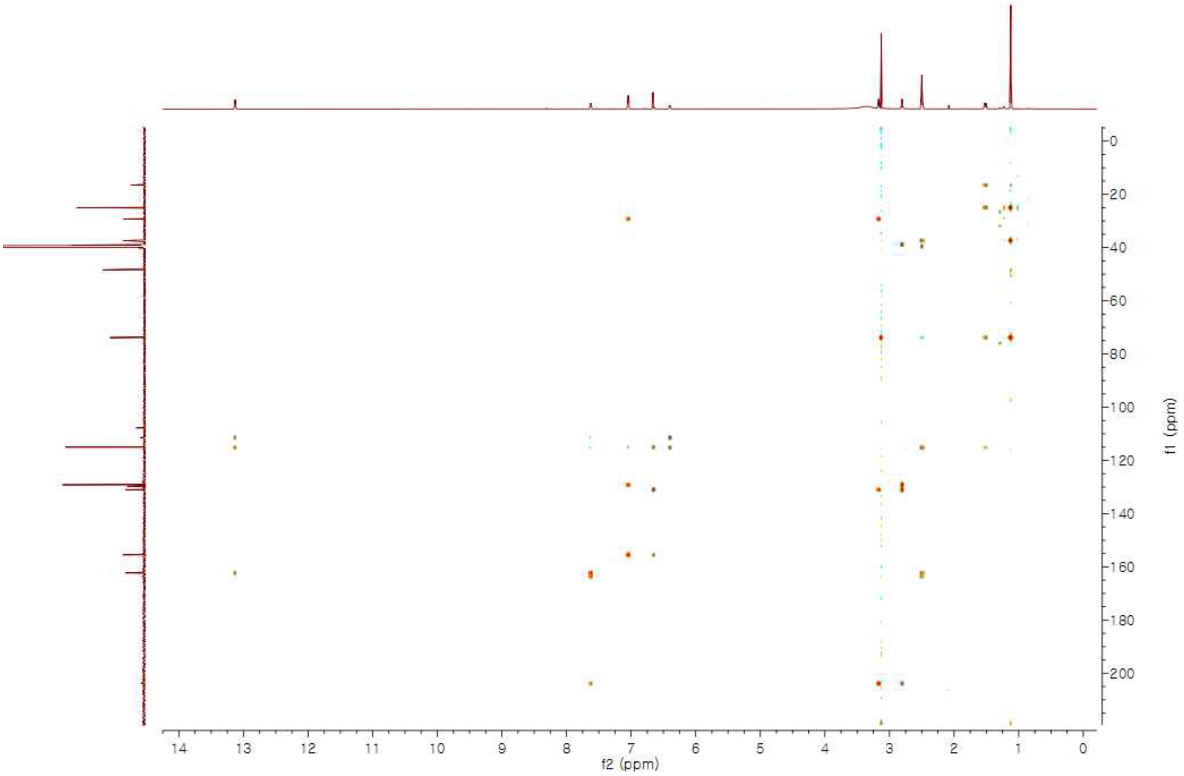

Figure S31. HMBC spectrum of **10**

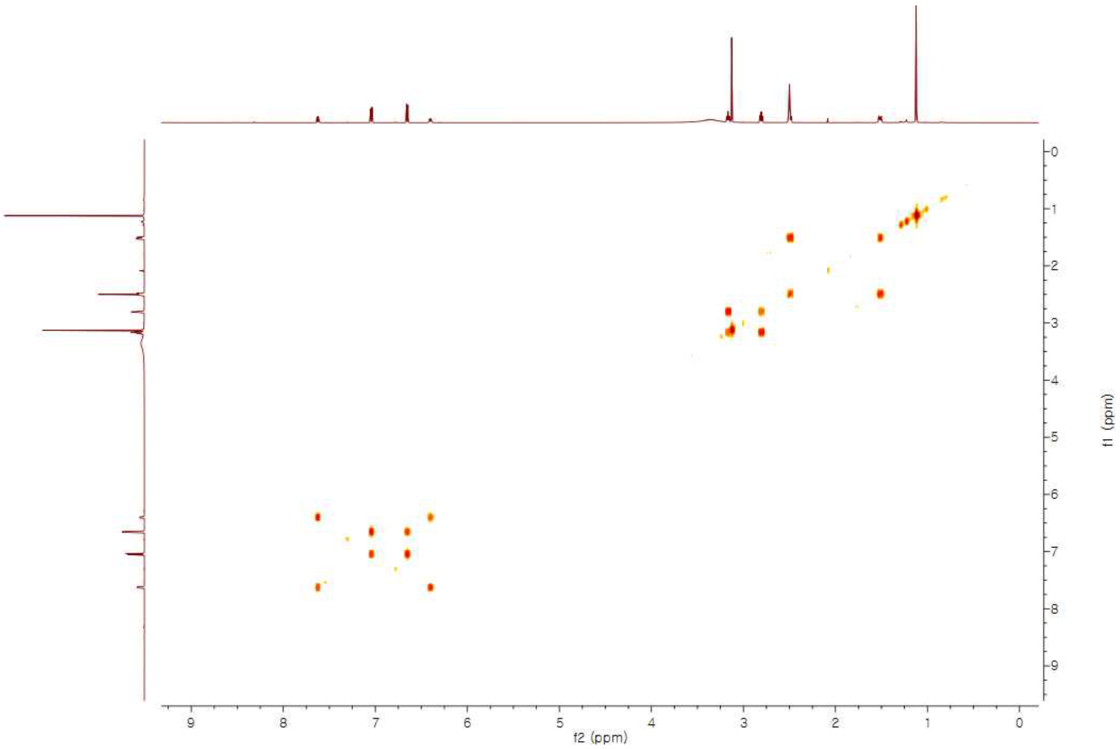

Figure S32. COSY spectrum of **10**

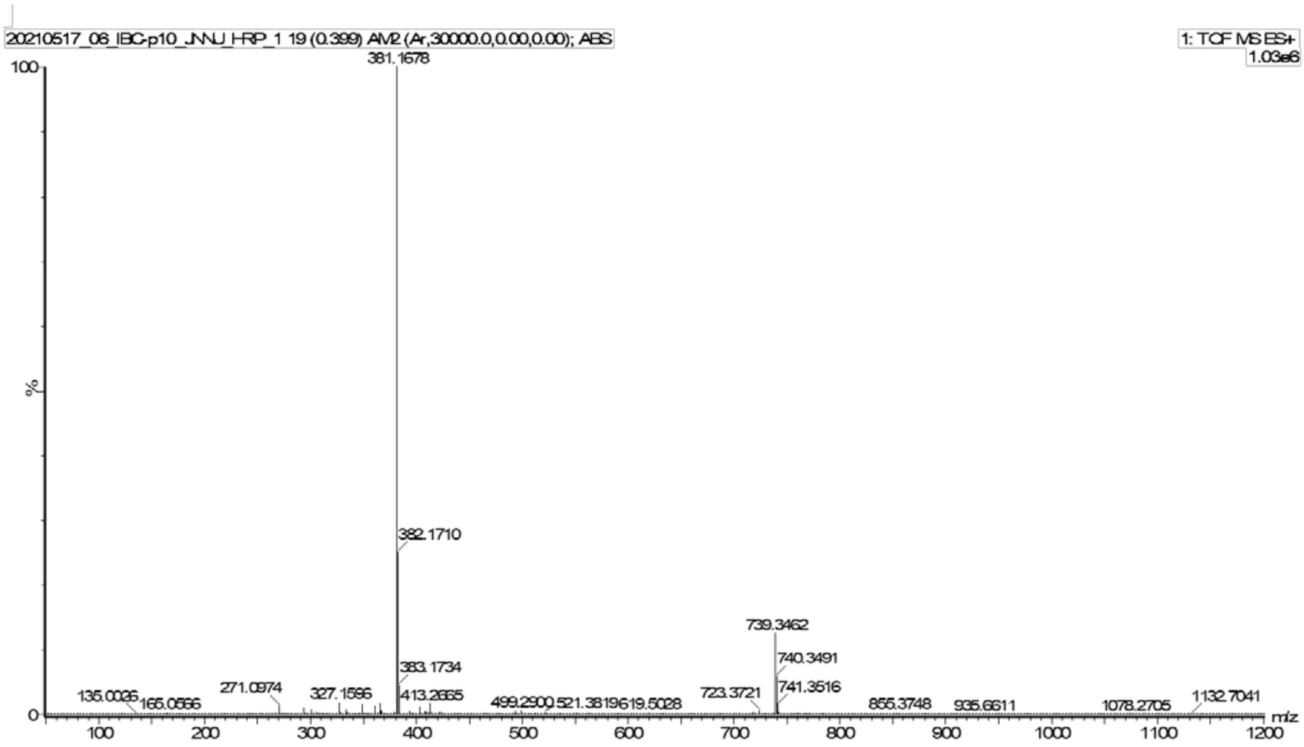

Elemental Composition Report

Single Mass Analysis
Tolerance = 5.0 PPM / DBE: min = -1.5, max = 100.0
Element prediction: Off
Number of isotope peaks used for i-FIT = 3

Monoisotopic Mass, Even Electron Ions
10 formula(e) evaluated with 1 results within limits (all results (up to 1000) for each mass)
Elements Used:
C: 0-22 H: 0 -30 O: 0 -5 Na : 0-1

Minimum:				-1.5				
Maximum:		100.0	5.0	100.0				
Mass	Calc. Mass	mDa	PPM	DBE	i-FIT	Norm	Conf(%)	Formula
381.1678	381.1678	0.0	0.0	8.5	1035.6	n/a	n/a	C21 H26 O5 Na

Figure S33. HRESIMS spectrum of **10**

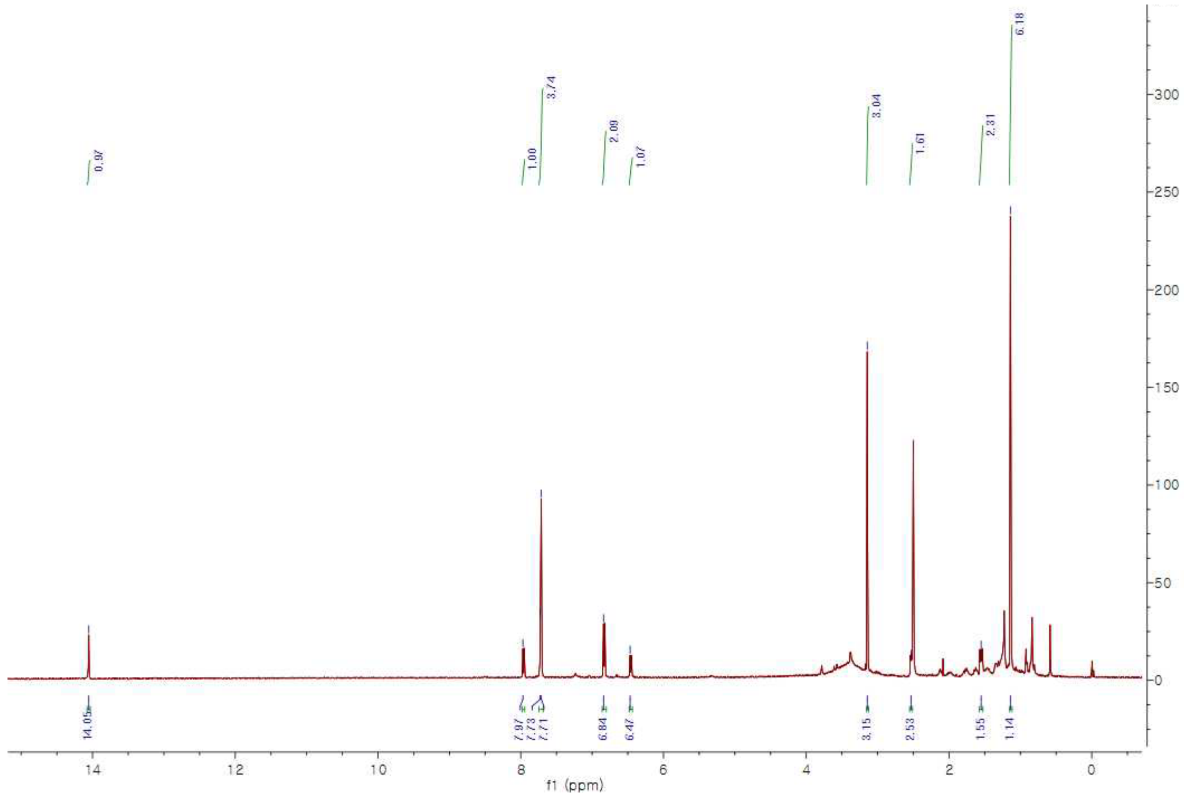

Figure S34. ^{1}H-NMR (500 MHz, DMSO-d_6) spectrum of **11**

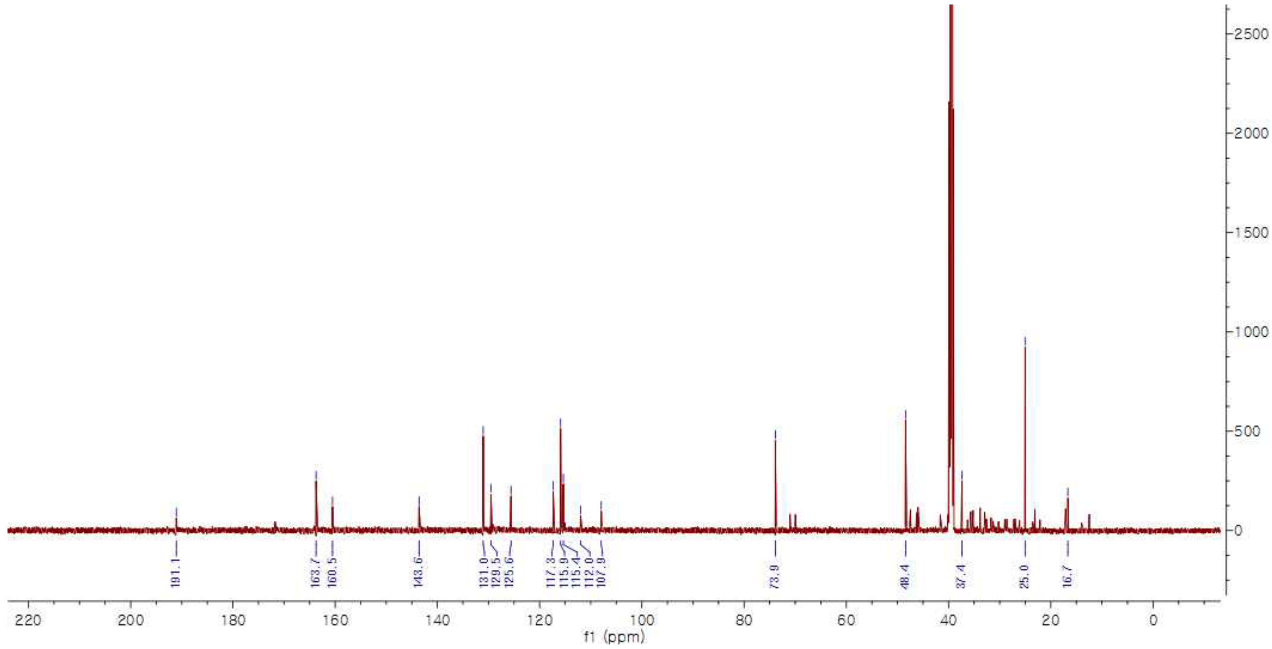

Figure S35. ^{13}C-NMR (150 MHz, DMSO-d_6) spectrum of **11**

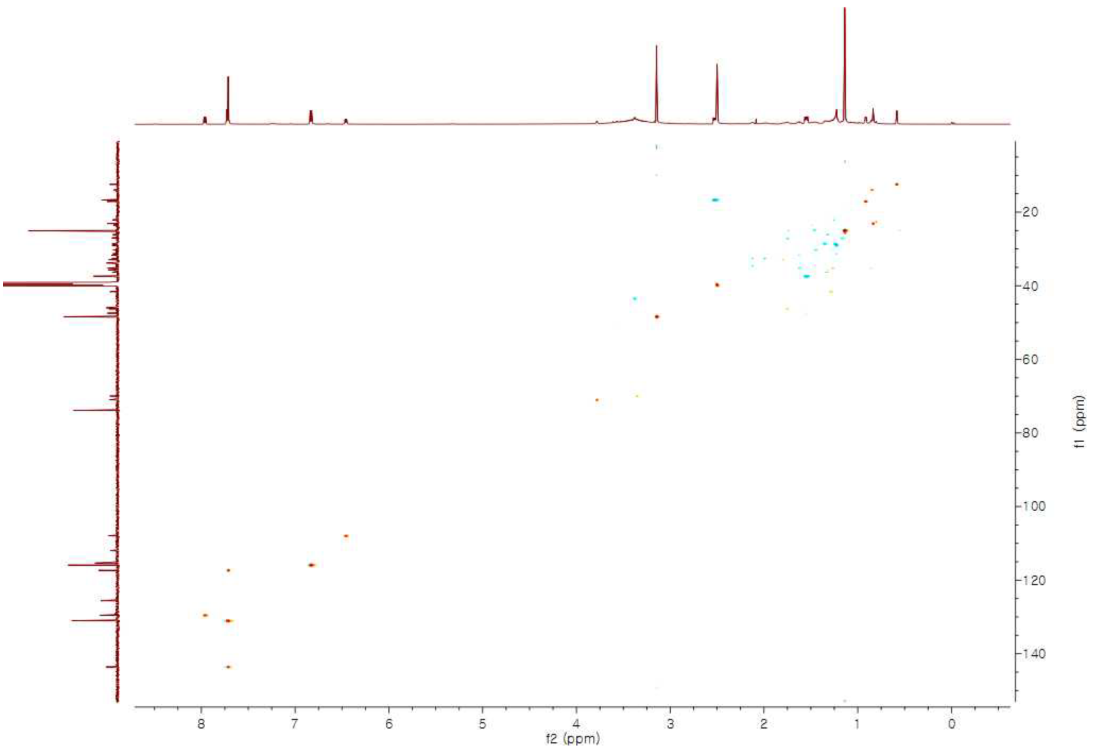

Figure S36. HSQC spectrum of **11**

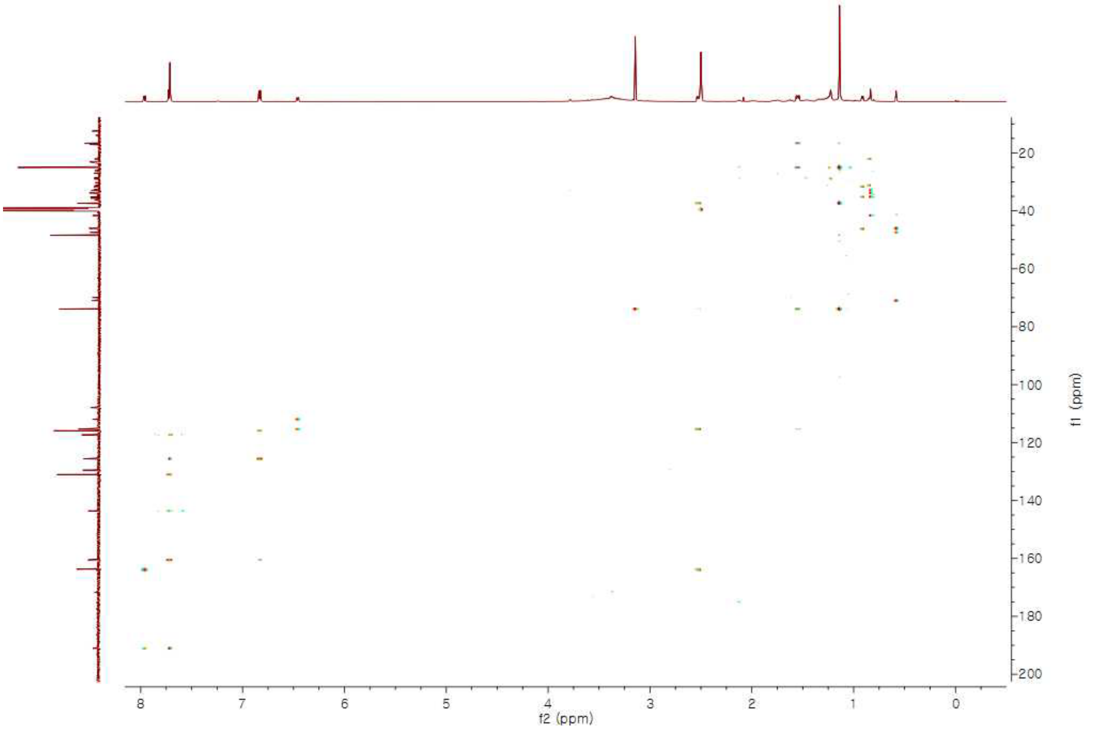

Figure S37. HMBC spectrum of **11**

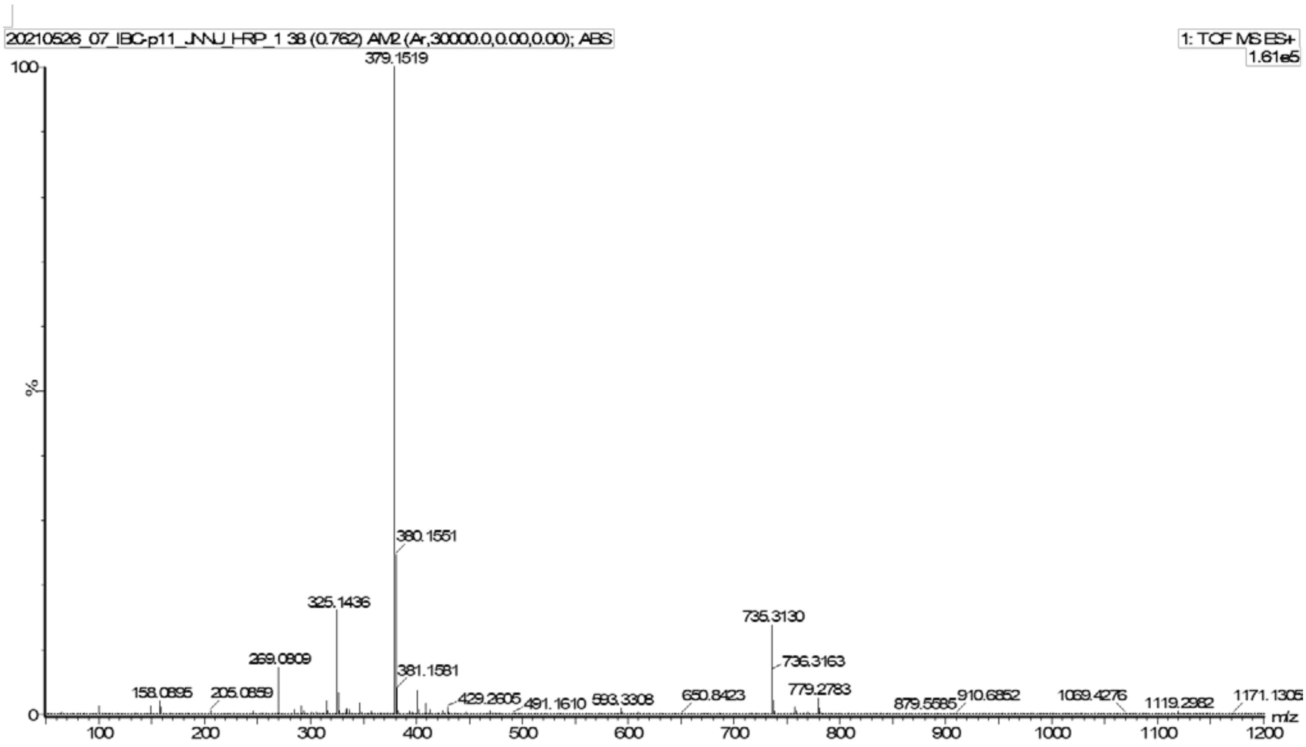

Elemental Composition Report

Single Mass Analysis
Tolerance = 5.0 PPM / DBE: min = -1.5, max = 100.0
Element prediction: Off
Number of isotope peaks used for i-FIT = 3

Monoisotopic Mass, Even Electron Ions
92 formula(e) evaluated with 1 results within limits (all results (up to 1000) for each mass)
Elements Used:
C: 0-29 H: 0 -45 O: 0 -6 Na: 0 -1 Gd: 0 -1

Minimum:				-1.5				
Maximum:		100.0	5.0	100.0				
Mass	Calc. Mass	mDa	PPM	DBE	i-FIT	Norm	Conf(%)	Formula
379.1519	379.1521	-0.2	-0.5	9.5	806.8	n/a	n/a	C21 H24 O5 Na

Figure S38. HRESIMS spectrum of **11**

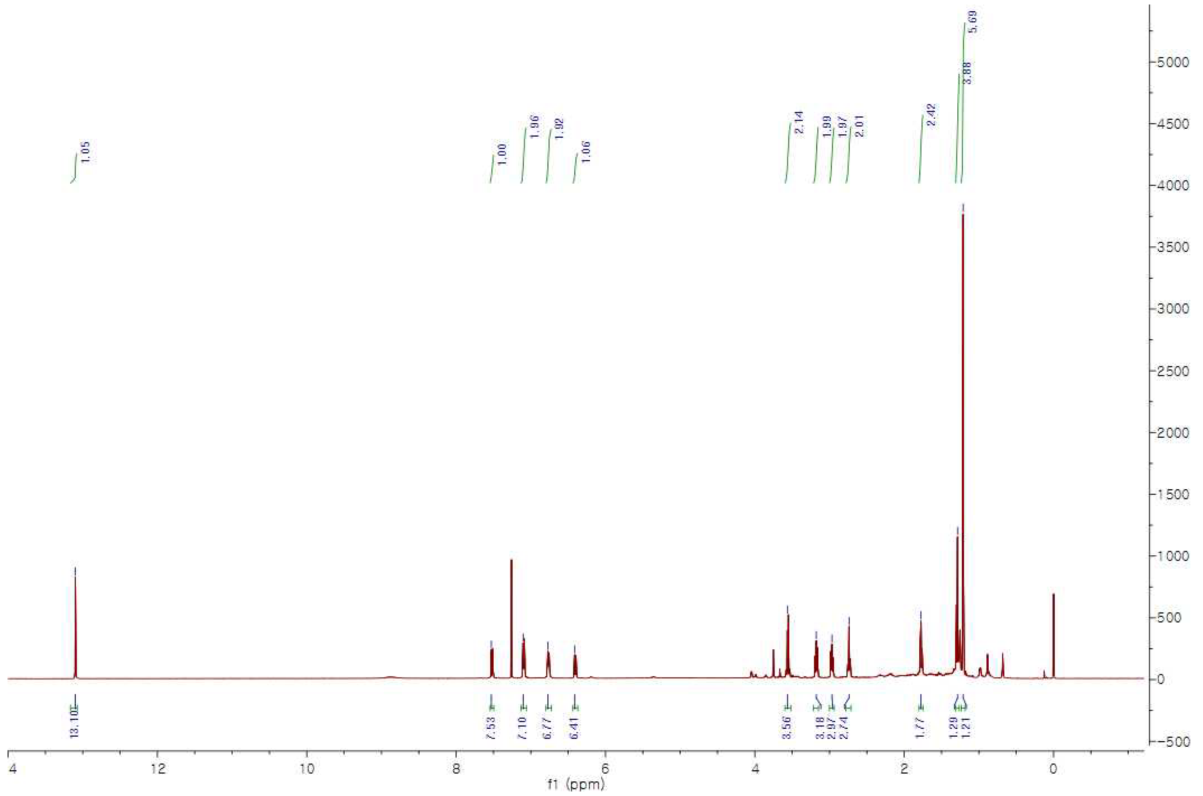

Figure S39. ^{1}H-NMR (500 MHz, $CDCl_3$) spectrum of **12**

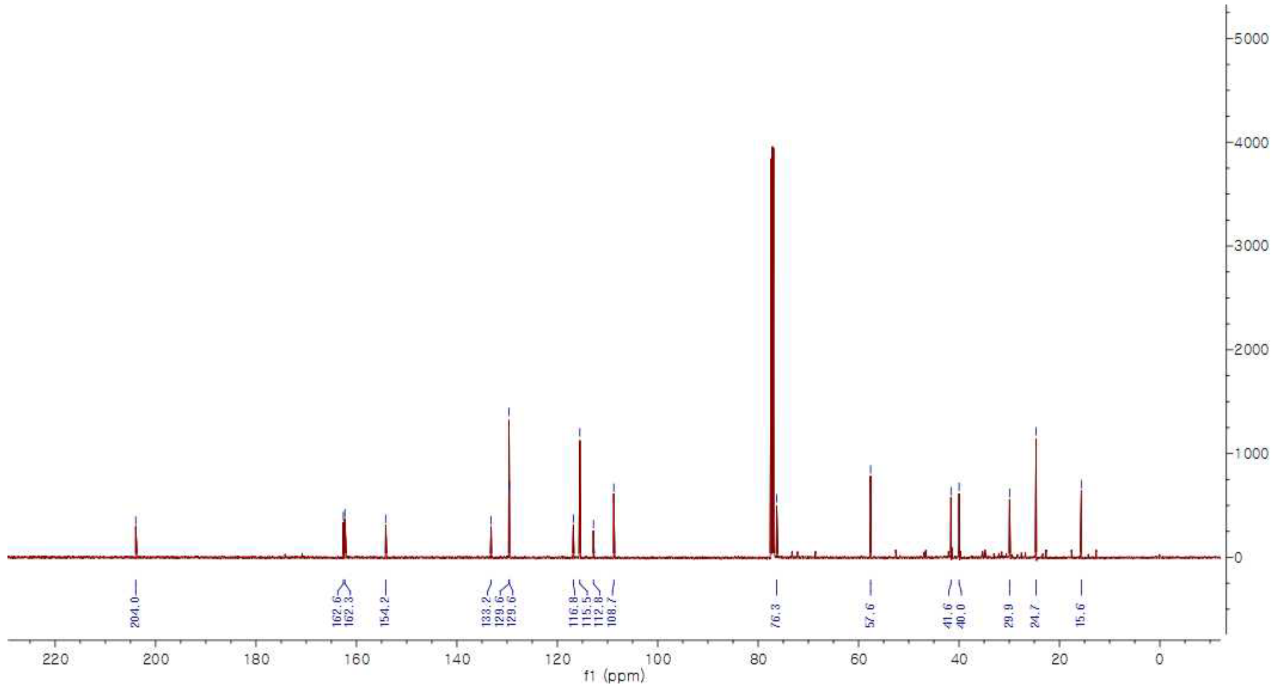

Figure S40. ^{13}C-NMR (150 MHz, $CDCl_3$) spectrum of **12**

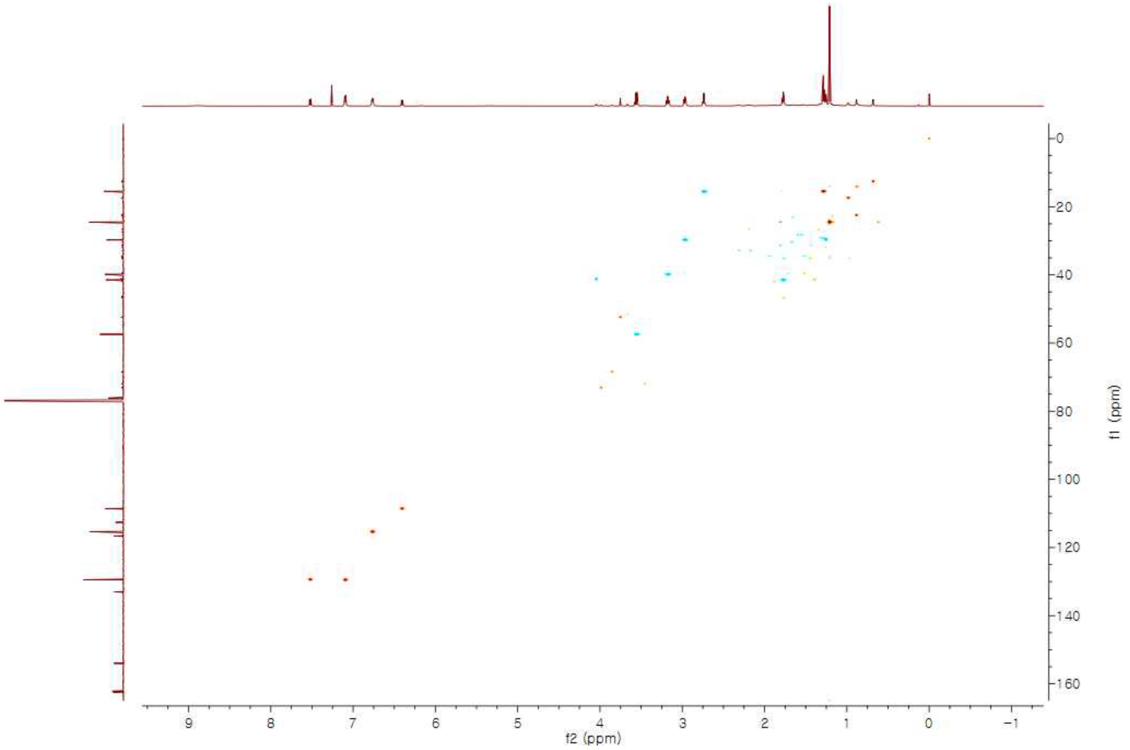

Figure S41. HSQC spectrum of **12**

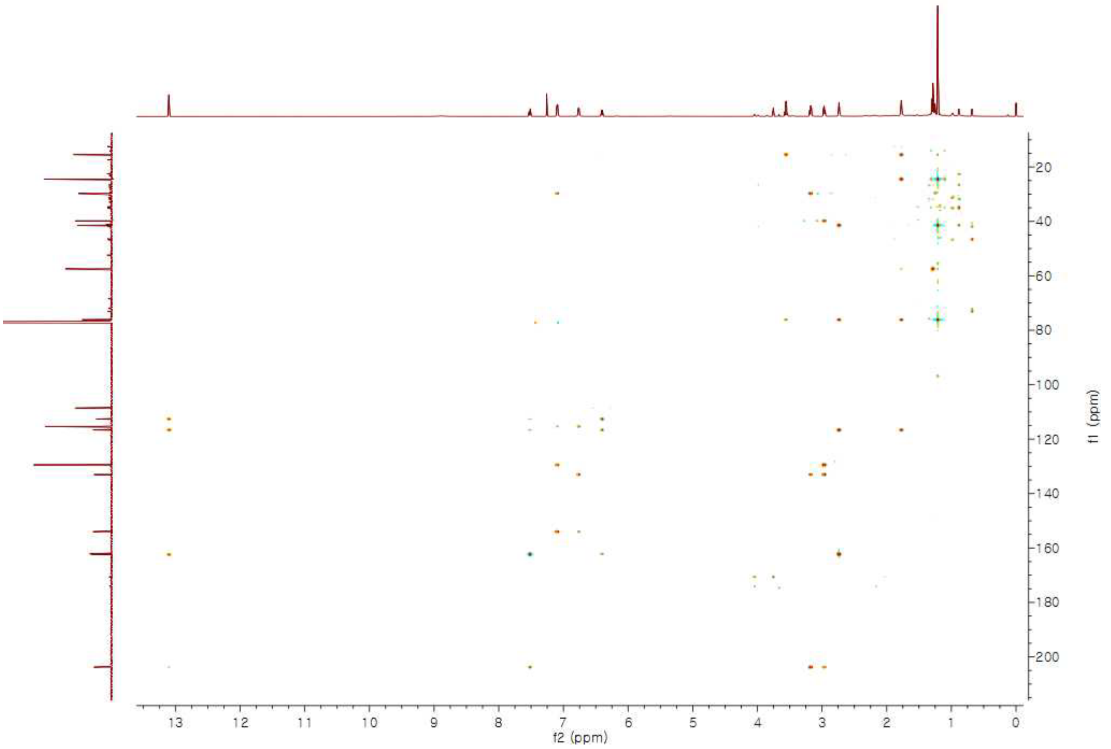

Figure S42. HMBC spectrum of **12**

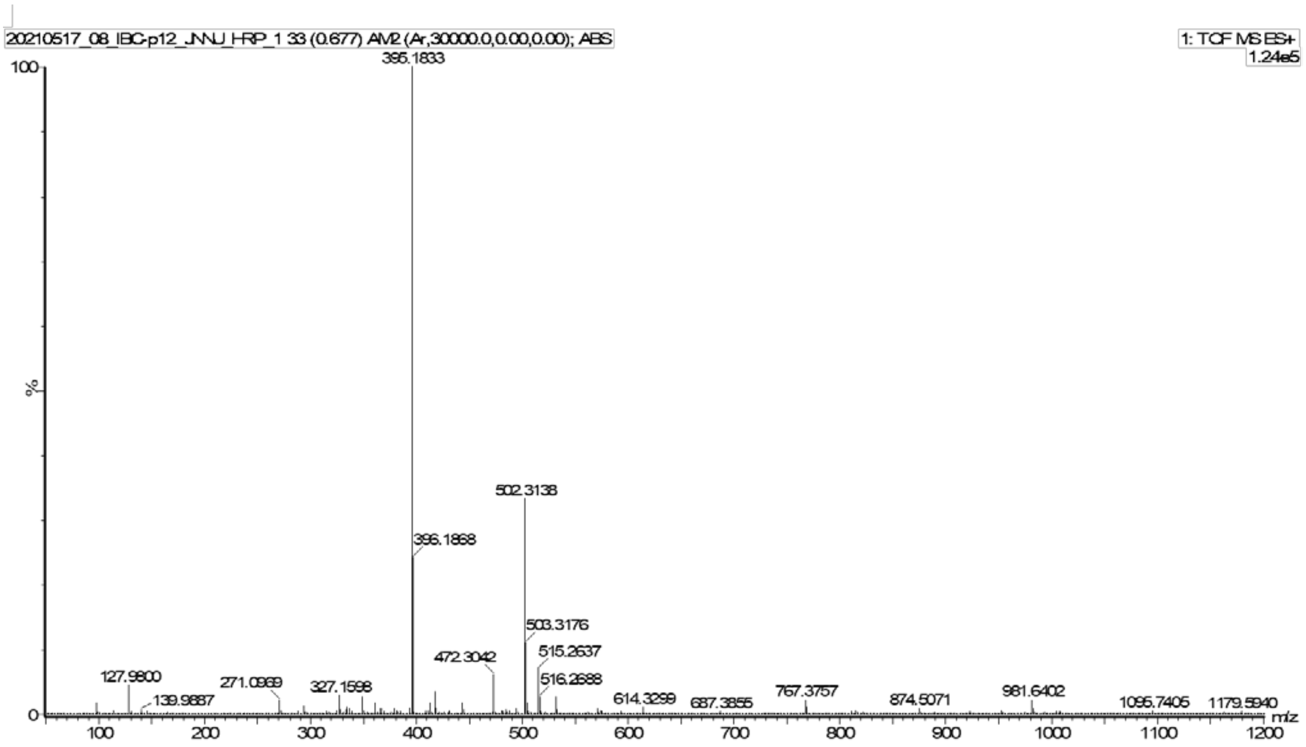

Elemental Composition Report

Single Mass Analysis
Tolerance = 5.0 PPM / DBE: min = -1.5, max = 100.0
Element prediction: Off
Number of isotope peaks used for i-FIT = 3

Monoisotopic Mass, Even Electron Ions
10 formula(e) evaluated with 1 results within limits (all results (up to 1000) for each mass)
Elements Used:
C: 0-22 H: 0 -30 O: 0 -5 Na: 0 -1

Minimum:				-1.5				
Maximum:		100.0	5.0	100.0				
Mass	Calc. Mass	mDa	PPM	DBE	i-FIT	Norm	Conf(%)	Formula
395.1833	395.1834	-0.1	-0.3	8.5	746.4	n/a	n/a	C22 H28 O5 Na

Figure S43. HRESIMS spectrum of **12**

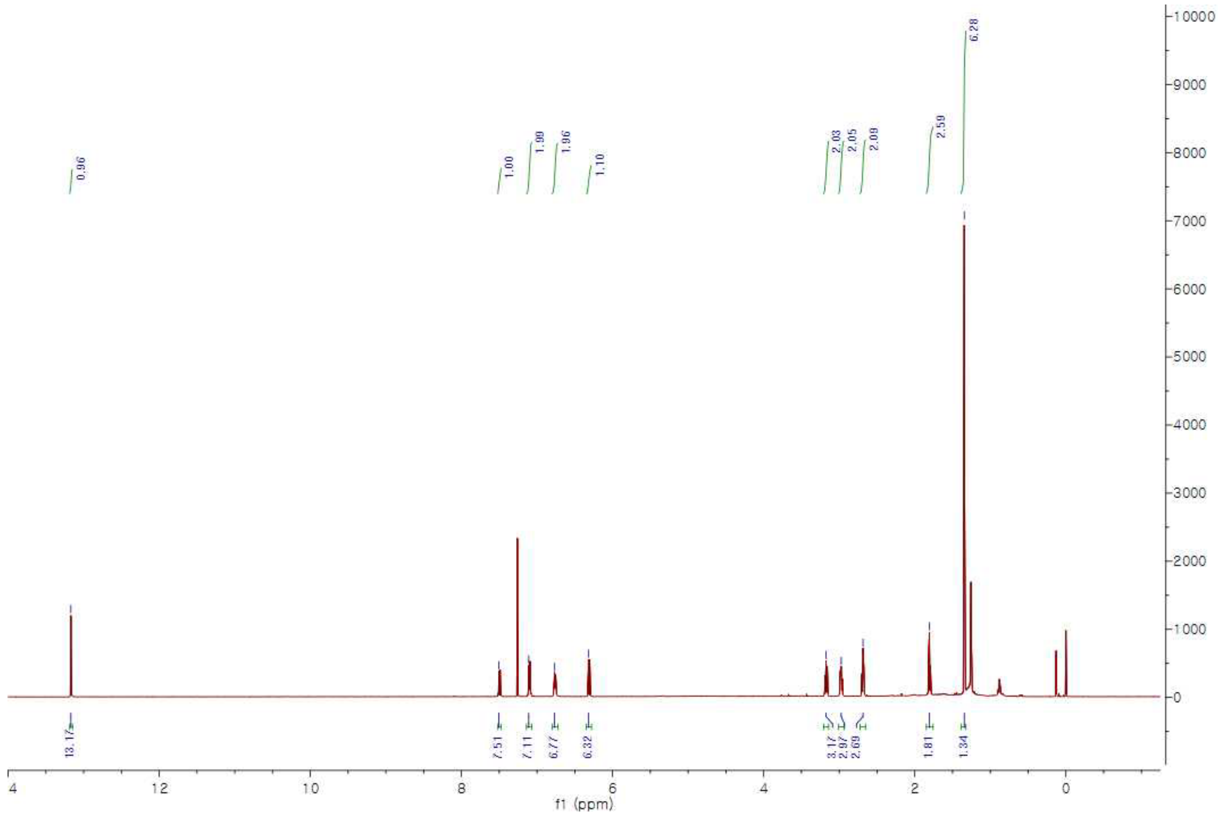

Figure S44. ^{1}H-NMR (500 MHz, $CDCl_3$) spectrum of **13**

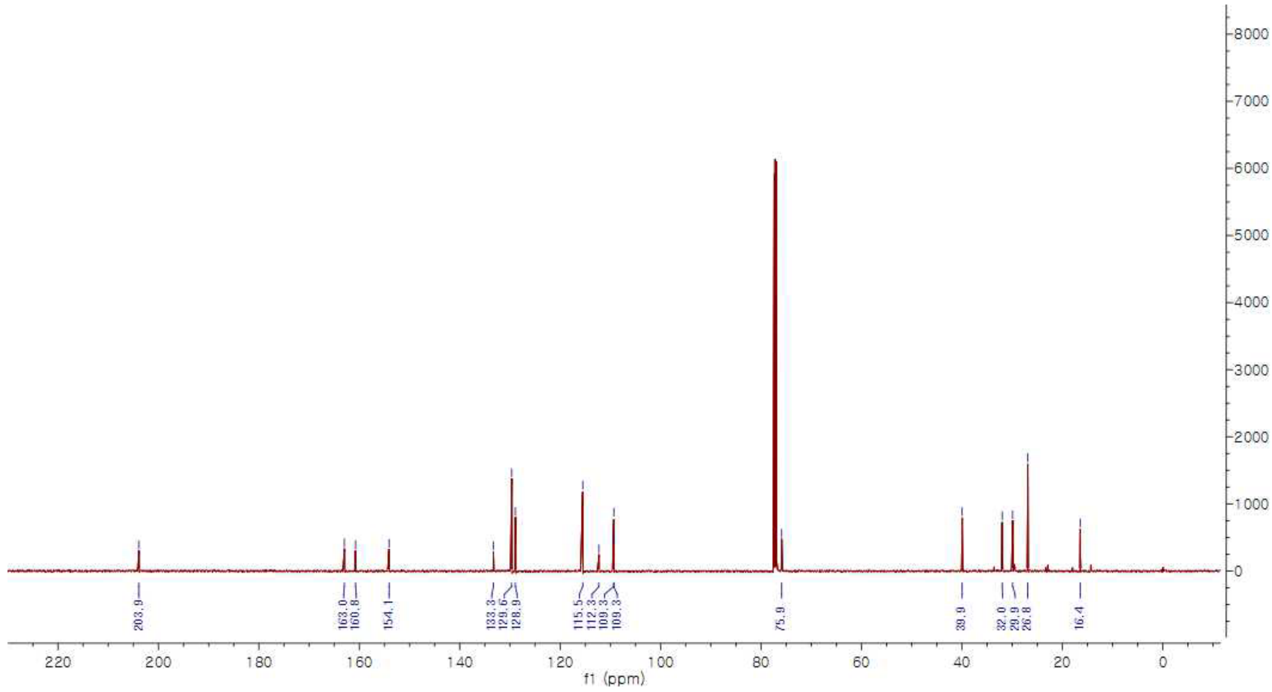

Figure S45. ^{13}C NMR (150 MHz, $CDCl_3$) spectrum of **13**

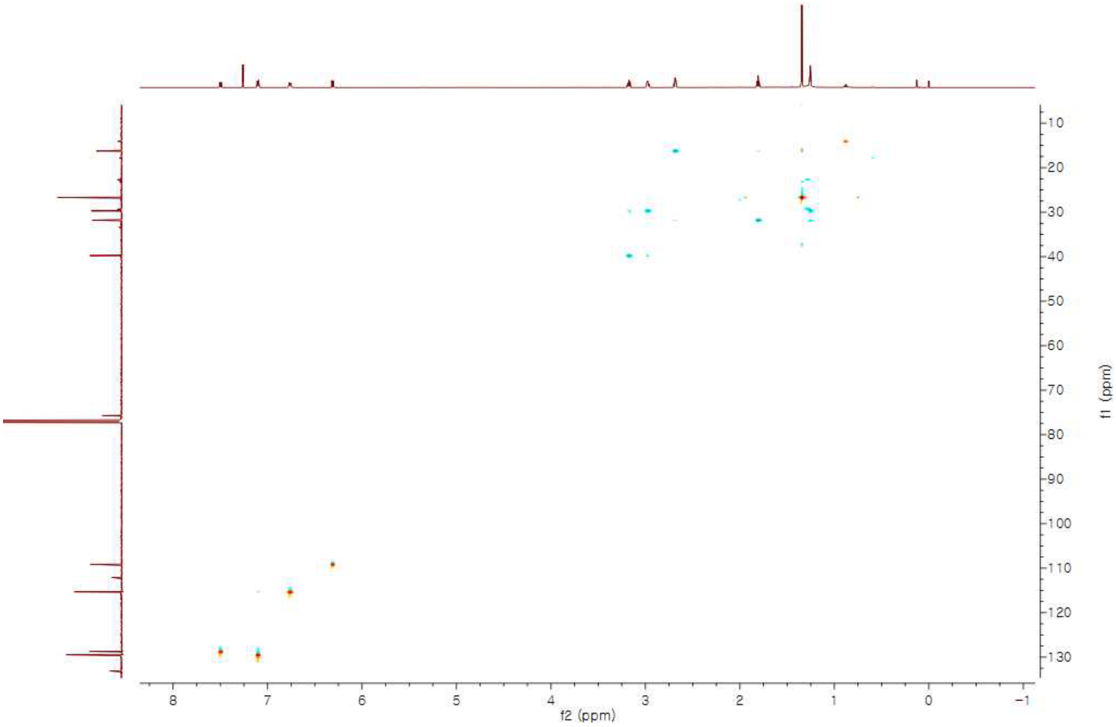

Figure S46. HSQC spectrum of **13**

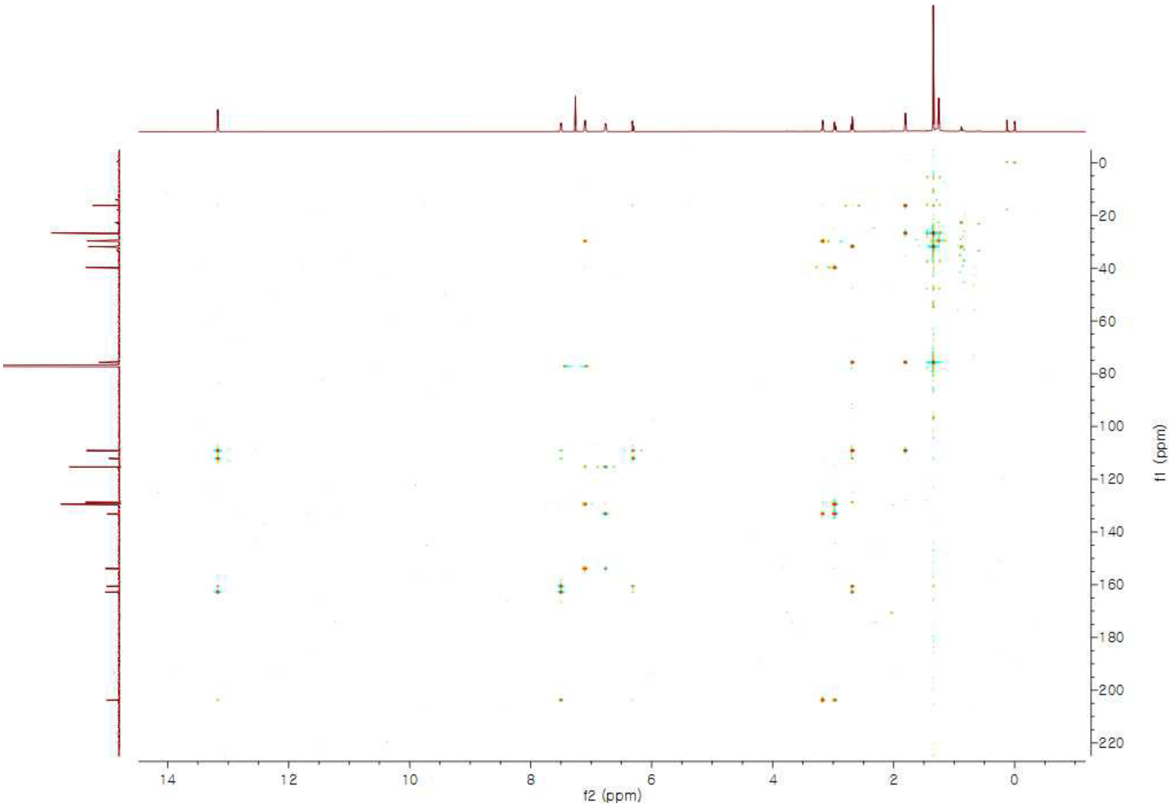

Figure S47. HMBC spectrum of **13**

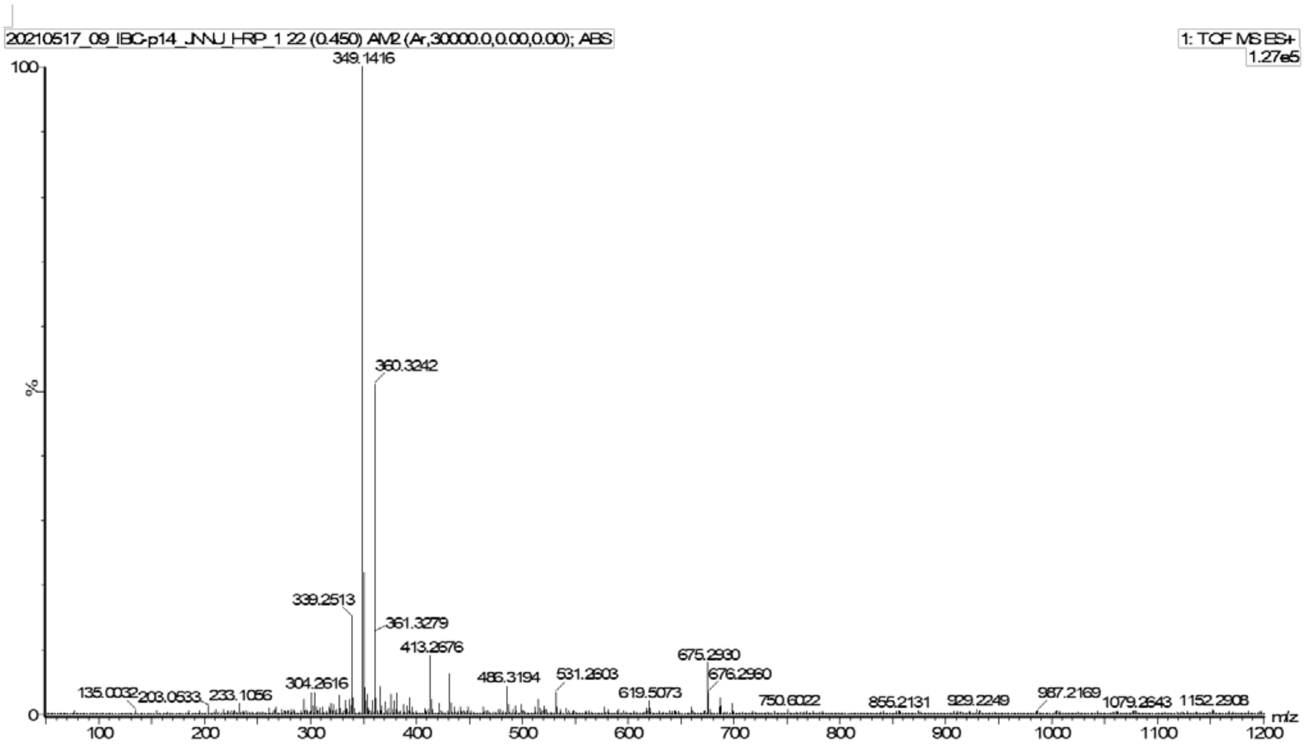

Elemental Composition Report

Single Mass Analysis
Tolerance = 5.0 PPM / DBE: min = -1.5, max = 100.0
Element prediction: Off
Number of isotope peaks used for i-FIT = 3

Monoisotopic Mass, Even Electron Ions
15 formula(e) evaluated with 1 results within limits (all results (up to 1000) for each mass)
Elements Used:
C: 0-22 H: 0 -30 O: 0 -5 Na: 0 -1

Minimum:				-1.5				
Maximum:		100.0	5.0	100.0				
Mass	Calc. Mass	mDa	PPM	DBE	i-FIT	Norm	Conf(%)	Formula
349.1416	349.1416	0.0	0.0	9.5	829.6	n/a	n/a	C20 H22 O4 Na

Figure S48. HRESIMS spectrum of **13**

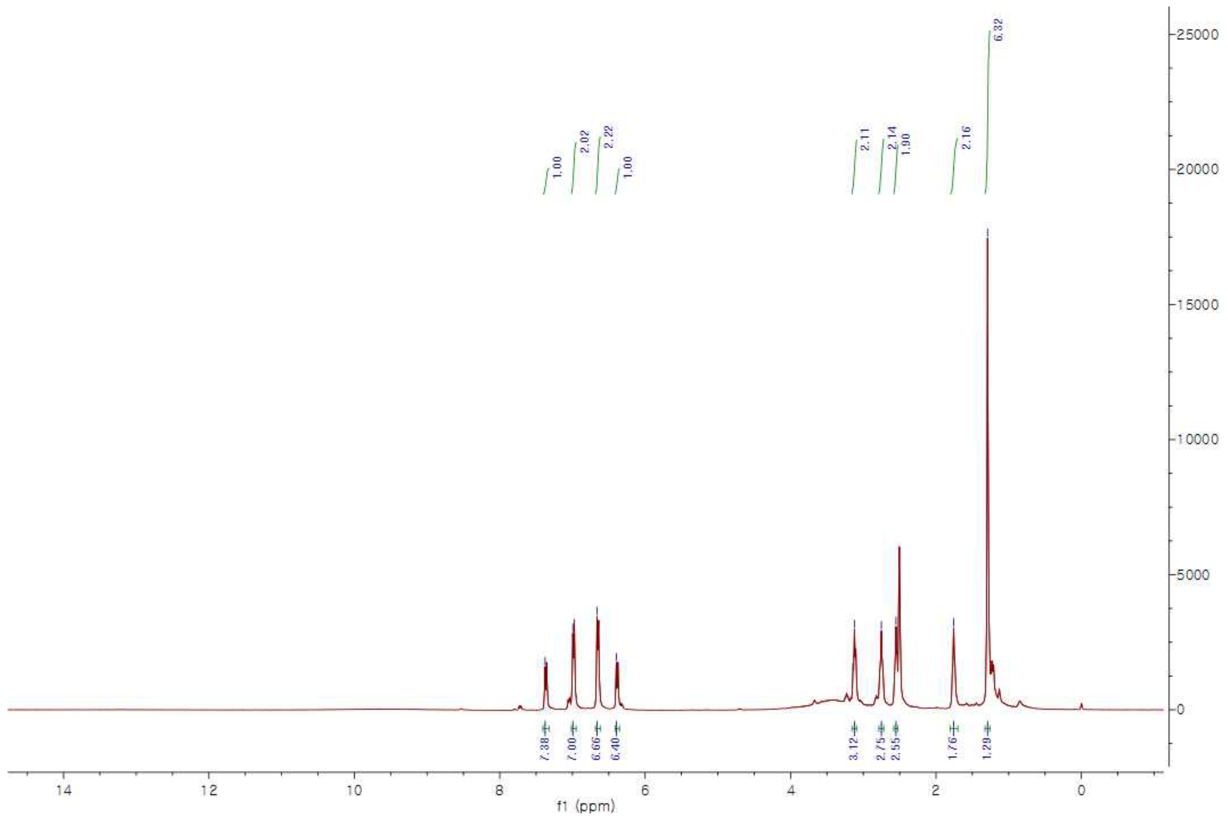

Figure S49. ^{1}H-NMR (400 MHz, DMSO-d_6) spectrum of **14**

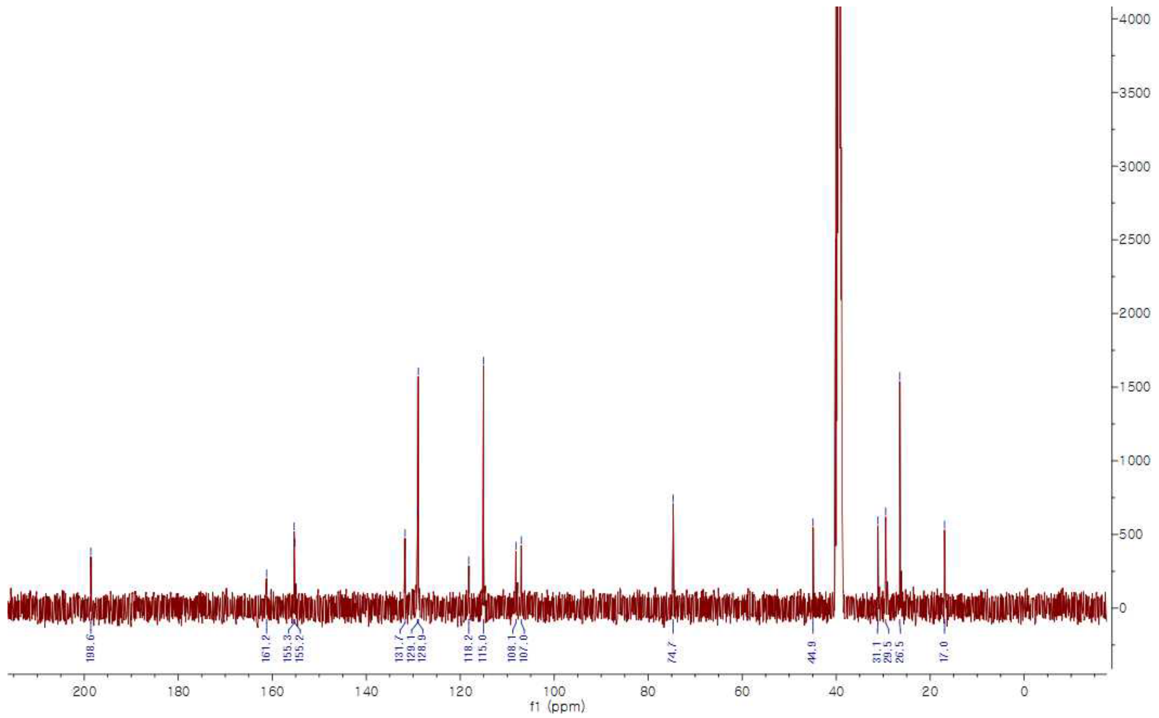

Figure S50. ^{13}C-NMR (100 MHz, DMSO-d_6) spectrum of **14**

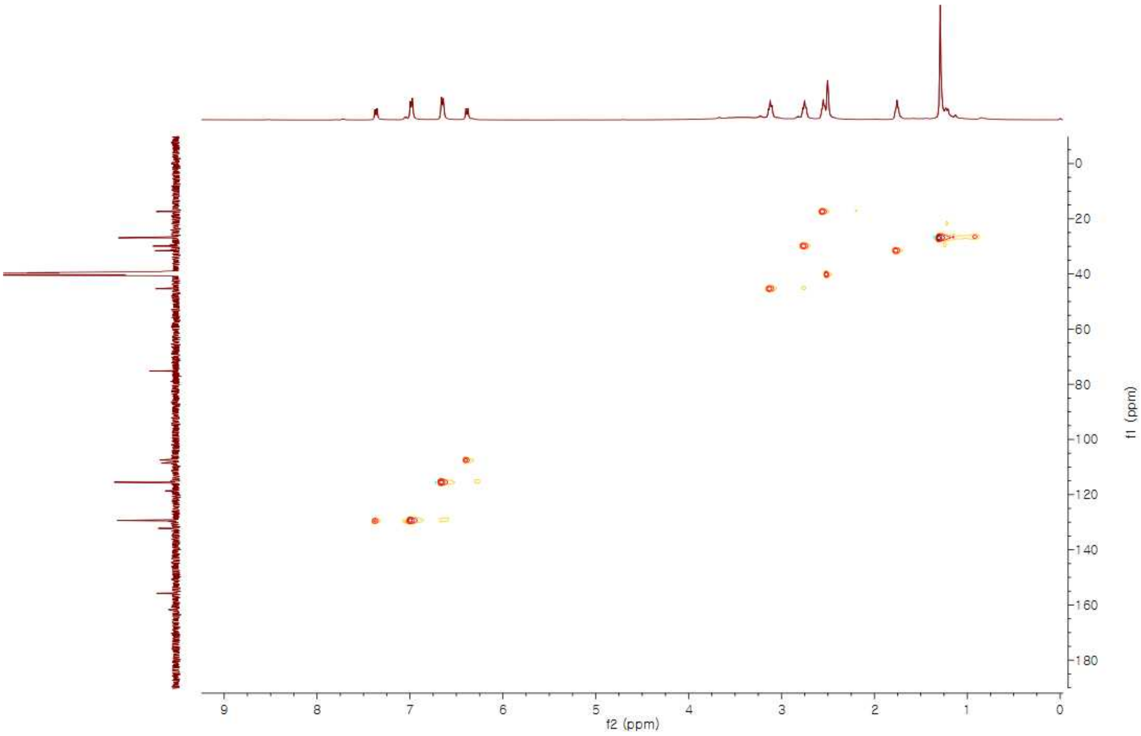

Figure S51. HSQC spectrum of **14**

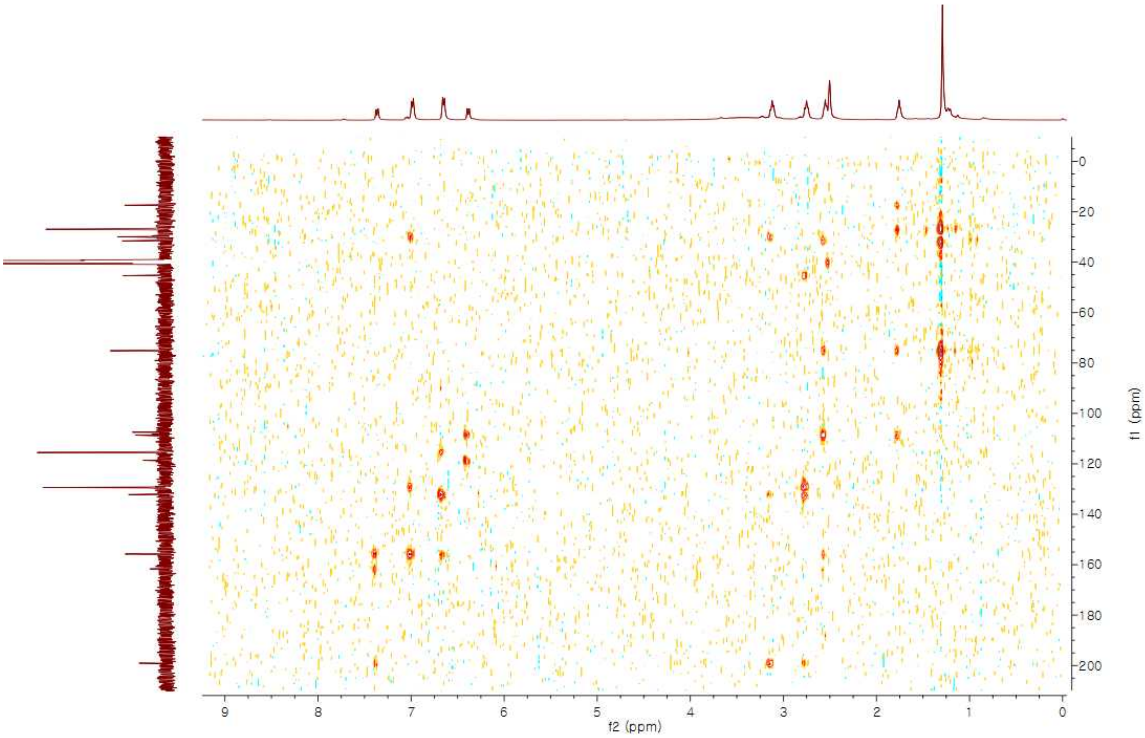

Figure S52. HMBC spectrum of **14**

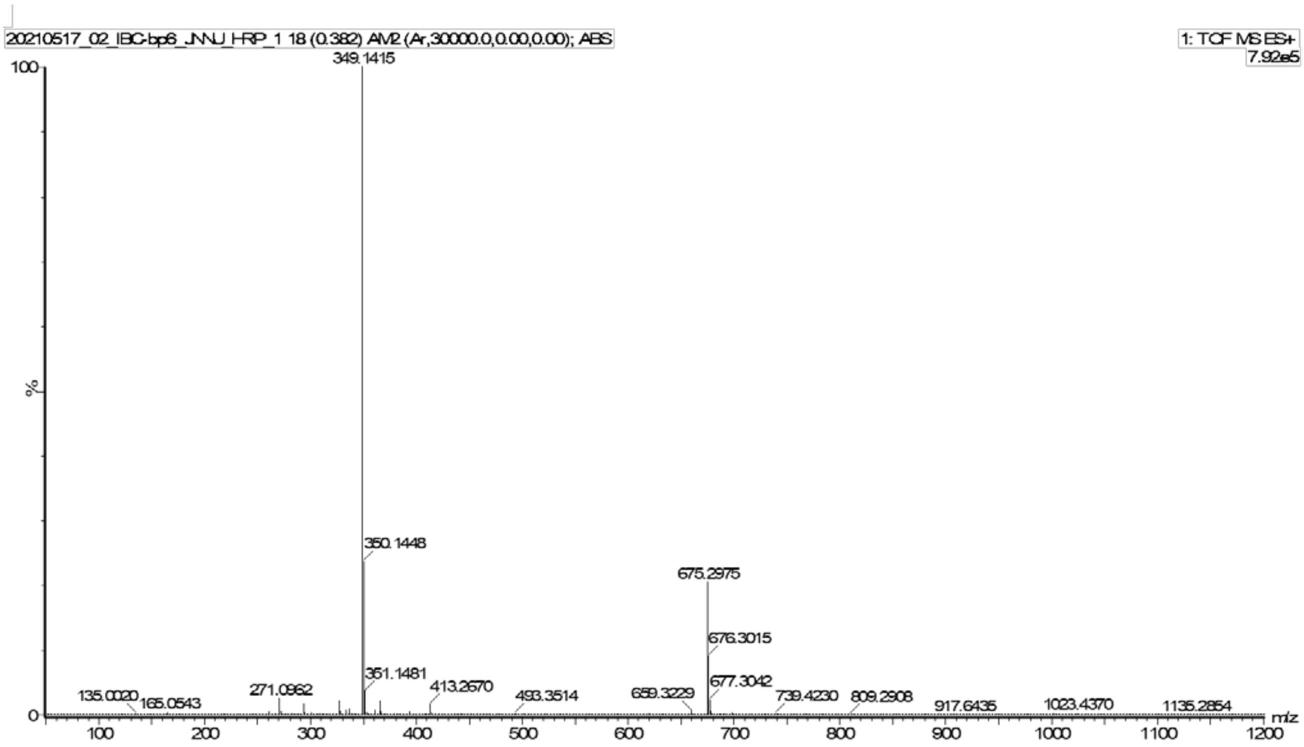

Elemental Composition Report

Single Mass Analysis
Tolerance = 5.0 PPM / DBE: min = -1.5, max = 100.0
Element prediction: Off
Number of isotope peaks used for i-FIT = 3

Monoisotopic Mass, Even Electron Ions
15 formula(e) evaluated with 1 results within limits (all results (up to 1000) for each mass)
Elements Used:
C: 0-22 H: 0 -30 O: 0 -5 Na: 0 -1

Minimum:				-1.5				
Maximum:		100.0	5.0	100.0				
Mass	Calc. Mass	mDa	PPM	DBE	i-FIT	Norm	Conf(%)	Formula
349.1415	349.1416	-0.1	-0.3	9.5	1041.3	n/a	n/a	C20 H22 O4 Na

Figure S53. HRESIMS spectrum of **14**

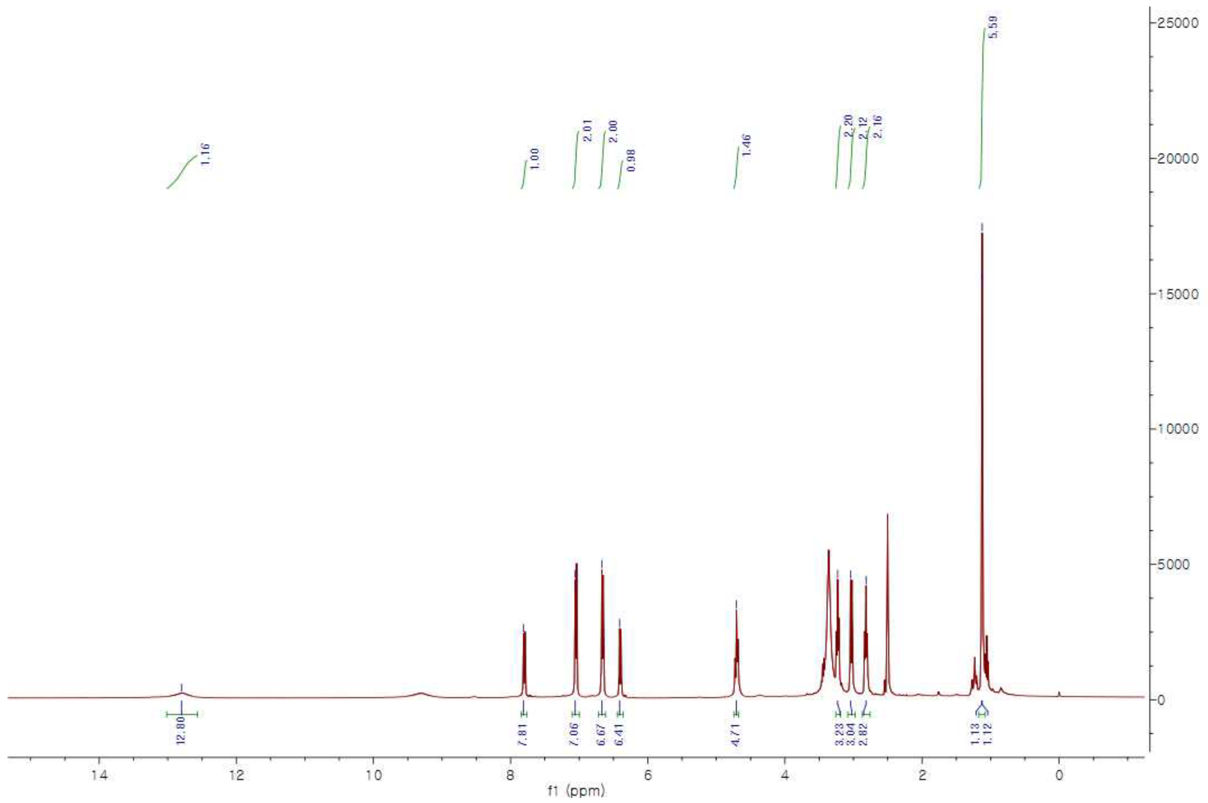

Figure S54. ^{1}H-NMR (400 MHz, DMSO-d_6) spectrum of **15**

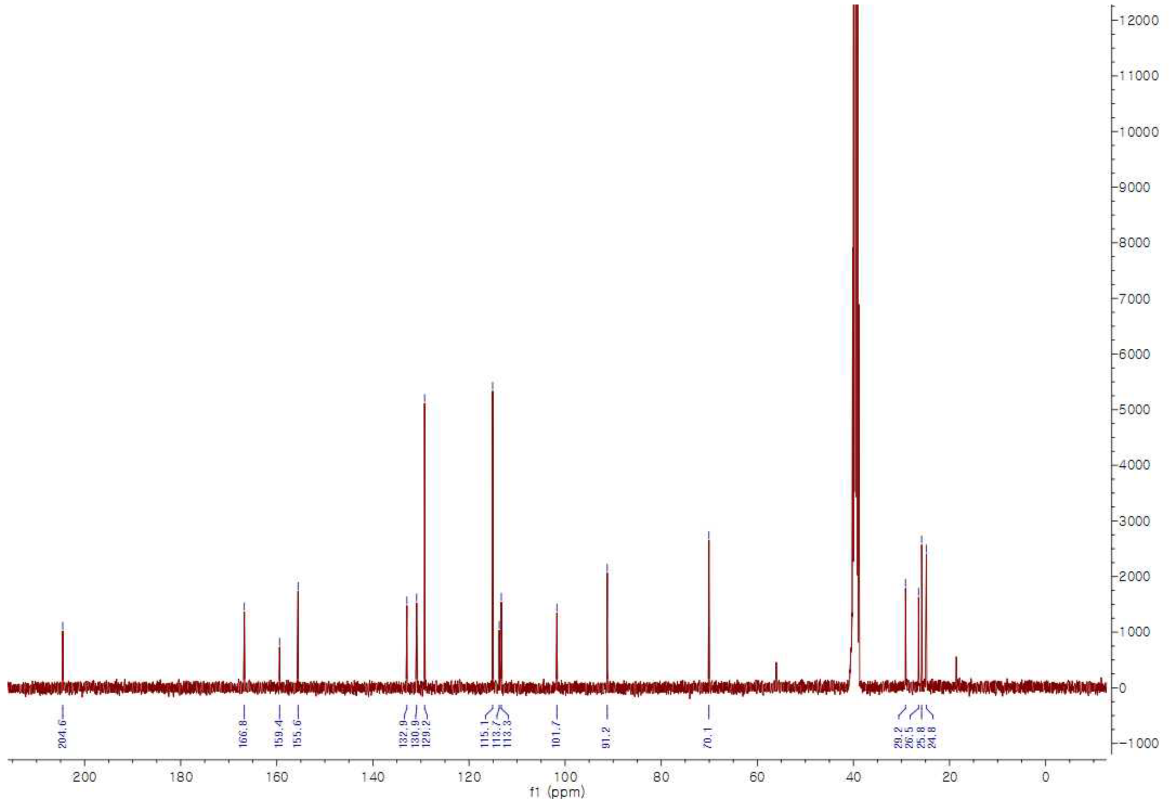

Figure S55. ^{13}C-NMR (100 MHz, DMSO-d_6) spectrum of **15**

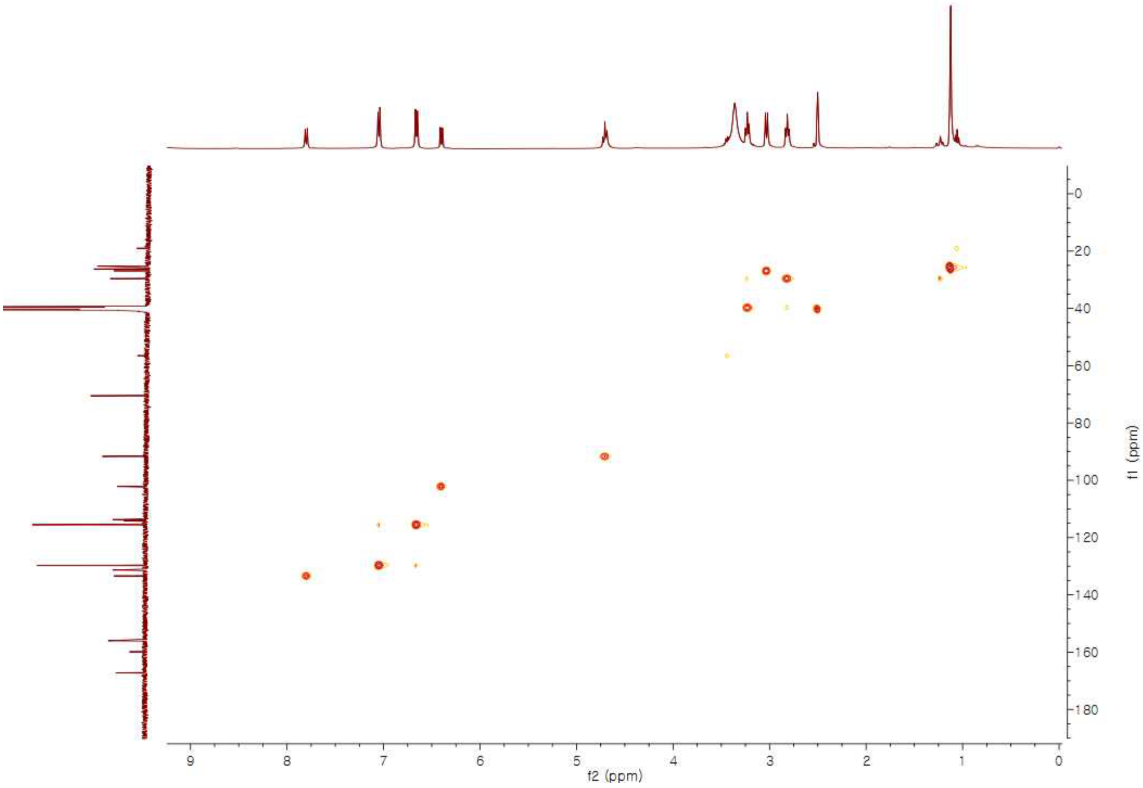

Figure S56. HSQC spectrum of **15**

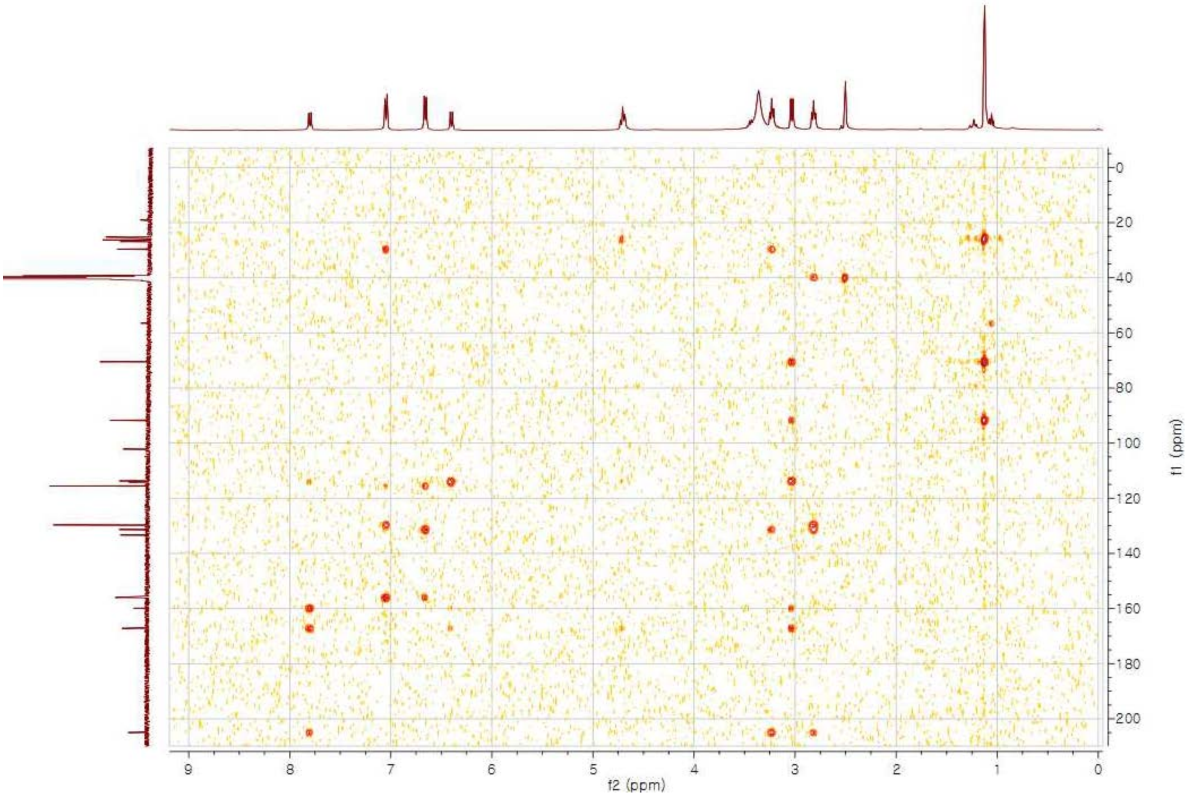

Figure S57. HMBC spectrum of **15**

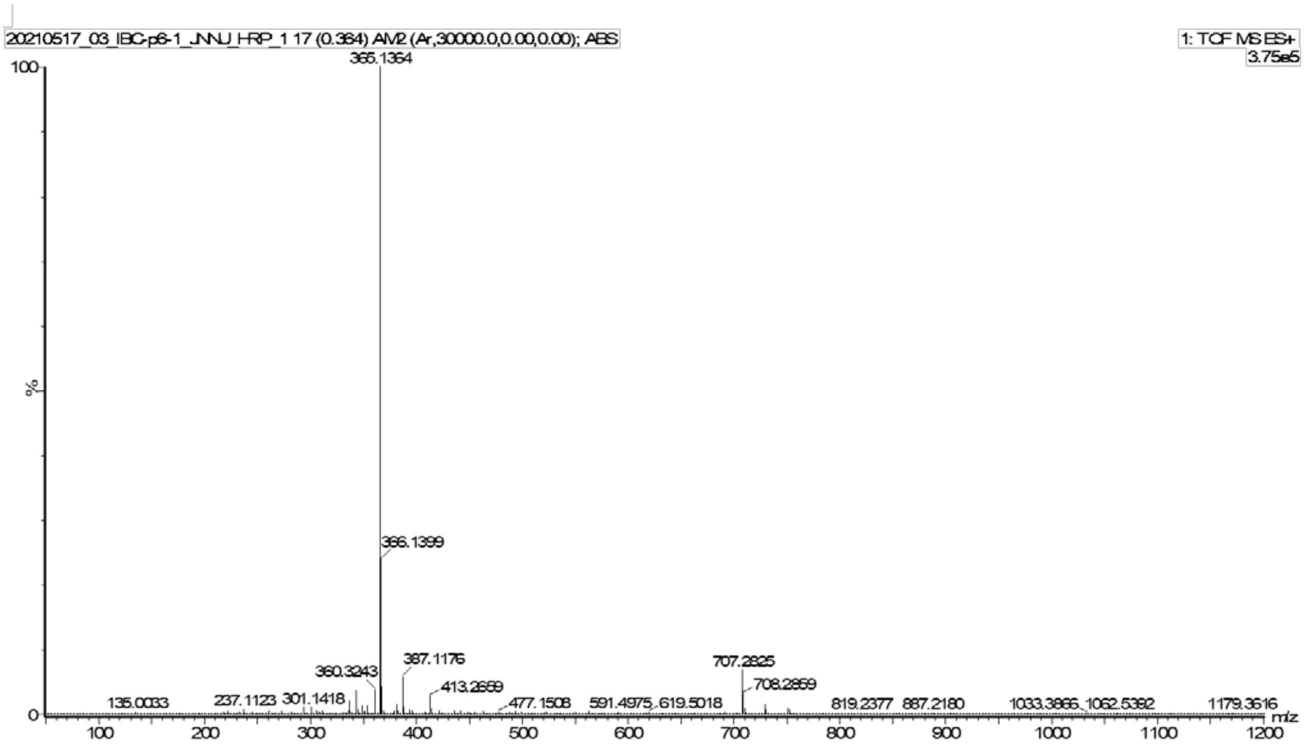

Elemental Composition Report

Single Mass Analysis
Tolerance = 5.0 PPM / DBE: min = -1.5, max = 100.0
Element prediction: Off
Number of isotope peaks used for i-FIT = 3

Monoisotopic Mass, Even Electron Ions
11 formula(e) evaluated with 1 results within limits (all results (up to 1000) for each mass)
Elements Used:
C: 0-22 H: 0 -30 O: 0 -5 Na: 0 -1

Minimum:				-1.5				
Maximum:		100.0	5.0	100.0				
Mass	Calc. Mass	mDa	PPM	DBE	i-FIT	Norm	Conf(%)	Formula
365.1364	365.1365	-0.1	-0.3	9.5	925.7	n/a	n/a	C20 H22 O5 Na

Figure S58. HRESIMS spectrum of **15**

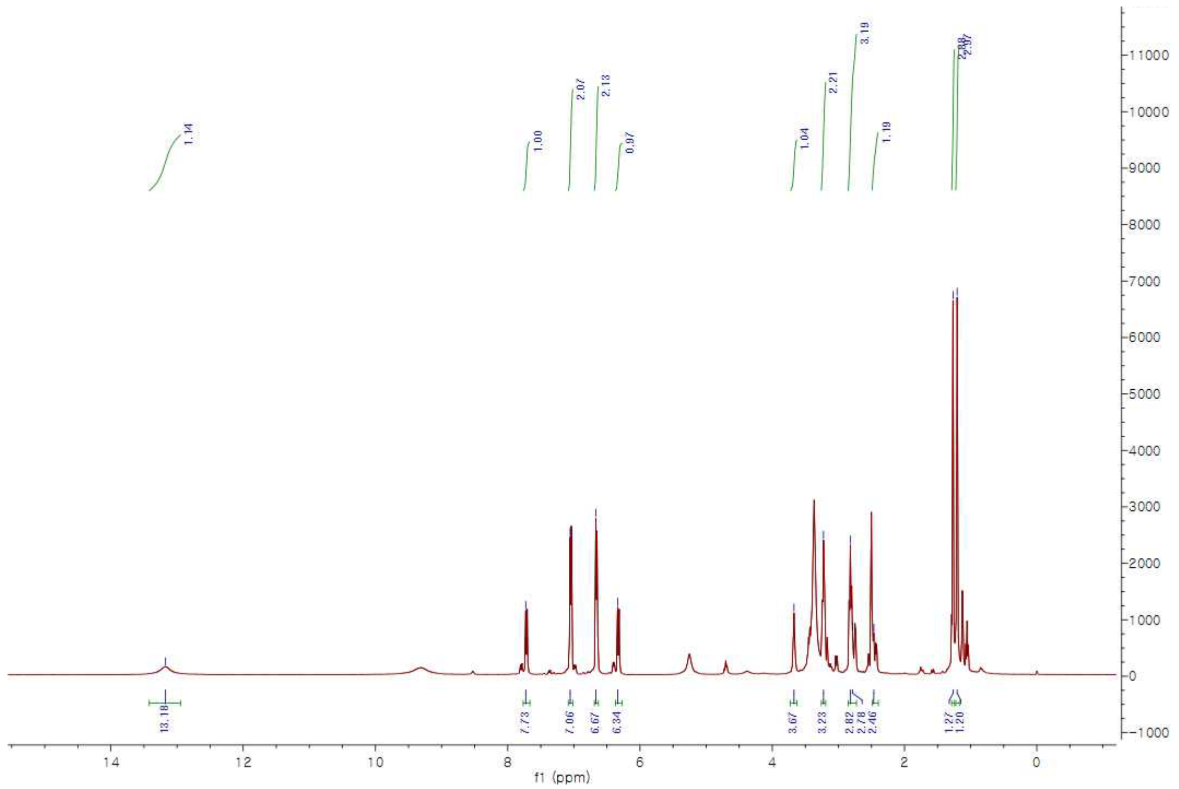

Figure S59. ^{1}H-NMR (400 MHz, DMSO-d_6) spectrum of **16**

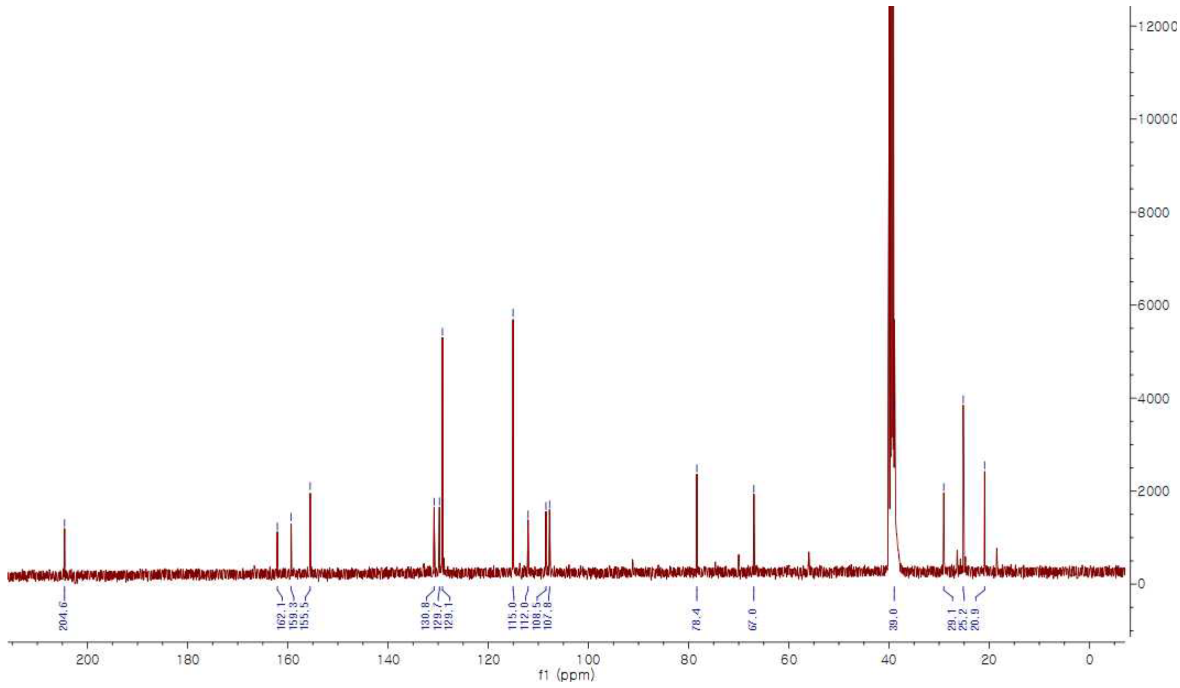

Figure S60. ^{13}C-NMR (100 MHz, DMSO-d_6) spectrum of **16**

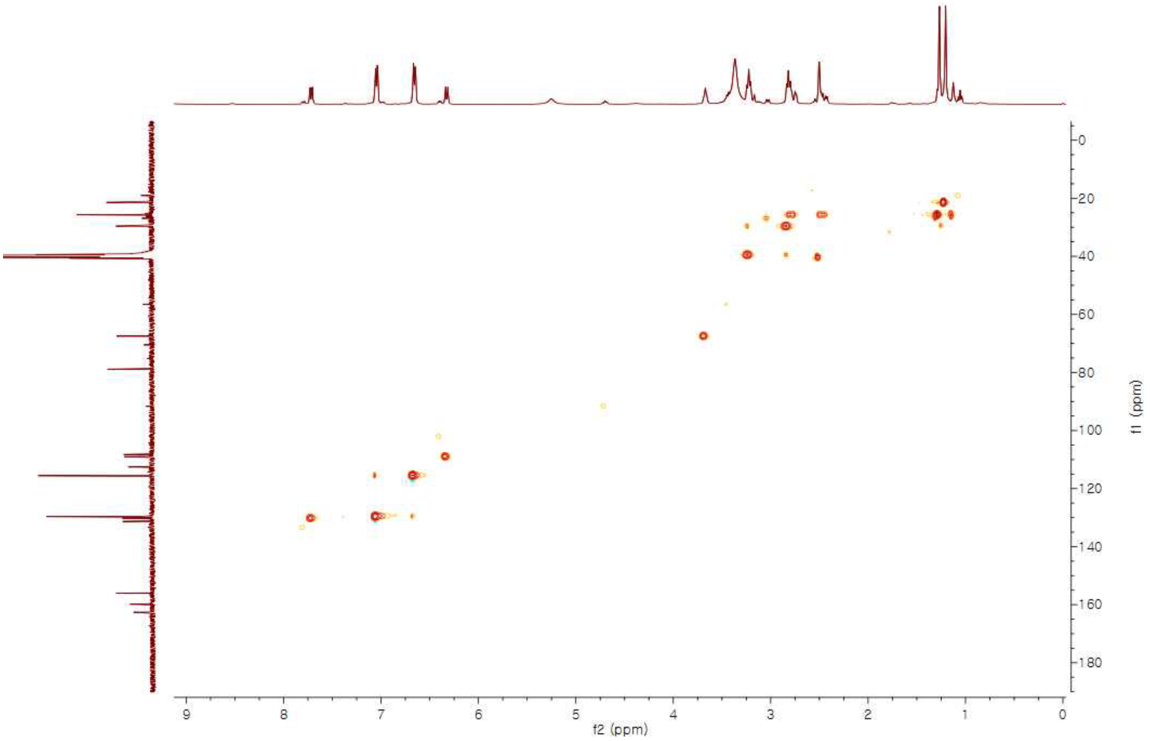

Figure S61. HSQC spectrum of **16**

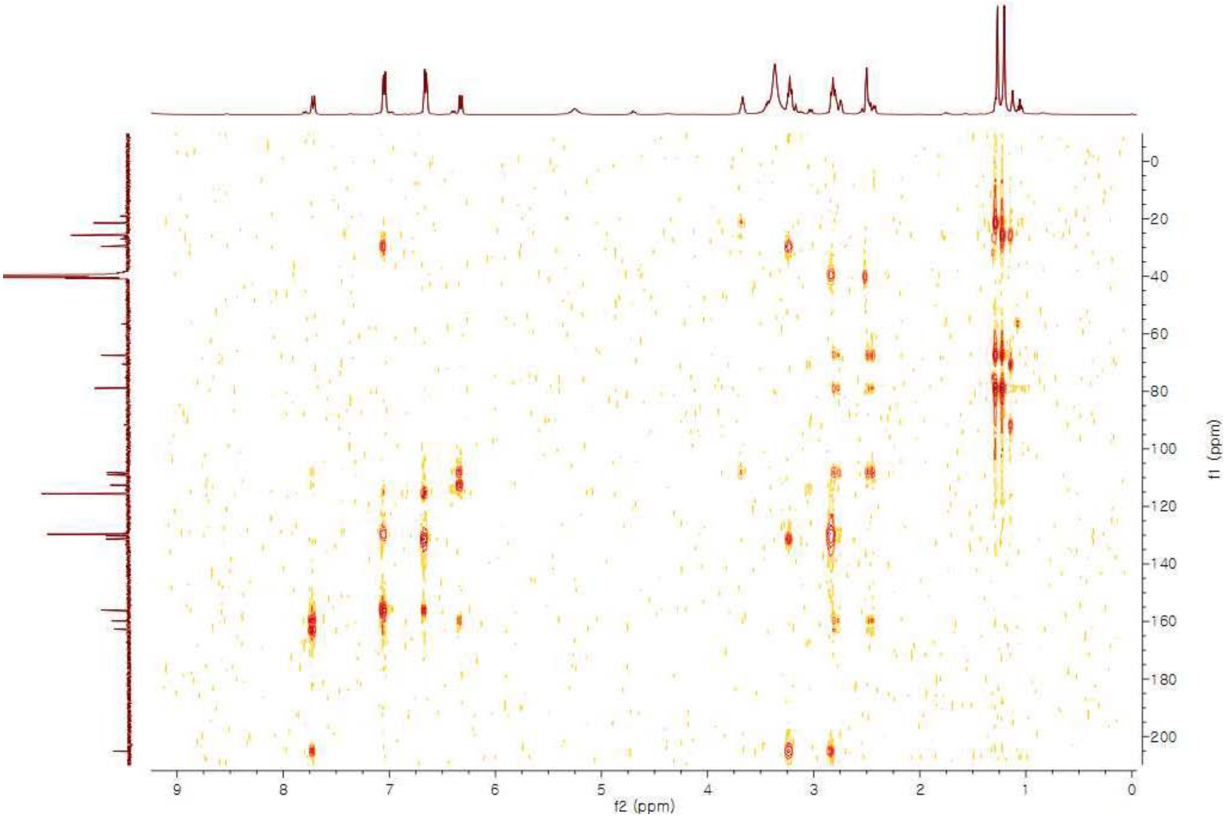

Figure S62. HMBC spectrum of **16**

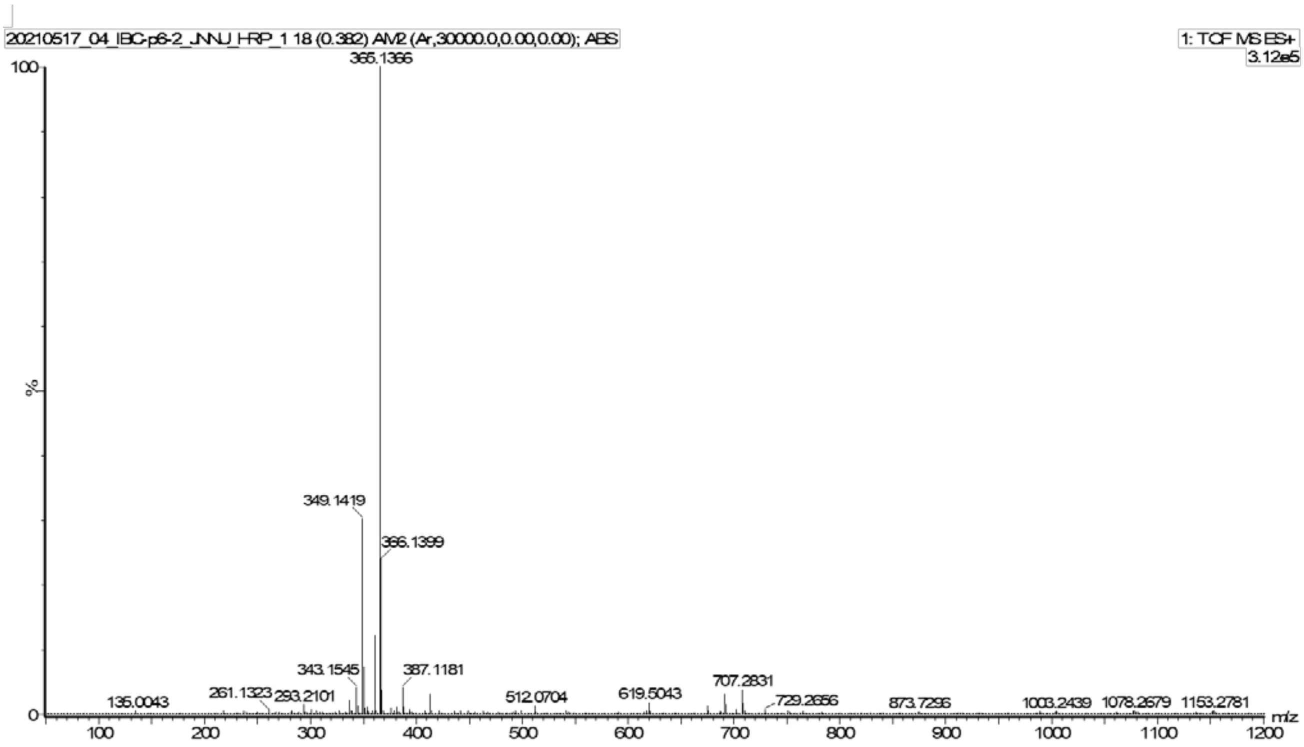

Elemental Composition Report

Single Mass Analysis
Tolerance = 5.0 PPM / DBE: min = -1.5, max = 100.0
Element prediction: Off
Number of isotope peaks used for i-FIT = 3

Monoisotopic Mass, Even Electron Ions
11 formula(e) evaluated with 1 results within limits (all results (up to 1000) for each mass)
Elements Used:
C: 0-22 H: 0 -30 O: 0 -5 Na: 0 -1

Minimum:				-1.5				
Maximum:		100.0	5.0	100.0				
Mass	Calc. Mass	mDa	PPM	DBE	i-FIT	Norm	Conf(%)	Formula
365.1366	365.1365	0.1	0.3	9.5	932.9	n/a	n/a	C20 H22 O5 Na

Figure S63. HRESIMS spectrum of **16**

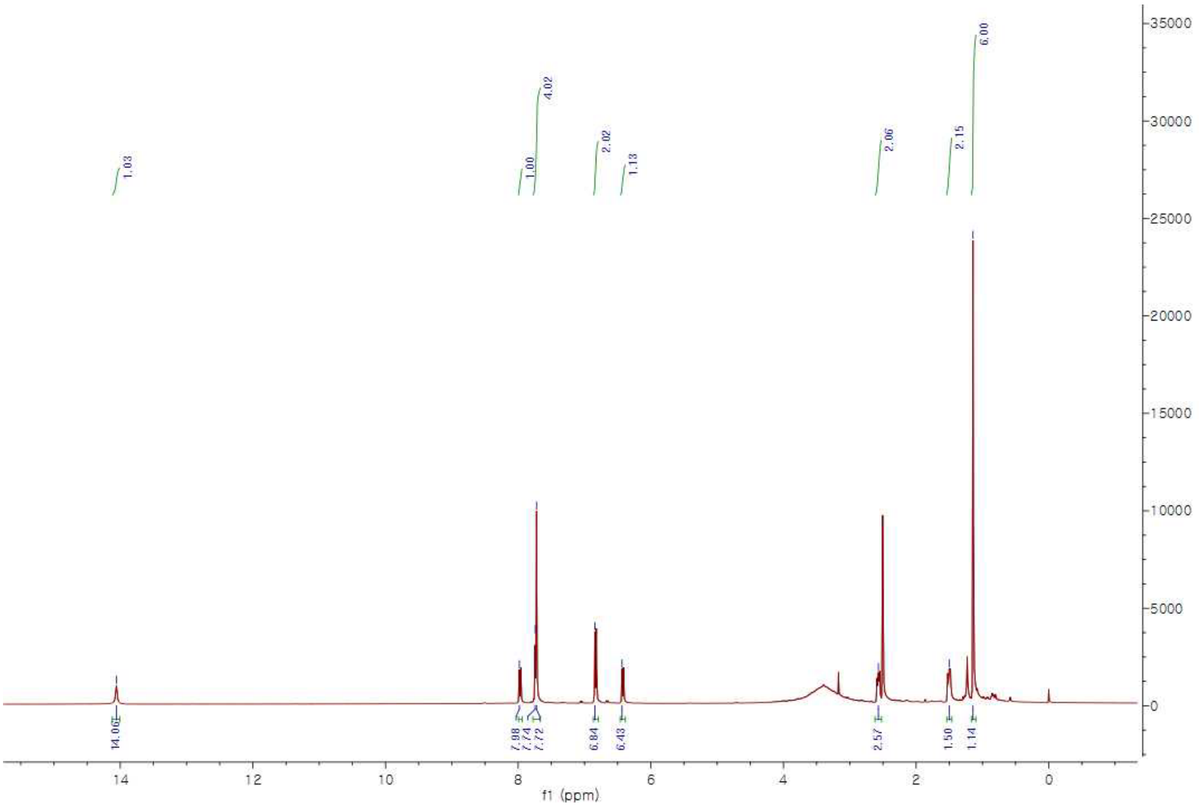

Figure S64. ^{1}H-NMR (400 MHz, DMSO-d_6) spectrum of **17**

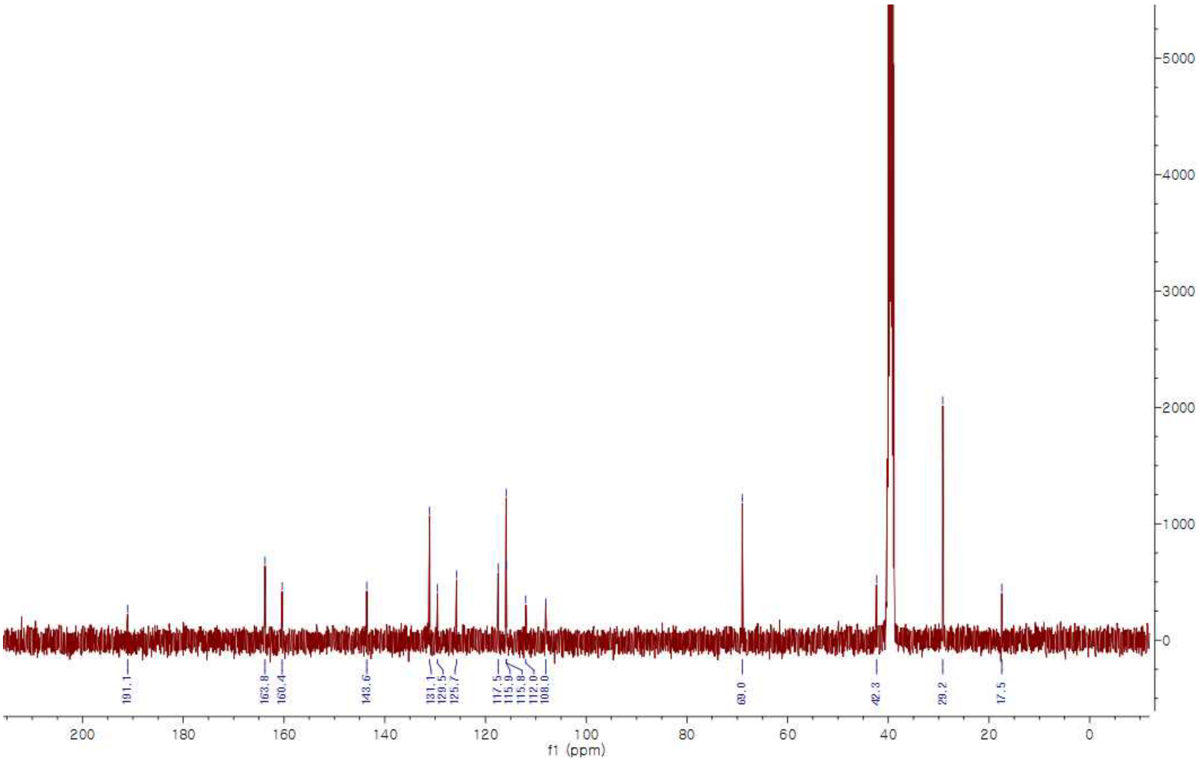

Figure S65. ^{13}C-NMR (100 MHz, DMSO-d_6) spectrum of **17**

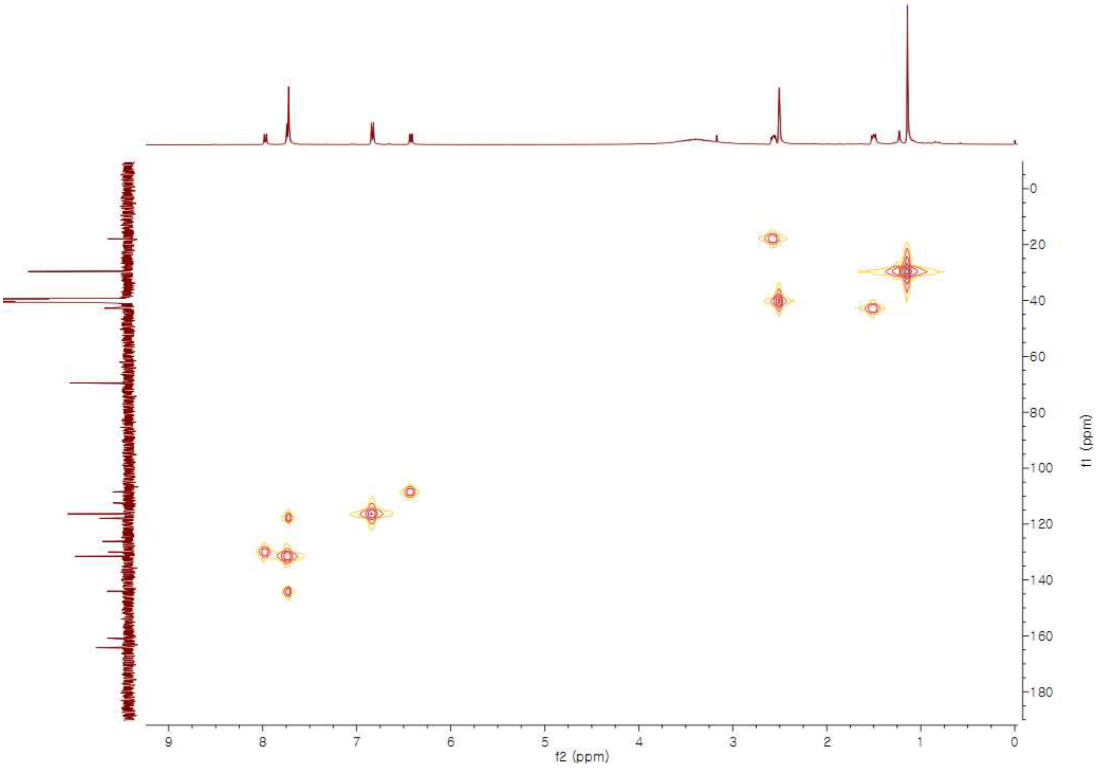

Figure S66. HSQC spectrum of **17**

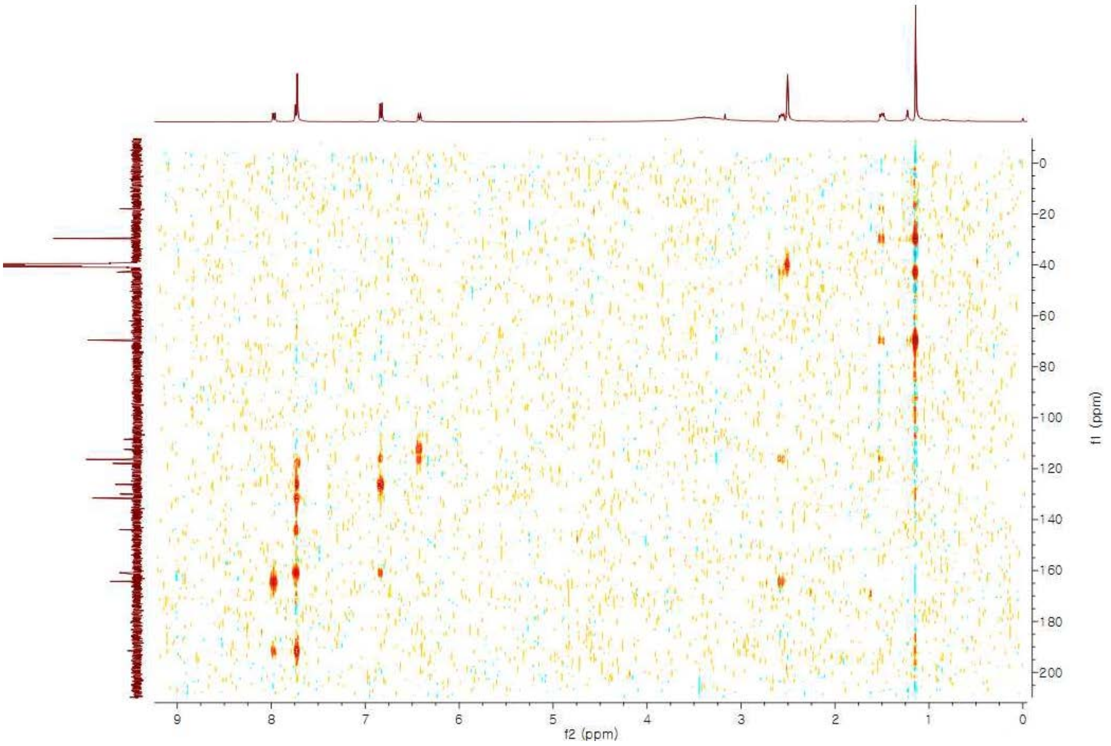

Figure S67. HMBC spectrum of **17**

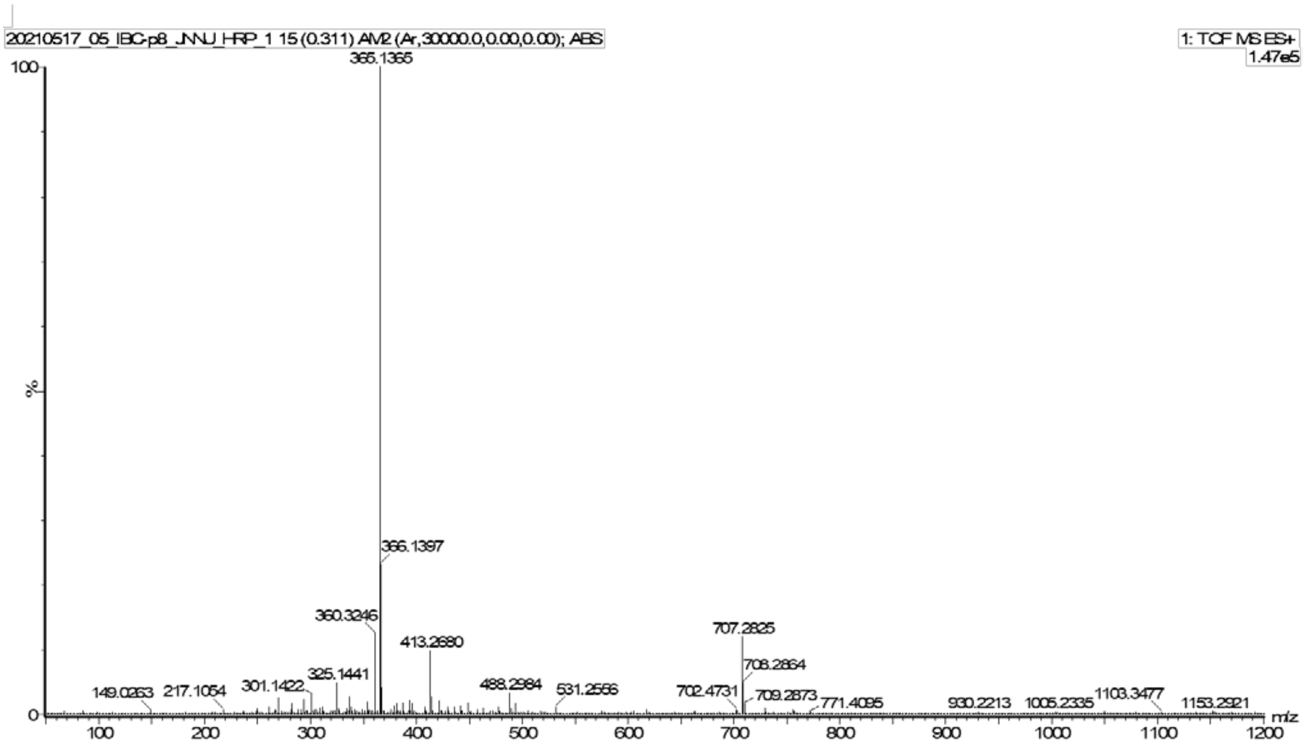

Elemental Composition Report

Single Mass Analysis
Tolerance = 5.0 PPM / DBE: min = -1.5, max = 100.0
Element prediction: Off
Number of isotope peaks used for i-FIT = 3

Monoisotopic Mass, Even Electron Ions
11 formula(e) evaluated with 1 results within limits (all results (up to 1000) for each mass)
Elements Used:
C: 0-22 H: 0 -30 O: 0 -5 Na: 0 -1

Minimum:				-1.5				
Maximum:		100.0	5.0	100.0				
Mass	Calc. Mass	mDa	PPM	DBE	i-FIT	Norm	Conf(%)	Formula
365.1365	365.1365	0.0	0.0	9.5	875.6	n/a	n/a	C20 H22 O5 Na

Figure S68. HRESIMS spectrum of **17**

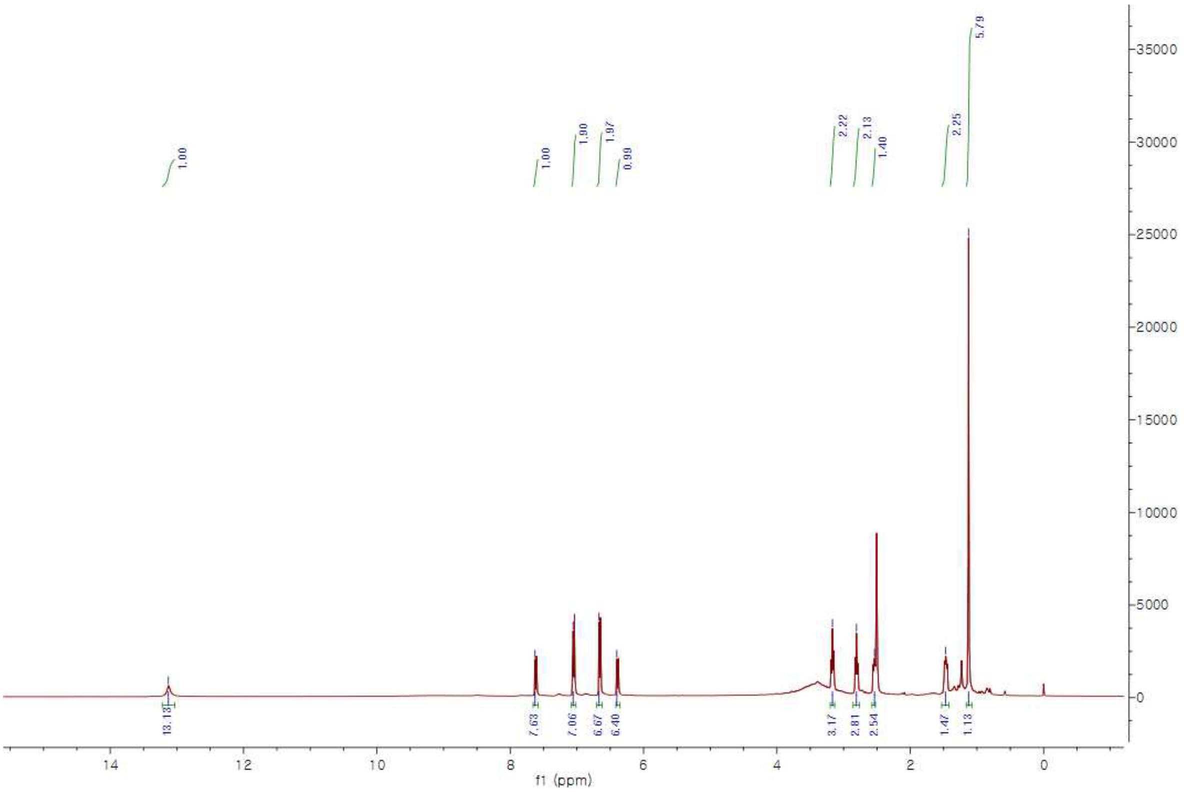

Figure S69. ^{1}H-NMR (400 MHz, DMSO-d_6) spectrum of **19**

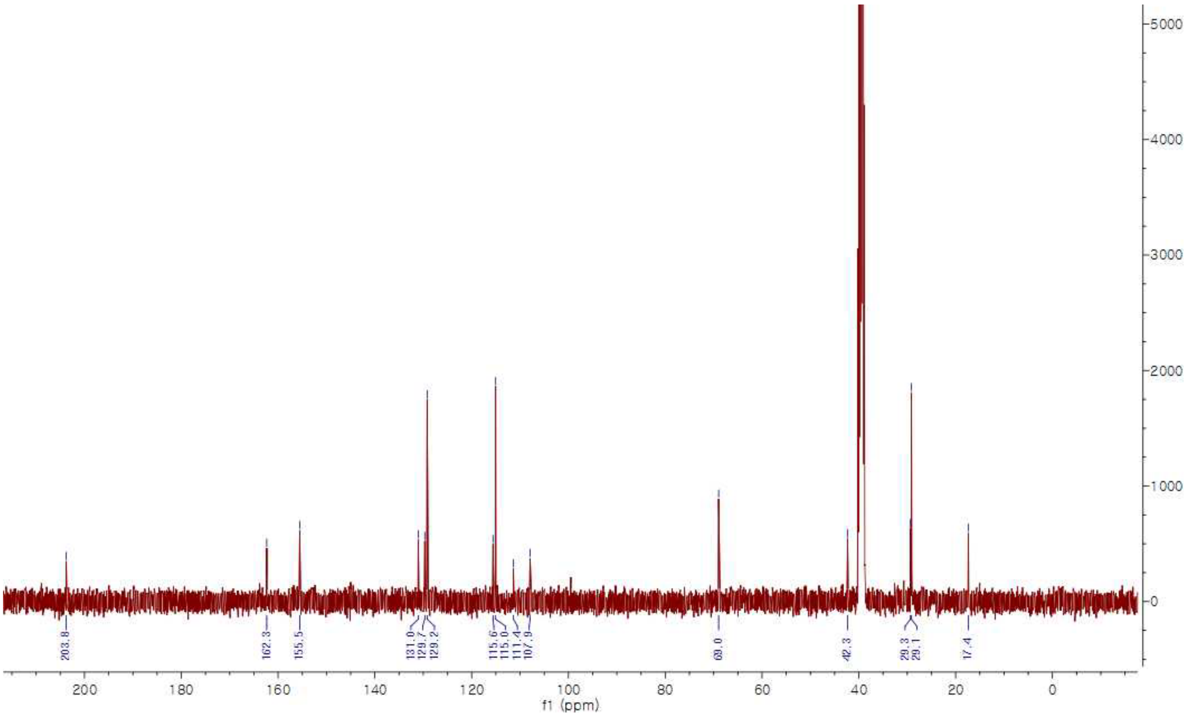

Figure S70. ^{13}C-NMR (100 MHz, DMSO-d_6) spectrum of **19**

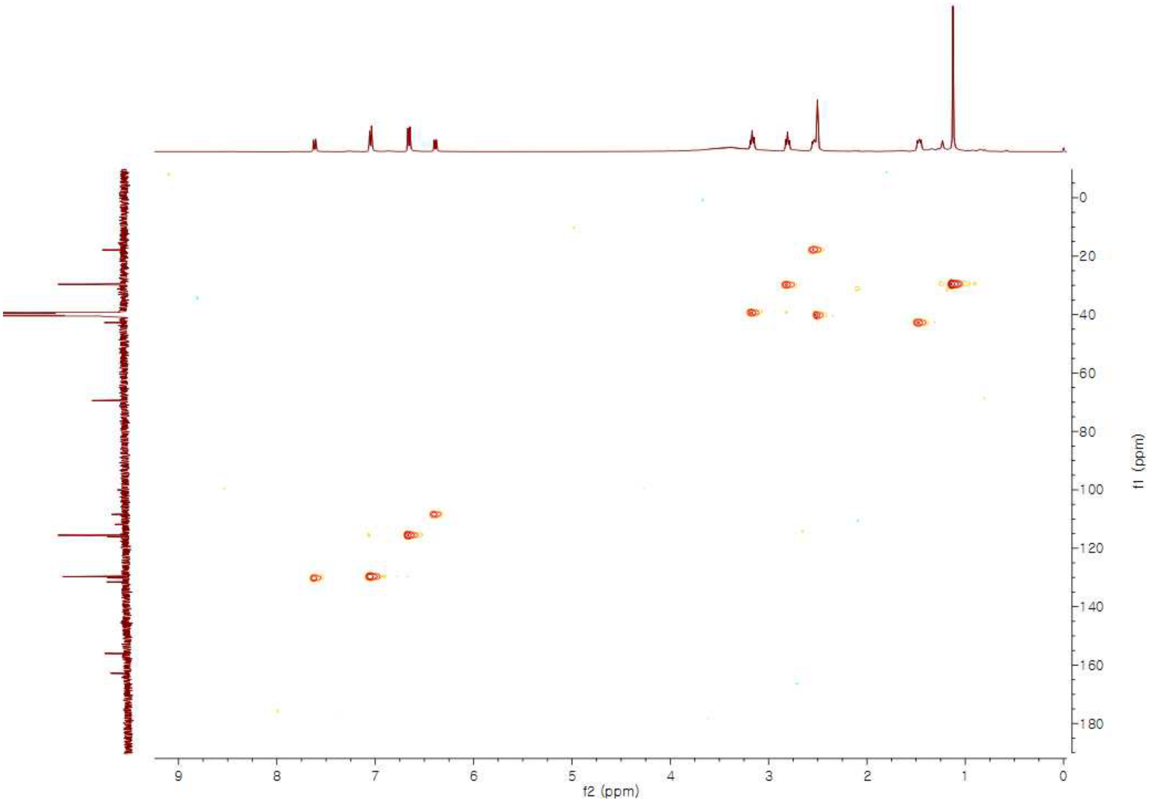

Figure S71. HSQC spectrum of **19**

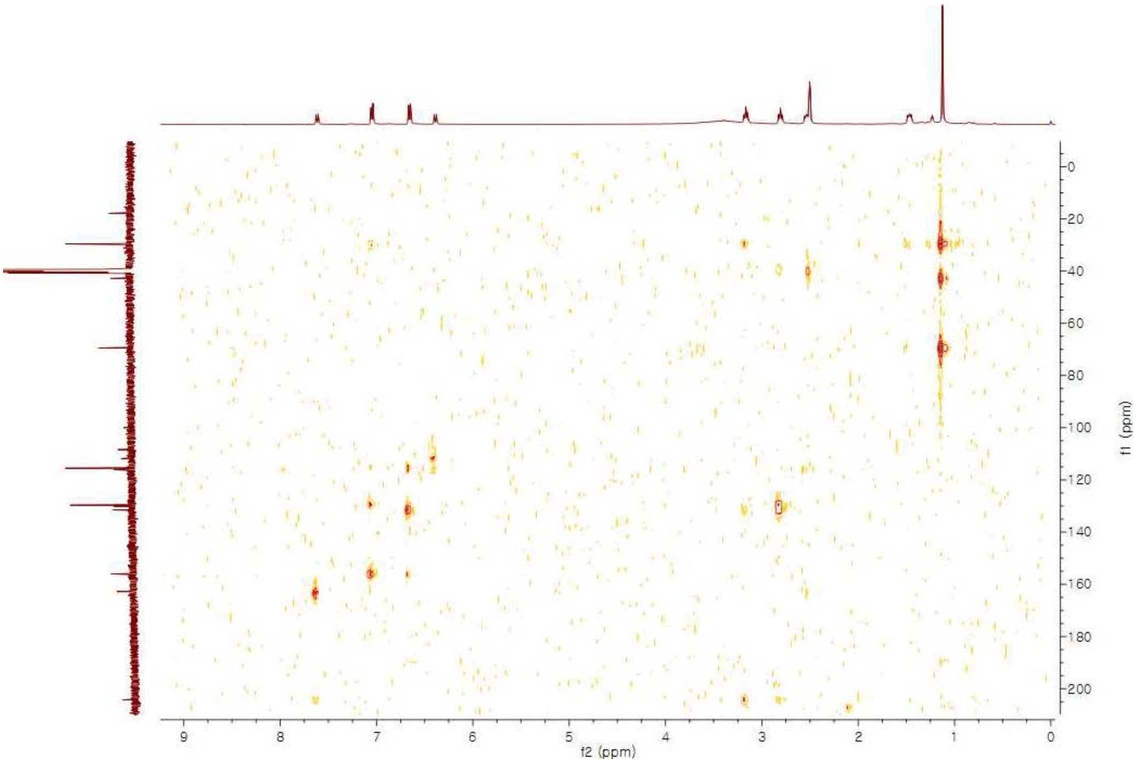

Figure S72. HMBC spectrum of **19**

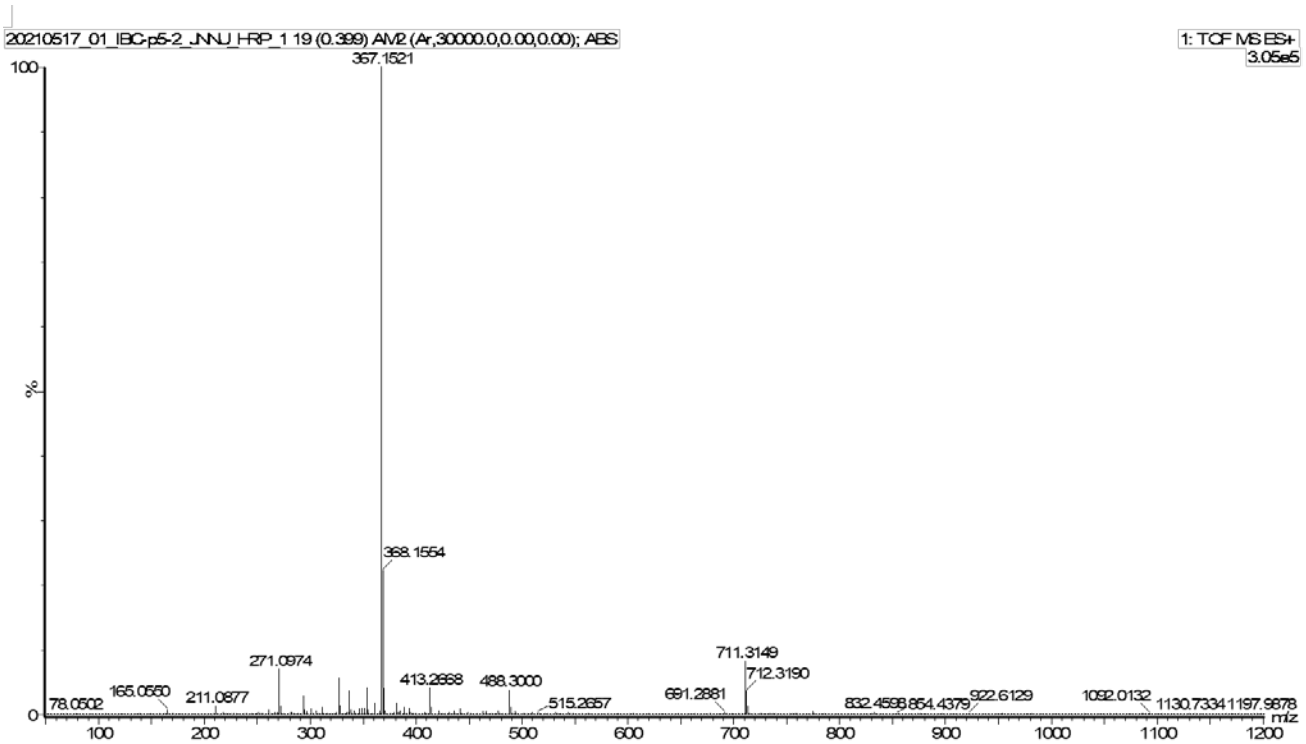

Elemental Composition Report

Single Mass Analysis
Tolerance = 5.0 PPM / DBE: min = -1.5, max = 100.0
Element prediction: Off
Number of isotope peaks used for i-FIT = 3

Monoisotopic Mass, Even Electron Ions
11 formula(e) evaluated with 1 results within limits (all results (up to 1000) for each mass)
Elements Used:
C: 0-22 H: 0 -30 O: 0 -5 Na: 0 -1

Minimum:				-1.5				
Maximum:		100.0	5.0	100.0				
Mass	Calc. Mass	mDa	PPM	DBE	i-FIT	Norm	Conf(%)	Formula
367.1521	367.1521	0.0	0.0	8.5	932.4	n/a	n/a	C20 H24 O5 Na

Figure S73. HRESIMS spectrum of **19**

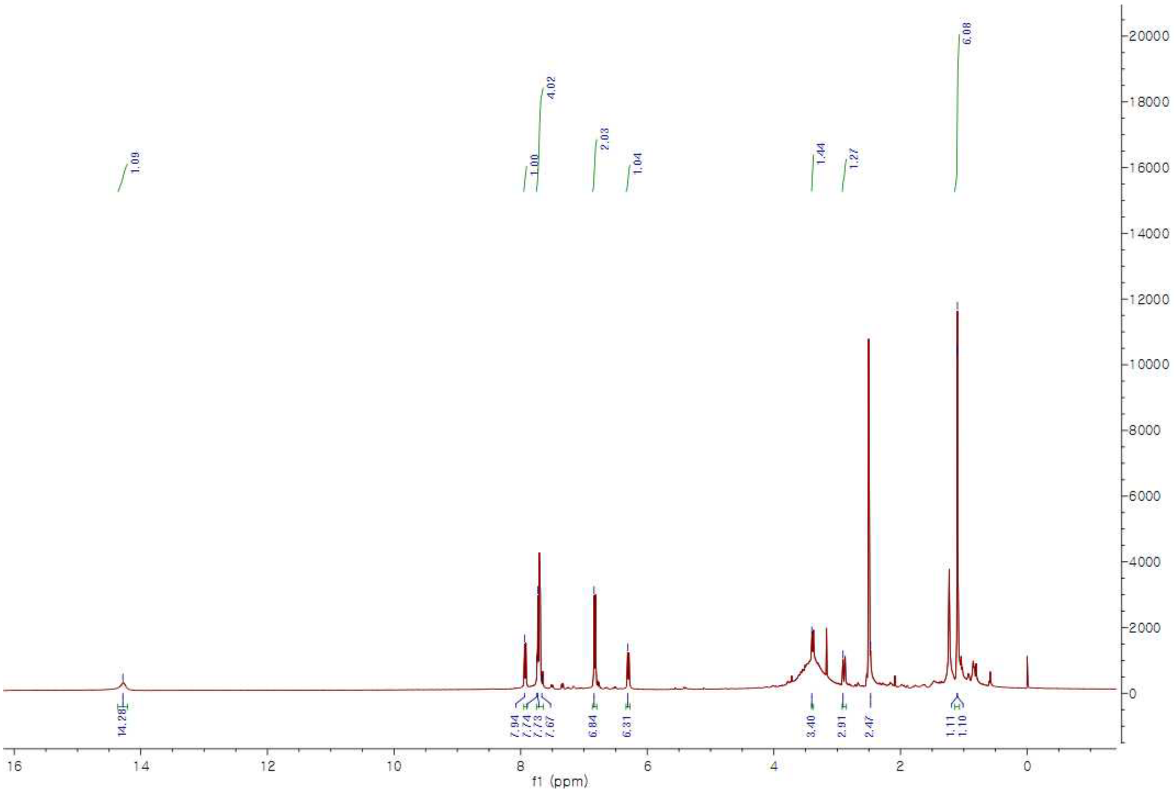

Figure S74. ^{1}H-NMR (400 MHz, DMSO-d_6) spectrum of **18**

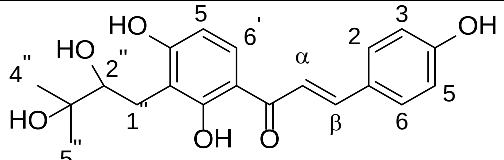 **18**	^{1}H-NMR (DMSO-d_6, 400 MHz, δ in ppm, J in Hz) δ 14.28 (OH), 7.94 (1H, d, J = 9.1, H-2′), 7.74~7.67 (4H, overlapped, H-α,β,2,6), 6.84 (2H, d, J = 8.5, H-3,5), 6.31 (1H, d, J = 9.1, H-5′), 3.40 (1H, dd, J = 9.7, 2.1, H-2′′), 2.91 (1H, dd, J = 13.5, 2.1, H-1′′a), 2.47 (1H, overlapped, H-1′′b), 1.11 (3 H, s, H-4′′), 1.10 (3H, s, H-5′′).

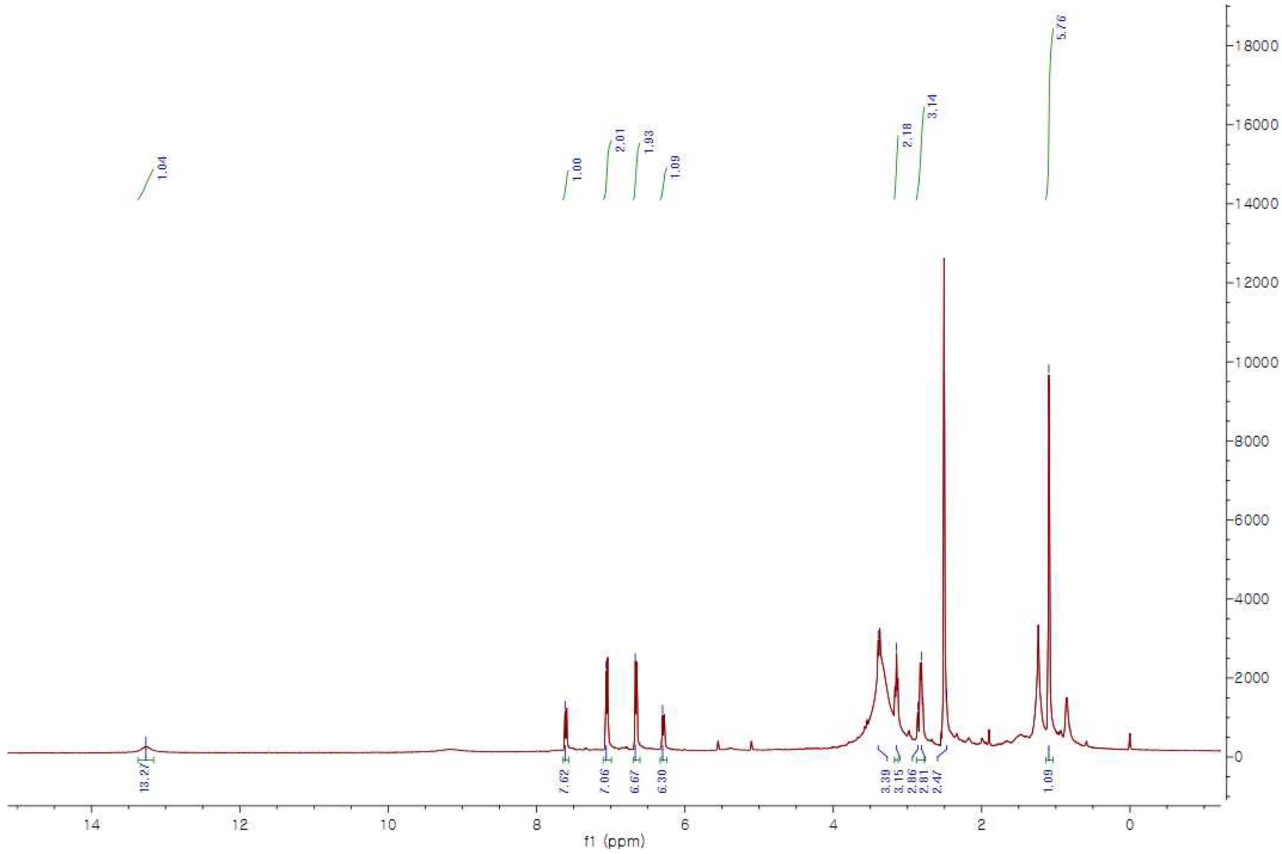

Figure S75. ^{1}H-NMR (500 MHz, DMSO-d_6) spectrum of **20**

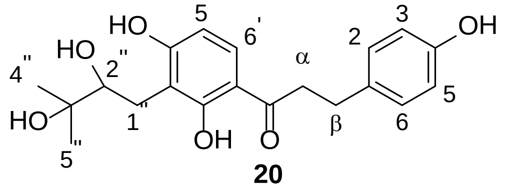 **20**	^{1}H-NMR (DMSO-d_6, 400 MHz, δ in ppm, *J* in Hz) δ 13.27 (OH), 7.62 (1H, d, *J* = 9.1, H-6′), 7.06 (2H, d, *J* = 7.9, H-2, 6), 6.67 (2H, d, *J* = 7.9, H-3,5), 6.30 (1H, d, *J* = 9.1, H-5′), 3.39 (1H, overlapped, H-2′′), 3.15 (2H, t, *J* = 7.5, H-α), 2.86 (1H, m, H-1′′a), 2.81 (2H, t, *J* = 7.5, H-β), 2.47 (1H, o verlapped, H-1′′b), 1.09 (6H, s, H-4′′,5′′).

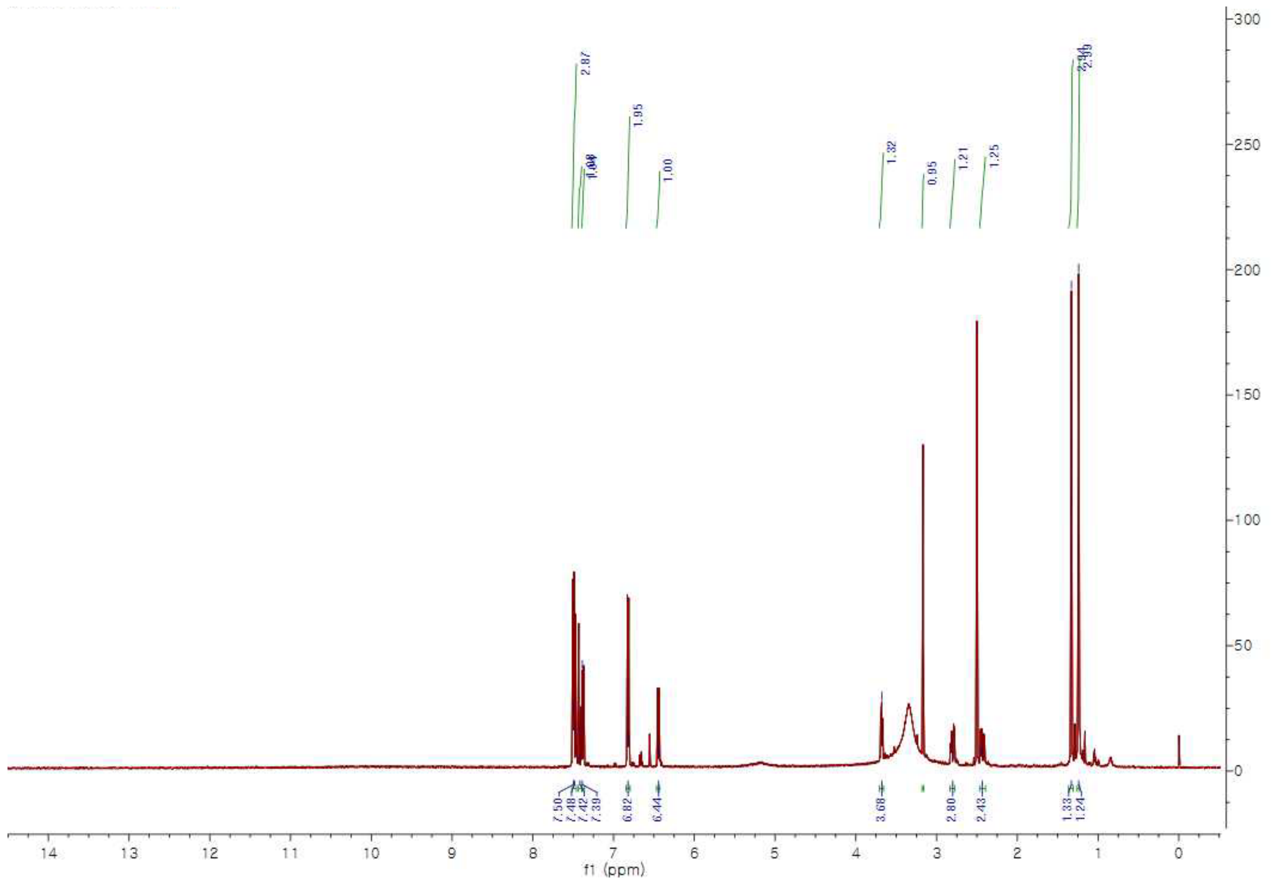

Figure S76. ^{1}H-NMR (400 MHz, DMSO-d_6) spectrum of **21**

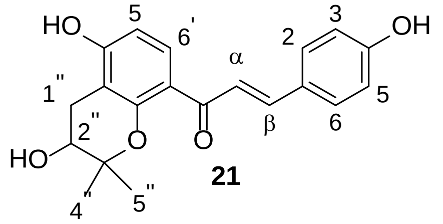	^{1}H-NMR (DMSO-d_6, 400 MHz, δ in ppm, *J* in Hz) δ 7.50 (2H, d, *J* = 8.9, H-2,6), 7.48 (1H, d, *J* = 15.8, H-β), 7.42 (1H, d, *J* = 15.8, H-α), 7.39 (1H, d, *J* = 8.6, H-6′), 6.82 (2H, d, *J* = 8.6, H-3, 5), 6.44 (1H, d, *J* = 8.6, H-5′), 3.68 (1H, m, H-2′′), 2.80 (1H, dd, *J* = 17.2, 5.4, H-1′′a), 2.43 (1H, dd, *J* = 17.2, 7.5, H-1′′b), 1.33 (3H, s, H-4′′), 1.24 (3H, s, H-5′′).
